# Supplementary material for: Circadian regulation of the transcriptome in a complex polyploid crop
Source: PLoS Biol. 2022 Oct 13;20(10):e3001802. doi: 10.1371/journal.pbio.3001802 (PMC9560141; doi:10.1371/journal.pbio.3001802)
Supplement: S1 Note — (DOCX) [file pbio.3001802.s001.docx]

# S1_Note: Proportions of rhythmic genes in expression datasets

The proportion of the wheat transcriptome categorized as rhythmic depends on the window over which measurements are taken, the *q*-value cut-off for rhythmicity and the threshold at which you exclude low expression transcripts. We chose to conduct our primary analysis without filtering for any minimum expression to allow us to capture all rhythmically categorized transcripts. This approach has been used in several previous studies (1–3) and comparison of relative proportions in *Arabidopsis* and wheat is possible as identical criteria are applied to both datasets as shown in Table 1 in the main text. To assess the effects of applying a pre-filtering step to exclude low-expression genes, we removed genes with expression less than 0.1 TPM in six or more timepoints and again ran Metacycle to define numbers of rhythmic genes. This had the effect of reducing the number of genes defined as expressed (and therefore the proportions of rhythmic genes) but had only a small effect on the numbers of rhythmically categorized genes as shown in S1_Table. With the filter applied to the wheat 24-68h dataset, the number of rhythmic genes reduced by 206 genes at q<0.05, and increased by 752 genes at q<0.01 which had a minimal impact on mean period and relative amplitudes. However, the proportion of rhythmic genes increased from 33.56% to 46.3% in wheat and from 50.7% to 61.5% in *Arabidopsis* due to the lower expression totals. Regardless of whether a filtering step was applied or whether a q<0.05 or q<0.01 threshold was used, the proportion of genes in *Arabidopsis* was found to be consistently higher than in wheat (*p* < 0.001, one-tailed, two-proportions z-test).

1. Romanowski A, Schlaen RG, Perez-Santangelo S, Mancini E, Yanovsky MJ. Global transcriptome analysis reveals circadian control of splicing events in Arabidopsis thaliana. Plant J [Internet]. 2020 Jul 1 [cited 2021 Jun 21];103(2):889–902. Available from: https://onlinelibrary.wiley.com/doi/full/10.1111/tpj.14776

2. Covington MF, Maloof JN, Straume M, Kay SA, Harmer SL. Global transcriptome analysis reveals circadian regulation of key pathways in plant growth and development. Genome Biol [Internet]. 2008 Aug 18 [cited 2021 Jul 7];9(8):1–18. Available from: https://genomebiology.biomedcentral.com/articles/10.1186/gb-2008-9-8-r130

3. Kim JA, Shim D, Kumari S, Jung HE, Jung KH, Jeong H, et al. Transcriptome analysis of diurnal gene expression in Chinese cabbage. Genes (Basel) [Internet]. 2019 [cited 2021 Jul 7];10(2). Available from: /pmc/articles/PMC6409912/
