# Supplementary material for: Circadian regulation of the transcriptome in a complex polyploid crop
Source: PLoS Biol. 2022 Oct 13;20(10):e3001802. doi: 10.1371/journal.pbio.3001802 (PMC9560141; doi:10.1371/journal.pbio.3001802)
Supplement: S2 Note — (DOCX) [file pbio.3001802.s002.docx]

# S2_Note: Mean period lengths in rhythmic wheat transcripts over sliding windows of circadian experiment

We wanted to investigate how period length changed over the time-course following transfer to constant light (L:L). Mean period lengths were calculated for rhythmic transcripts (B.H *q* < 0.01) over a sliding window of 0-44h, 12-56h or 24-68h relative to transfer to L:L. Unexpectedly, the mean period length of all rhythmic wheat genes at 0-44h was even longer at 28.61h (SD=3.421), which shortened over a window of 12-56h to 27.31h (SD=3.461) and finally shortened again to 26.82h (SD=3.208) over 24-68h. Differences in period length were statistically significant between each group (F(2, 52898) = 1041.432, p<0.001, 0-44h vs 12-56h (95% C.I.= -1.39 to -1.21), 0-44h vs 24-68h (95% C.I.= -1.88 to -1.70), 12-56h vs 24-68h (95% C.I.= -0.56 to -0.40), One-way ANOVA, Tukey HSD). This suggests that the period length of rhythmic genes entrained under 24h days became much longer immediately after transfer to constant light (L:L) and then progressively got shorter again over the following two days. There was no correlation between period length of individual genes measured in the first window versus the last window (R^2^ =0.02, *p-*value =0.06) therefore genes defined as ‘short’ and ‘long” period under 24-68h were not consistently short or long immediately after transfer to L:L.
