# Supplementary material for: Circadian regulation of the transcriptome in a complex polyploid crop
Source: PLoS Biol. 2022 Oct 13;20(10):e3001802. doi: 10.1371/journal.pbio.3001802 (PMC9560141; doi:10.1371/journal.pbio.3001802)
Supplement: S3 Note — (DOCX) [file pbio.3001802.s003.docx]

# S3_Note: Distribution of phases across different period bins for rhythmic wheat transcripts

Using the 24-68h data window, we split genes into 5 groups based on their period length; Group A: 21-23h (808 genes), Group B: 23-25h (2229 genes), Group C: 25-27h (2506 genes), Group D: 27-29h (1940 genes), Group E: 30-31h (761 genes). Genes in group C (25-27h) had the highest rhythmicity (defined by q-values and RAE scores) and largest relative amplitudes of the five groups. When we analysed histograms for phase of transcripts in each period bin, we found that groups A and B contained proportionally more dawn-peaking genes than the other groups, whereas groups D and E contained proportionally more dusk-peaking genes. These phase distributions can be seen in S4_Fig.
