# Supplementary material for: Circadian regulation of the transcriptome in a complex polyploid crop
Source: PLoS Biol. 2022 Oct 13;20(10):e3001802. doi: 10.1371/journal.pbio.3001802 (PMC9560141; doi:10.1371/journal.pbio.3001802)
Supplement: S4 Note — (DOCX) [file pbio.3001802.s004.docx]

# S4_Note: Examples of TF triads with imbalanced regulation

As case studies, we investigated two transcription factor triads with imbalanced circadian expression that make known contributions to the productivity of wheat. *WCBF2* (aka *TaCBF1*) participates in cold signal transduction in wheat and is induced by drought and low temperature gated by the circadian clock (Badawi et al., 2007; Fowler et al., 2005; Jaglo et al., 2001; Kume et al., 2005). The TCP transcription factor *PCF5* participates in cold tolerance and leaf development in rice and switchgrass (Xie et al., 2017; Yang et al., 2013). Interestingly, homologs of putative targets of WCBF2 *(WDHN13* and *WRAB17)* and TaPCF5 (*HY5, XTH27, NIA1, NIA2* and *OsPCNA)* also had imbalanced rhythmicity within their triads, although not necessarily with the same dominant homoeolog as their transcription factor (S7_Fig).

Badawi, M., Danyluk, J., Boucho, B., Houde, M., & Sarhan, F. (2007). The CBF gene family in hexaploid wheat and its relationship to the phylogenetic complexity of cereal CBFs. *Molecular Genetics and Genomics* , *277*(5), 533. https://doi.org/10.1007/S00438-006-0206-9

Fowler, S. G., Cook, D., & Thomashow, M. F. (2005). Low Temperature Induction of Arabidopsis CBF1, 2, and 3 Is Gated by the Circadian Clock. *Plant Physiology*, *137*(3), 961. https://doi.org/10.1104/PP.104.058354

Jaglo, K. R., Kleff, S., Amundsen, K. L., Zhang, X., Haake, V., Zhang, J. Z., Deits, T., & Thomashow, M. F. (2001). Components of the Arabidopsis C-repeat/dehydration-responsive element binding factor cold-response pathway are conserved in Brassica napus and other plant species. *Plant Physiology*, *127*(3), 910–917. https://doi.org/10.1104/pp.010548

Kume, S., Kobayashi, F., Ishibashi, M., Ohno, R., Nakamura, C., & Takumi, S. (2005). Differential and coordinated expression of Cbf and Cor/Lea genes during long-term cold acclimation in two wheat cultivars showing distinct levels of freezing tolerance. *Genes and Genetic Systems*. https://doi.org/10.1266/ggs.80.185

Xie, Q., Liu, X., Zhang, Y., Tang, J., Yin, D., Fan, B., Zhu, L., Han, L., Song, G., & Li, D. (2017). Identification and Characterization of microRNA319a and Its Putative Target Gene, PvPCF5, in the Bioenergy Grass Switchgrass (Panicum virgatum). *Frontiers in Plant Science*, *0*, 396. https://doi.org/10.3389/FPLS.2017.00396

Yang, C., Li, D., Mao, D., Liu, X., Ji, C., Li, X., Zhao, X., Cheng, Z., Chen, C., & Zhu, L. (2013). Overexpression of microRNA319 impacts leaf morphogenesis and leads to enhanced cold tolerance in rice (Oryza sativaL.). *Plant, Cell and Environment*, *36*(12), 2207–2218. https://doi.org/10.1111/PCE.12130/SUPPINFO
