# Supplementary material for: Circadian regulation of the transcriptome in a complex polyploid crop
Source: PLoS Biol. 2022 Oct 13;20(10):e3001802. doi: 10.1371/journal.pbio.3001802 (PMC9560141; doi:10.1371/journal.pbio.3001802)
Supplement: S5 Note — (DOCX) [file pbio.3001802.s005.docx]

# S5_Note: Detecting patterns of triad circadian balance

We looked at triads with imbalanced rhythmicity (one rhythmic gene q <0.01 (2058 triads) or two rhythmic genes q <0.01(1392 triads)) and triads with balanced rhythmicity or three rhythmic genes q<0.05 (3448 triads). For each gene, we found the corresponding chromosome positions based on the IWGSC RefSeq v1.1 gene annotation in cv. Chinese spring. In our dataset, we found 4225 triads with positional information and with either 1, 2 or 3 rhythmic homoeologs. The probability of a triads having 1 rhythmic homoeolog was 45%, 2 rhythmic homoeologs was 30% and 3 rhythmic homeologs was 25% based on their occurrence in the real data. Using the formula:

$$\left| \frac{\ln\left( n \right)}{\ln\left( P \right)} \right|$$

Where:

n = the total number of triads, P = the probability of each triad class occurring, ln = natural log and |…| = the absolute value

we could calculate the longest expected run of each category of rhythmic triads. Using the Wald–Wolfowitz test, we could also statistically test the randomness of each category to see whether rhythmic triads were grouping together more often than would be expected by chance. The null hypothesis is that all elements in a serial list are independent. The results, shown in S4_Table, suggest that sets of balanced and imbalanced triads are not grouped together in the genome and, likewise, that there are no regions in the genome where absence of rhythmicity from a particular chromosome is clustered more than would be expected by chance.
