# Supplementary material for: Circadian regulation of the transcriptome in a complex polyploid crop
Source: PLoS Biol. 2022 Oct 13;20(10):e3001802. doi: 10.1371/journal.pbio.3001802 (PMC9560141; doi:10.1371/journal.pbio.3001802)
Supplement: S6 Note — (DOCX) [file pbio.3001802.s006.docx]

# S6_Note: Orthologs co-expressed in similar phased modules of gene expression

We used Biomart tool in Ensembl plants to identify *Arabidopsis* orthologs for rhythmically expressed wheat genes in each expression module based on percentages of nucleotide identity >40%. We then compared these putative orthologs with *Arabidopsis* genes expressed in similar phased *Arabidopsis* modules to find co-expressed genes. In dawn-phased A9 and W9, we found 45 ribosomal protein orthologs comprising both large and small ribosomal subunits. Other co-expressed genes in these modules included *CHALCONE SYNTHASE (CHS)*, an anthocyanin biosynthesis enzyme also involved in salicylic acid defence; *PHOSPHATE STARVATION RESPONSE 1 (PHR1)*, involved in maintenance of photosynthesis under light stress and phosphate deficiency; *UV RESISTANCE 2* responsible for repairing UV radiation-induced DNA damage, and a heat shock protein, *HSP60* which ensures correct folding of proteins in mitochondria (1–4). This suggests that in both *Arabidopsis* and wheat, the circadian clock initiates similar changes in ribosome biosynthesis and biotic and abiotic defence in anticipation of dawn. Genes peaking in both W3 and A2 included several light-harvesting complex proteins and *PHYTOCHROME INTERACTING FACTOR (PIF5)* as well as a cytochrome P450 monooxygenase gene *CYP709B3* which has previously been shown to be circadian regulated in *Arabidopsis* and may protect the plant from transpiration-triggered salinity stress during the day (5,6). Genes co-expressed in W4 and A5 included several involved in auxin transport and signalling such as: the polar auxin transporter *AVP1*, *AUXIN F-BOX PROTEIN 5,* *AUXIN RESPONSE FACTOR 6,* *AUXIN SIGNALING F-BOX 2* and the endosomal sorting complex protein *CHMP1A* which ensures proper sorting of auxin carriers (7). Orthologs expressed in wheat and *Arabidopsis* modules W5 and A6 included: *SEVEN IN ABSENTIA 2* which has been shown to regulate ABA-mediated stomatal closure and drought tolerance in *Arabidopsis* (8), and *HYDROPEROXIDE LYASE 1* which has a role in responding to insect attack and mechanical wounding (9). Enriched MYB-TFs in W8 and W9 modules included *ASYMMETRIC LEAVES 1 (AS1)* involved in leaf morphogenesis, *GONIDIALESS A/ZUOTIN RELATED FACTOR A1* involved in plant growth, *G2-LIKE FLAVONOID REGULATOR (GFR*) involved in regulation of flavonoid biosynthesis and *LATE MERISTEM IDENTITY2* involved in transition from vegetative growth to flowering (*LMI2*). TFBS for MYB- and MYB-related-TFs were enriched in the W2 and W3 morning peaking modules and in night expressed modules W6, W7 and W8 in keeping with the broad phase range of the MYB TF expression.

1. Nilsson L, Lundmark M, Jensen PE, Nielsen TH. The Arabidopsis transcription factor PHR1 is essential for adaptation to high light and retaining functional photosynthesis during phosphate starvation. Physiol Plant [Internet]. 2012 Jan [cited 2021 Jul 26];144(1):35–47. Available from: https://pubmed.ncbi.nlm.nih.gov/21910737/

2. Dao TTH, Linthorst HJM, Verpoorte R. Chalcone synthase and its functions in plant resistance. Phytochem Rev [Internet]. 2011 Sep [cited 2021 Jul 26];10(3):397–412. Available from: /pmc/articles/PMC3148432/

3. Willing EM, Piofczyk T, Albert A, Winkler JB, Schneeberger K, Pecinka A. UVR2 ensures transgenerational genome stability under simulated natural UV-B in Arabidopsis thaliana. Nat Commun [Internet]. 2016 Dec 1 [cited 2021 Jul 26];7(1):1–9. Available from: https://www.nature.com/articles/ncomms13522

4. Hsu YW, Juan CT, Wang CM, Jauh GY. Mitochondrial Heat Shock Protein 60s Interact with What’s This Factor 9 to Regulate RNA Splicing of ccmF C and rpl2. Plant Cell Physiol [Internet]. 2019 Jan 1 [cited 2021 Jul 26];60(1):116–25. Available from: https://pubmed.ncbi.nlm.nih.gov/30289547/

5. Mao G, Seebeck T, Schrenker D, Yu O. CYP709B3, a cytochrome P450 monooxygenase gene involved in salt tolerance in Arabidopsis thaliana. BMC Plant Biol [Internet]. 2013 Oct 28 [cited 2021 Jul 23];13(1):1–13. Available from: https://bmcplantbiol.biomedcentral.com/articles/10.1186/1471-2229-13-169

6. Pan Y, Michael TP, Hudson ME, Kay SA, Chory J, Schuler MA. Cytochrome P450 monooxygenases as reporters for circadian-regulated pathways. Plant Physiol [Internet]. 2009 Jun [cited 2021 Jul 23];150(2):858–78. Available from: /pmc/articles/PMC2689971/

7. Spitzer C, Reyes FC, Buono R, Sliwinski MK, Haas TJ, Otegui MS. The ESCRT-Related CHMP1A and B Proteins Mediate Multivesicular Body Sorting of Auxin Carriers in Arabidopsis and Are Required for Plant Development. Plant Cell [Internet]. 2009 Apr 28 [cited 2021 Jul 23];21(3):749–66. Available from: https://academic.oup.com/plcell/article/21/3/749/6095237

8. Bao Y, Wang C, Jiang C, Pan J, Zhang G, Liu H, et al. The tumor necrosis factor receptor-associated factor (TRAF)-like family protein SEVEN IN ABSENTIA 2 (SINA2) promotes drought tolerance in an ABA-dependent manner in Arabidopsis. New Phytol [Internet]. 2014 Apr 1 [cited 2021 Jul 27];202(1):174–87. Available from: https://nph.onlinelibrary.wiley.com/doi/full/10.1111/nph.12644

9. Noordermeer MA, Veldink GA, Vliegenthart JFG. Fatty acid hydroperoxide lyase: A plant cytochrome P450 enzyme involved in wound healing and pest resistance. ChemBioChem. 2001;2(7–8):494–504.
