# Supplementary material for: Circadian regulation of the transcriptome in a complex polyploid crop
Source: PLoS Biol. 2022 Oct 13;20(10):e3001802. doi: 10.1371/journal.pbio.3001802 (PMC9560141; doi:10.1371/journal.pbio.3001802)
Supplement: S7 Note — (DOCX) [file pbio.3001802.s007.docx]

# S7_Note: Orthologs of further circadian clock components

We identified two *TaFKF1* transcripts that peaked at dusk and showed high similarity to *AtFKF1* in the PAS-domain phylogenetic tree (S14_Fig). Six transcripts similar to both *ZTL* and *LKP2* were not significantly rhythmic (S17_Fig), consistent with circadian regulation in *Arabidopsis* (Nelson et al., 2000; Schultz et al., 2001). Wheat transcripts orthologous to *LNK1/2* and *CHE* also accumulated rhythmically, however *TaCHE* peaked antiphase to *AtCHE,* at ~CT20 rather than CT8 (S17_Figure). Two putative wheat *LWD* transcripts were not rhythmically expressed (S17_Fig).

Nelson, D. C., Lasswell, J., Rogg, L. E., Cohen, M. A., & Bartel, B. (2000). FKF1, a Clock-Controlled Gene that Regulates the Transition to Flowering in Arabidopsis. *Cell*, *101*(3), 331–340. https://doi.org/10.1016/S0092-8674(00)80842-9

Schultz, T. F., Kiyosue, T., Yanovsky, M., Wada, M., & Kay, S. A. (2001). A Role for LKP2 in the Circadian Clock of Arabidopsis . *The Plant Cell*, *13*(12), 2659–2670. https://doi.org/10.1105/tpc.010332
