# Supplementary material for: Circadian regulation of the transcriptome in a complex polyploid crop
Source: PLoS Biol. 2022 Oct 13;20(10):e3001802. doi: 10.1371/journal.pbio.3001802 (PMC9560141; doi:10.1371/journal.pbio.3001802)
Supplement: S8 Note — (DOCX) [file pbio.3001802.s008.docx]

# S8_Note: Considerations for the differences in circadian regulation observed between *Arabidopsis* and wheat

In the main text we observe differences in circadian regulation of several transcripts encoding proteins involved in primary metabolism, and tentatively suggest that this may reflect a less pervasive influence of the circadian clock on Tre6P and starch metabolism in wheat.

An alternative hypothesis could be that regulatory control is conveyed by transcription of enzymes or transcription factors other than the *Arabidopsis* orthologs studied here.

In wheat, the role of starch is different in photosynthetic organs (source tissues) compared to grain producing organs (sink tissues), and the source:sink ratios change throughout wheat development. Therefore, regulation by the clock may change substantially between wheat tissues, organs and developmental stages. For instance, genes which are rhythmically expressed in the shoot may not be rhythmic in the root or genes which are not rhythmic in two-week old seedlings may become rhythmic during reproductive developmental stages. Further work is needed to elucidate tissue specific differences in circadian regulation in crops with pronounced source:sink relationships.

Additionally, rhythms in transcript abundance do not necessarily translate into rhythms in protein synthesis, assembly or post-translational regulation of activity. For example, post-translational phosphorylation regulates the activity of several wheat starch branching enzymes and starch synthases (Chen et al., 2016; Tetlow et al., 2004). Future studies could explore whether differences in rhythmicity at the transcriptional level are reflected in altered oscillations of key enzymes and metabolites.

Chen, G. X., Zhou, J. W., Liu, Y. L., Lu, X. B., Han, C. X., Zhang, W. Y., Xu, Y. H., & Yan, Y. M. (2016). Biosynthesis and Regulation of Wheat Amylose and Amylopectin from Proteomic and Phosphoproteomic Characterization of Granule-binding Proteins. *Scientific Reports*, *6*. https://doi.org/10.1038/srep33111

Tetlow, I. J., Wait, R., Lu, Z., Akkasaeng, R., Bowsher, C. G., Esposito, S., Kosar-Hashemi, B., Morell, M. K., & Emes, M. J. (2004). Protein phosphorylation in amyloplasts regulates starch branching enzyme activity and protein-protein interactions. *Plant Cell*, *16*(3), 694–708. https://doi.org/10.1105/tpc.017400
