# Supplementary figures and images for: Circadian regulation of the transcriptome in a complex polyploid crop
Source: PLoS Biol. 2022 Oct 13;20(10):e3001802. doi: 10.1371/journal.pbio.3001802 (PMC9560141; doi:10.1371/journal.pbio.3001802)

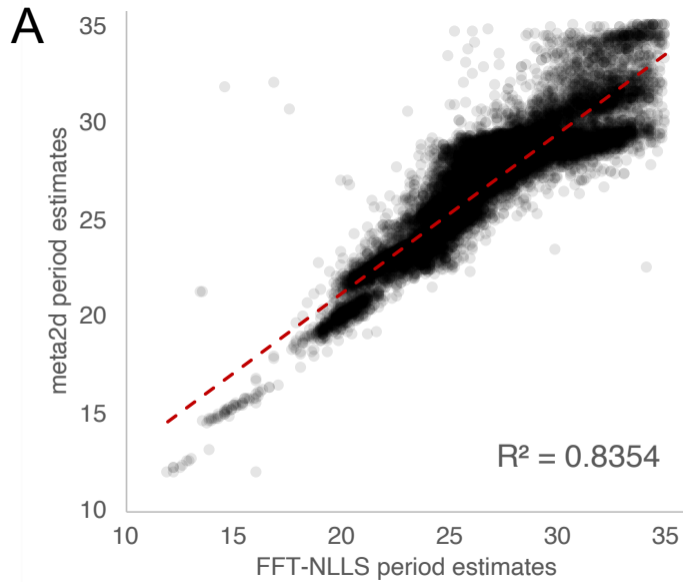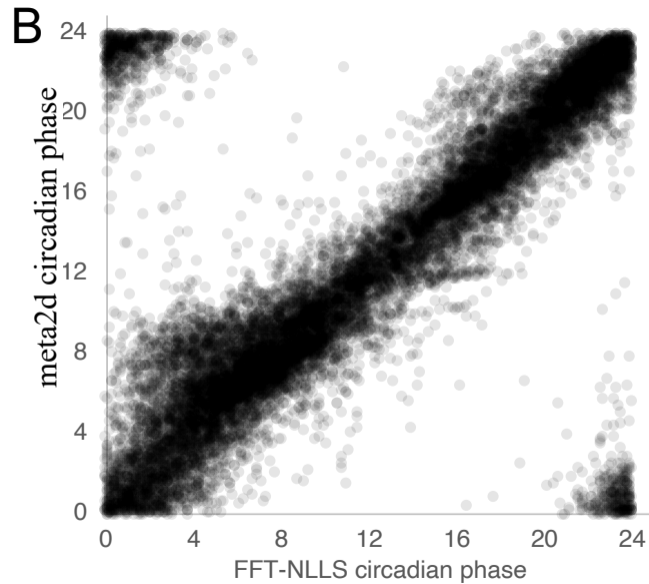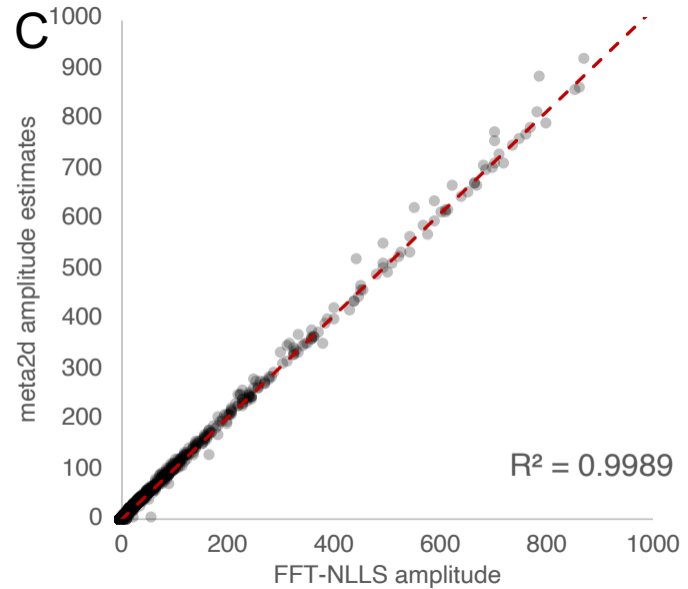

Supplement: S1 Fig — Meta2d was run in Metacycle and FFT-NLLS in Biodare2 using 24–68 h data filtered for rhythmicity B.H q-values <0.01. (Data_Fig_S1a-c in S2 Data). (PDF) [file pbio.3001802.s009.pdf]

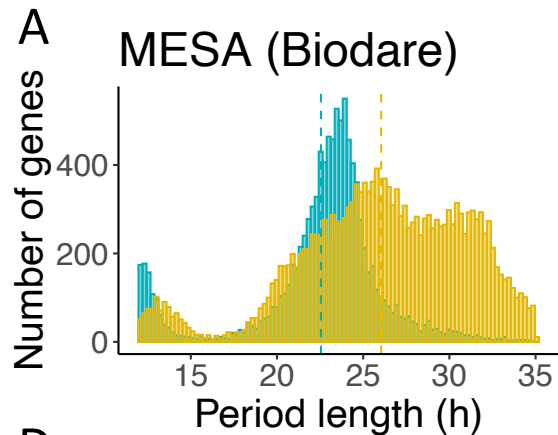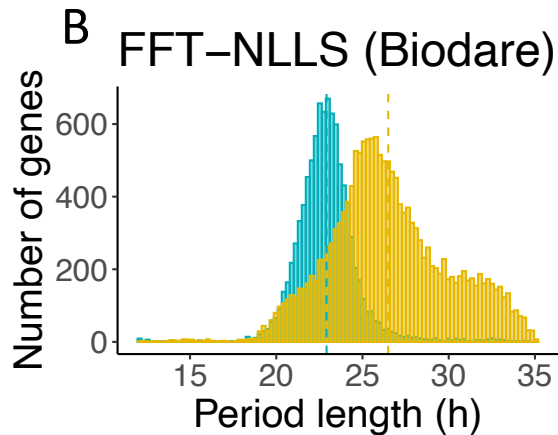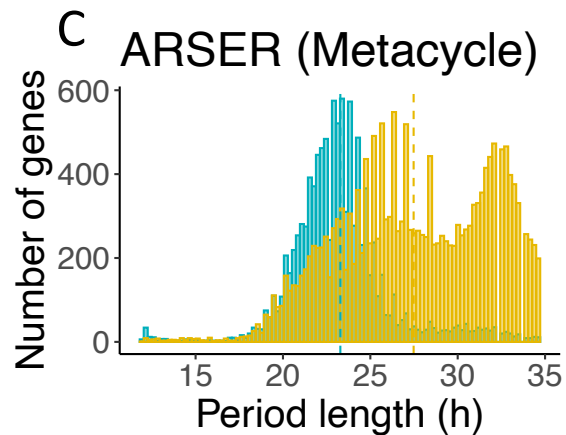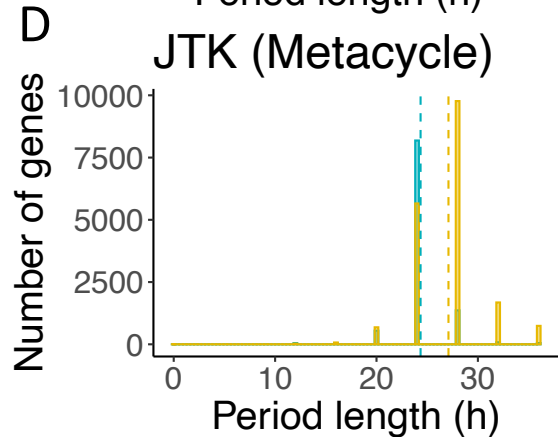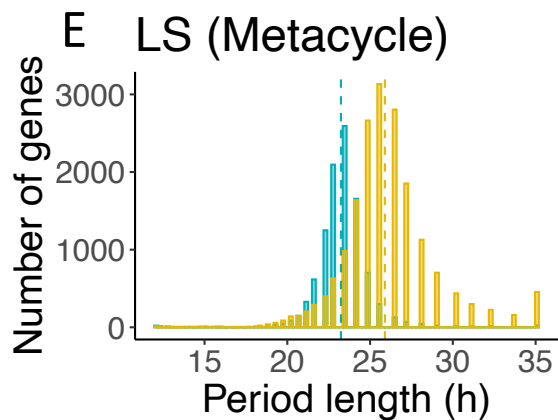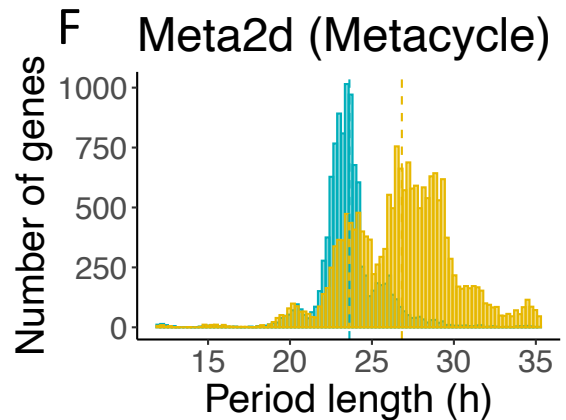

Supplement: S2 Fig — MESA and FFT-NLLS are independently run in Biodare2 (A and B), ARSER, JTK, and LS (C–E) are all run through Metacycle to produce an average period prediction meta2d (F). Data for Arabidopsis (blue) and wheat (yellow) was filtered for BH q < 0.01 on a data window of 24–68 h after transfer to constant light. (Data_Fig_S2a-f in S2 Data). (PDF) [file pbio.3001802.s010.pdf]

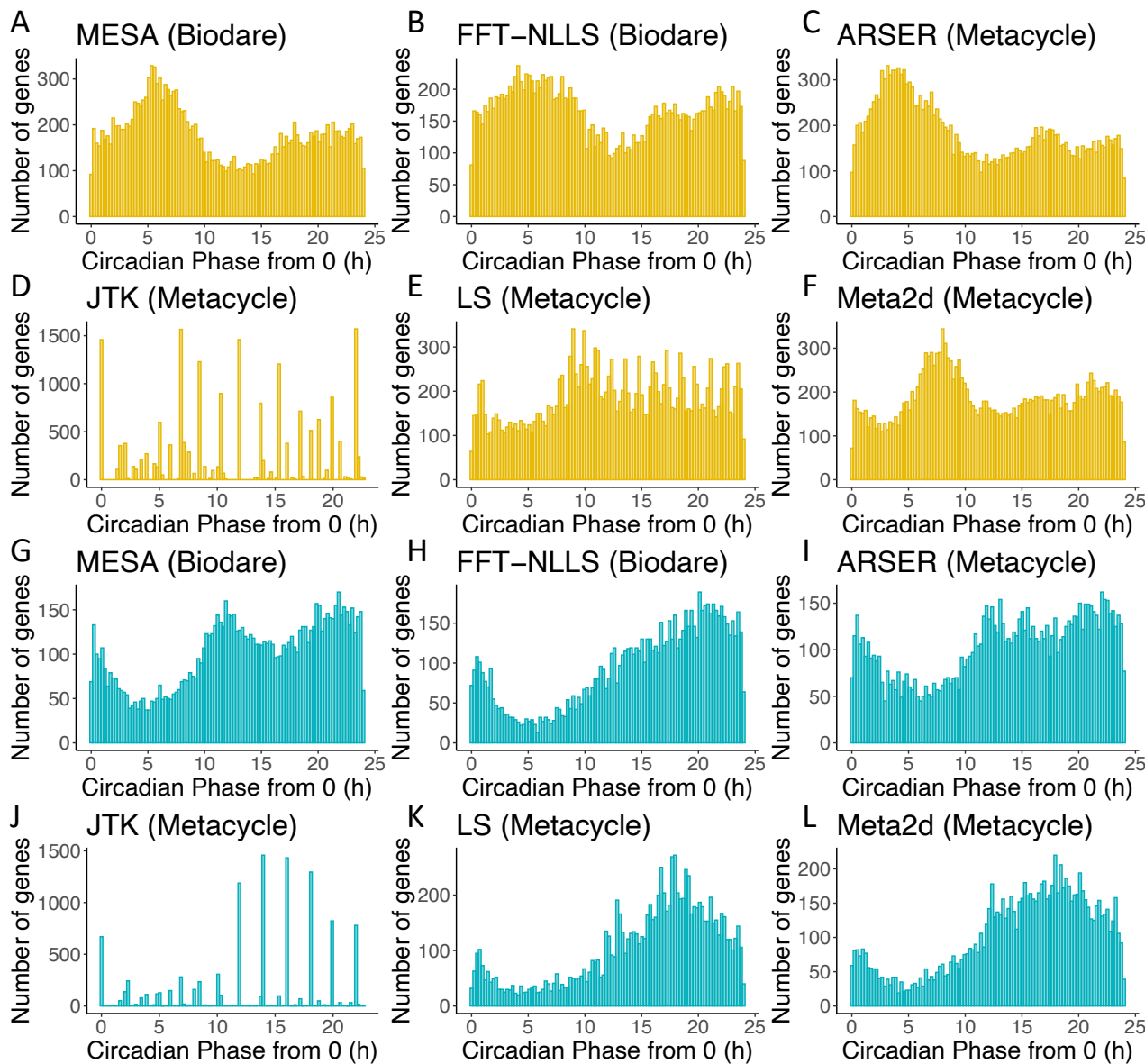

Supplement: S3 Fig — Phases were adjusted by period length so that 1 complete cycle is equal to 24 h. MESA and FFT-NLLS are independently run in Biodare2 (A and B, G and H), ARSER, JTK, and LS (C–E, I–K) are all run through Metacycle to produce an average period prediction meta2d (F and L). Data for Arabidopsis (blue) and wheat (yellow) was filtered for BH q < 0.01 on a data window of 24–68 h after dawn. (Data_Fig_S3a-l in S2 Data). (PDF) [file pbio.3001802.s011.pdf]

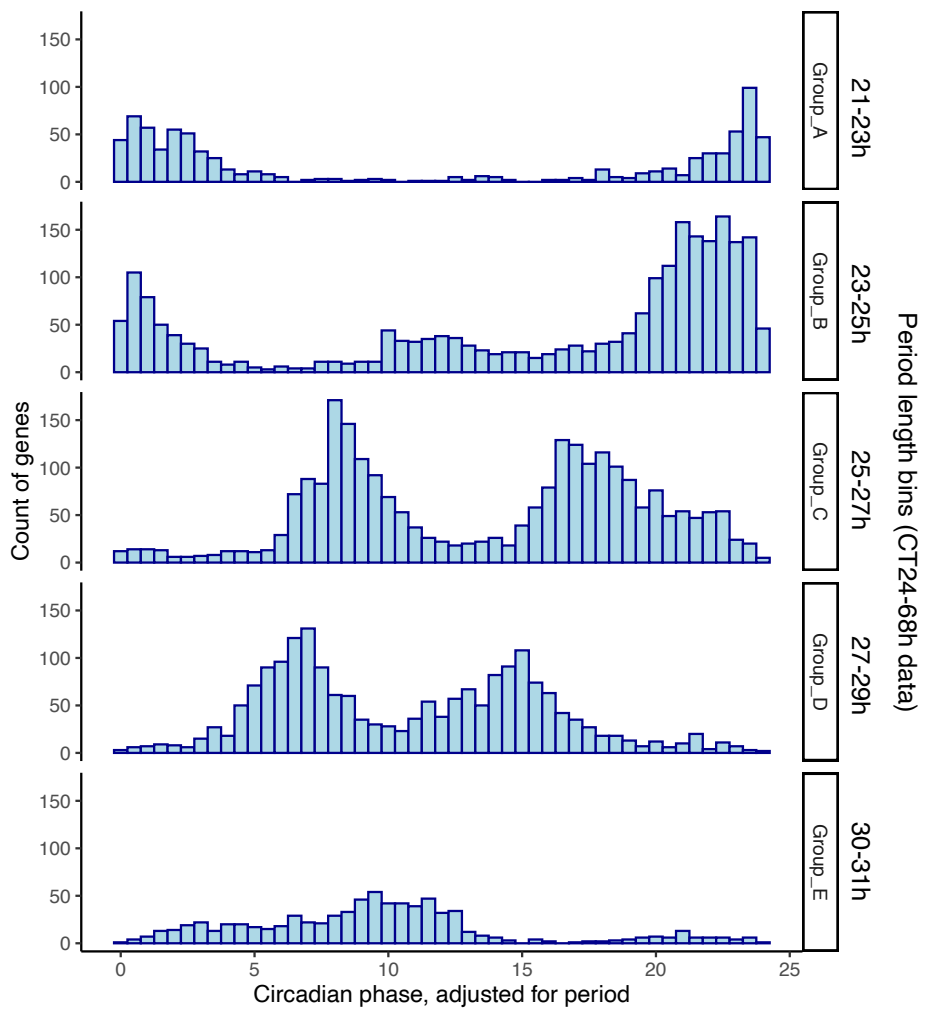

Supplement: S4 Fig — Rhythmic genes were defined as having Metacycle q-values of <0.01 over 24–68 h data relative to dawn. To improve accuracy, genes were placed into a period bin if both meta2d (Metacycle) and FFT-NLLS (Biodare2) predicted period lengths within the same 2 h window for each gene and were discarded if they fell into different bins. Meta2d phases were then recalculated relative to the meta2d period length (phase * 24/period) per gene. Histograms show that genes with shorter period lengths tended to have more dawn-peaking genes and genes with longer periods tended to have more dusk peaking genes. (Data_Fig_S4 in S2 Data). (PDF) [file pbio.3001802.s012.pdf]

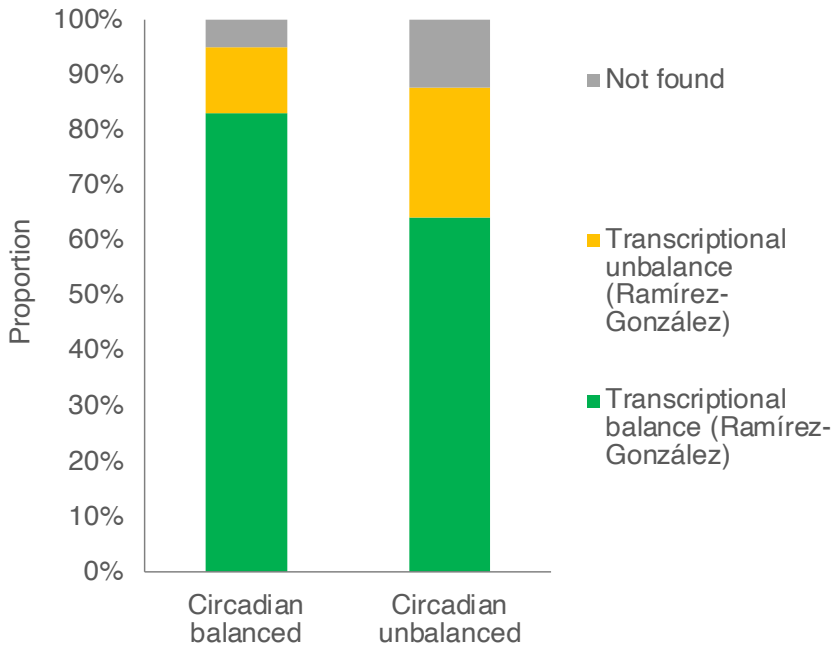

chi-squared = 689.84, df = 2,  $p$ -value < 2.2e-16

Supplement: S5 Fig — A chi-square test was used to determine whether there was a significant difference between the proportion of balanced and imbalanced triads (as defined by Ramírez-González (2020)) in each of the circadian-balanced and circadian-imbalanced triad categories. Results indicated that 83.20% of circadian-balanced triads were also categorised as balanced in the Ramírez-González data, whereas 64.15% of circadian-imbalanced triads were categorised as balanced in the Ramírez-González data. This difference was significant, χ2(2) = 689.8, p < 0.00001. These data also show that a large proportion of circadian triads labelled as balanced in the Ramírez-González dataset would be expected to have imbalanced patterns of expression if measured over several time points under circadian conditions, highlighting the importance of considering temporal dynamics in transcriptomic studies. Ramírez-González data is from Chinese spring leaves (excluding flag-leaf) under non-stressed conditions to provide as close a match to our data as possible. The diurnal time of collection is unknown. (Data_Fig_S5 in S2 Data). (PDF) [file pbio.3001802.s013.pdf]

Sub-genome

A —  
B —  
D —

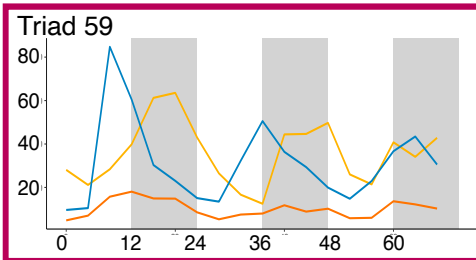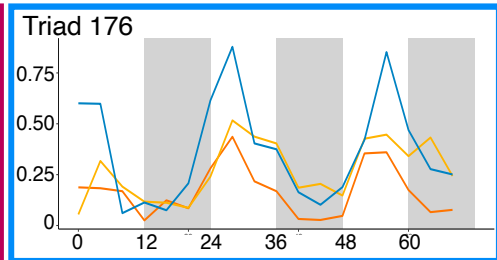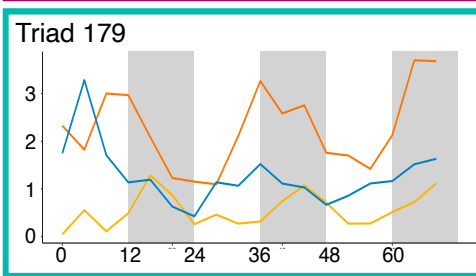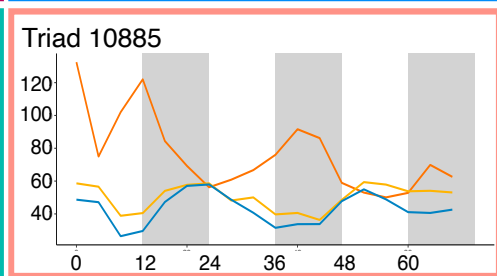

Triads

59  
176  
179  
10885

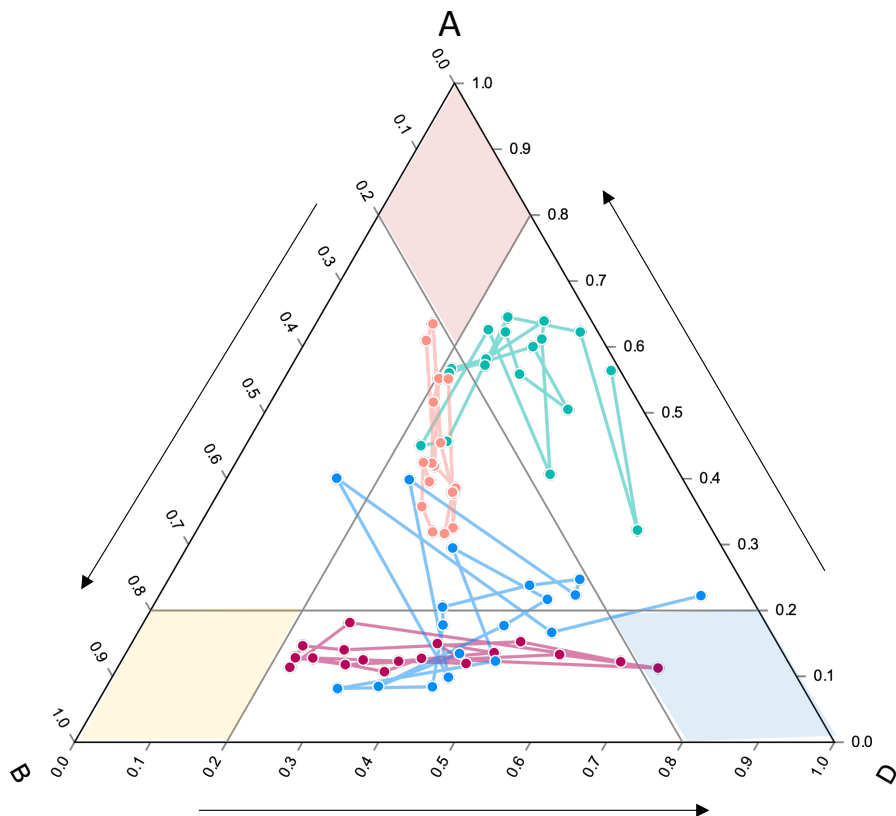

Supplement: S6 Fig — Each point represent expression normalised to 1 within each triad. Lines connect the dynamic changes in balance across the time course. Triad 59: A suppressed across all time points, but dominance of B and D vary across the time course. All homoeologs are classified as rhythmic (q < 0.05) but have imbalanced phases. Triad 176: Mostly appears as balanced across the time course but occasionally looks as though A is suppressed. All homeologs in this triad are rhythmic (q < 0.05) but have imbalanced relative amplitudes and periods. Triad 179: B suppressed over most time points, but when A homoeolog has a trough of expression appears as balanced. This triad has imbalanced phases and periods but all 3 homeologs are rhythmic (q < 0.05). Triad 10885: In this case, the A homoeolog is antiphase to the other homoeologs, and so in dawn time points the triad appears balanced, but in dusk time points there is A dominance. Ternary plots were created using https://www.ternaryplot.com/. (Data_Fig_S6 in S2 Data). (PDF) [file pbio.3001802.s014.pdf]

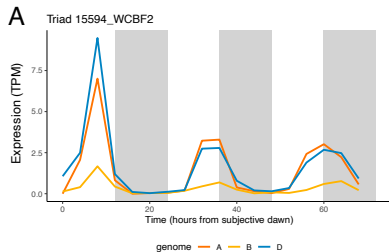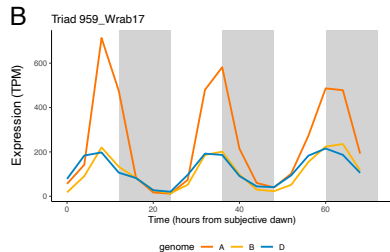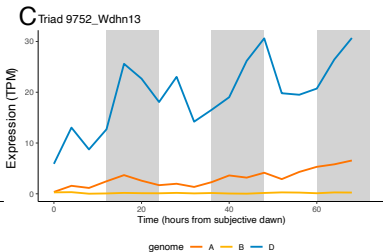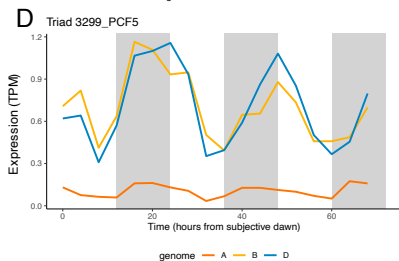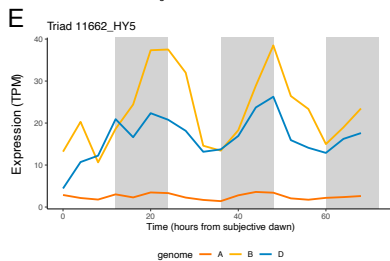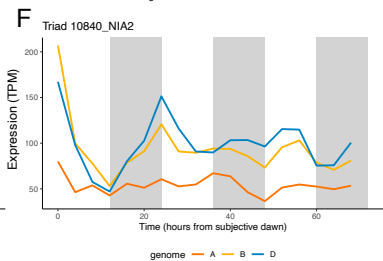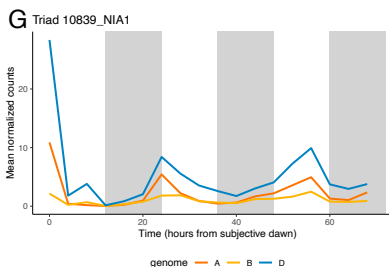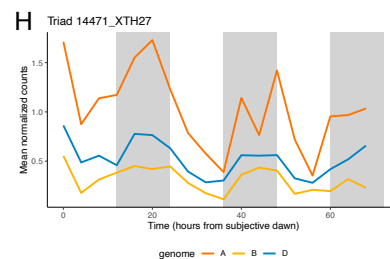

Supplement: S7 Fig — Wheat WCBF2 (A) putatively regulates WDHN17 (B) and WRAB13 (C), TaPCF5 (D) putatively regulates wheat orthologs of HY5 (E), NIA2 (F), NIA1 (G), and XTH27 (H). (Data_Fig_S7 in S2 Data). (PDF) [file pbio.3001802.s015.pdf]

# W1

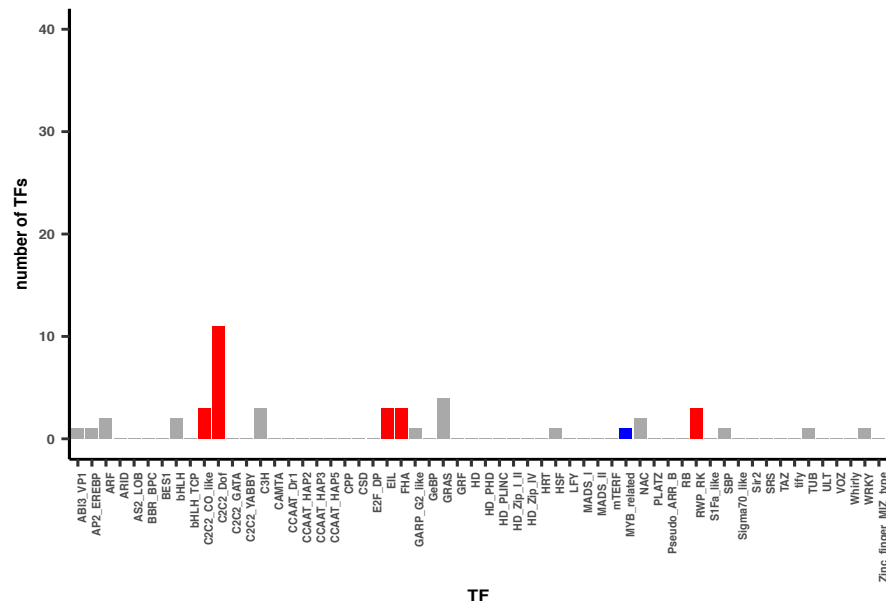

# W2

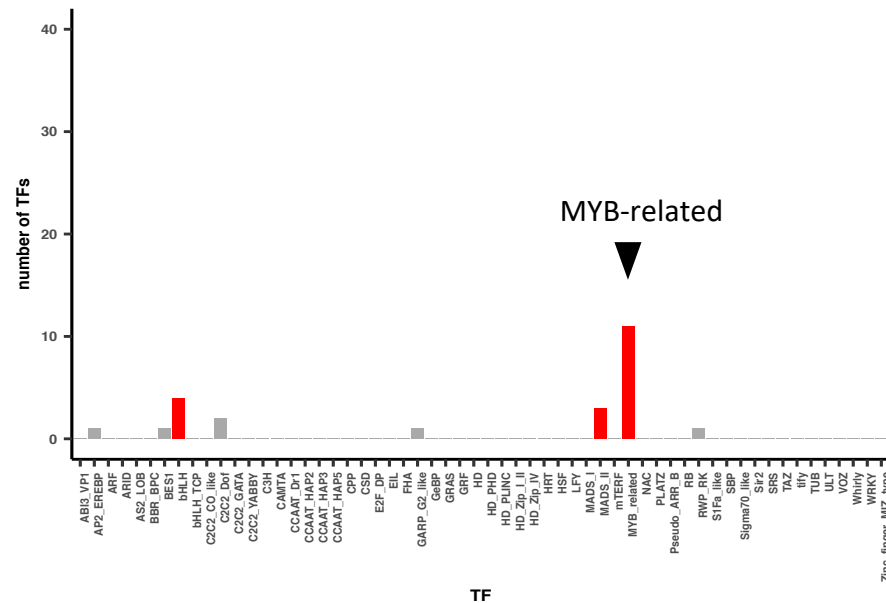

# W3

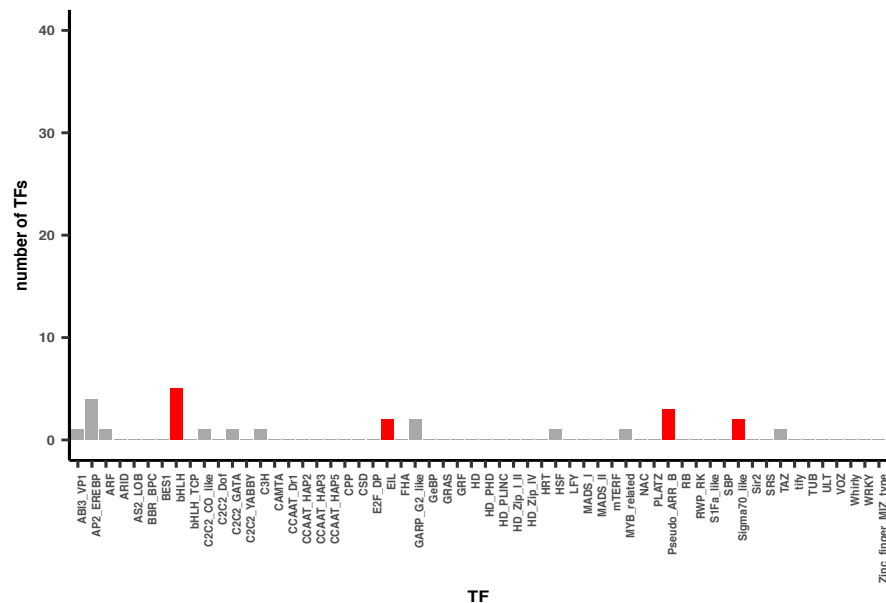

# W4

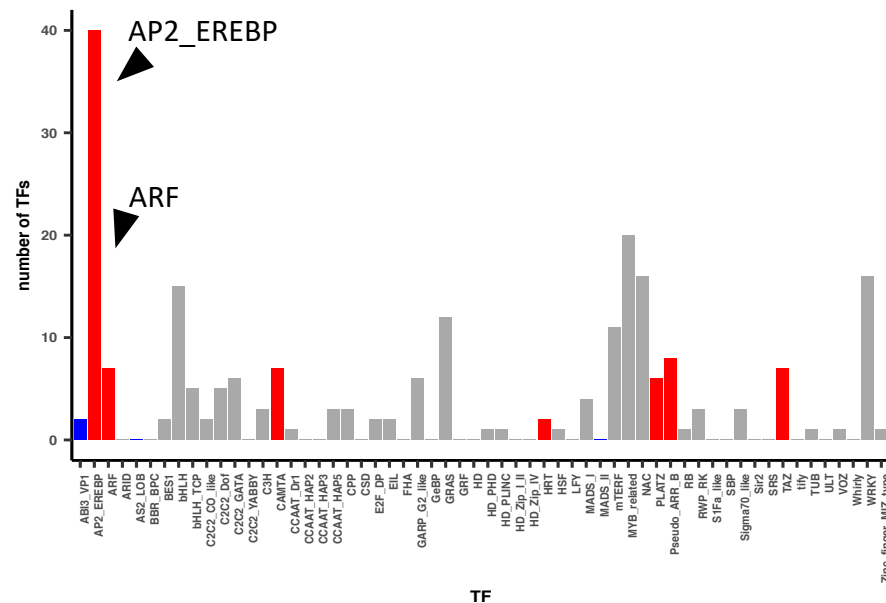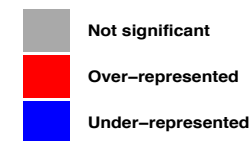

W5

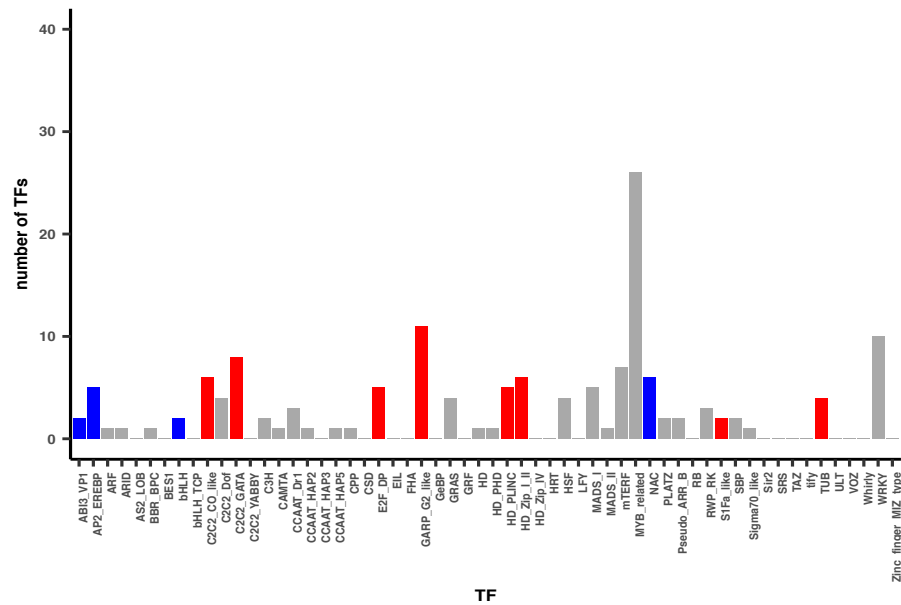

W6

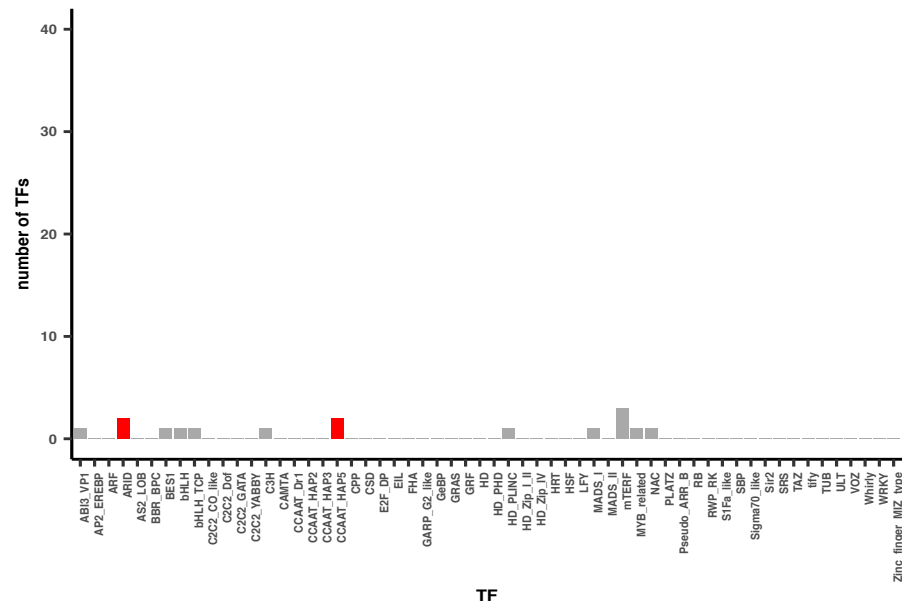

W7

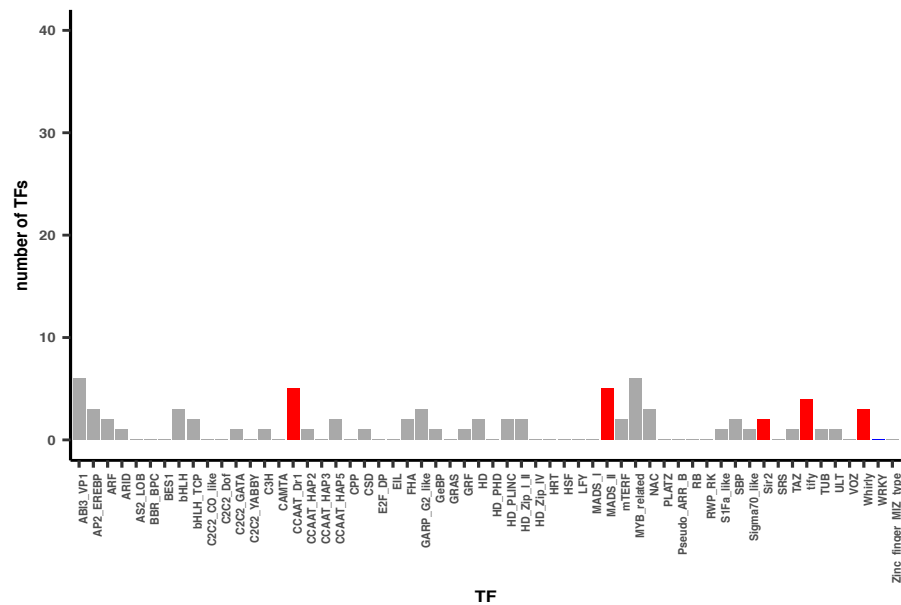

W8

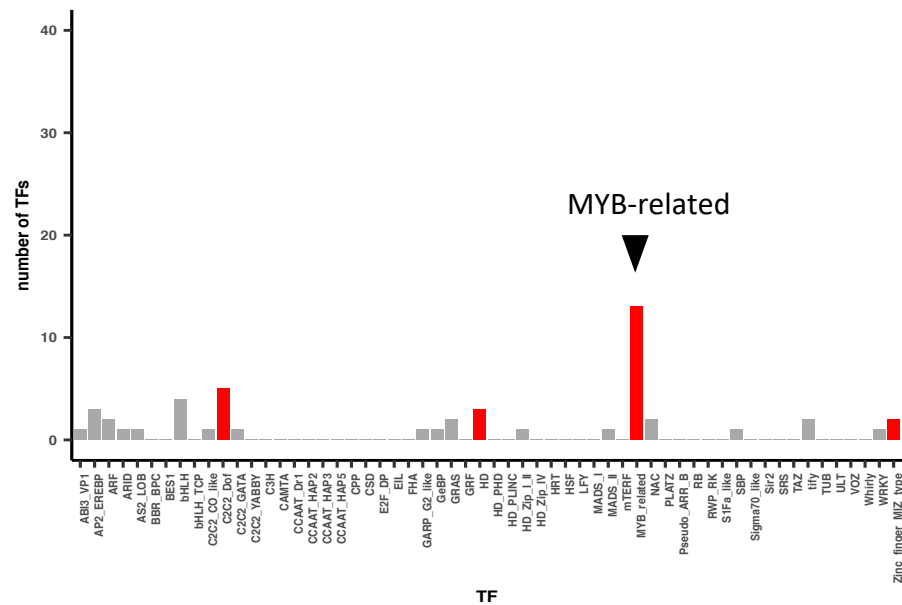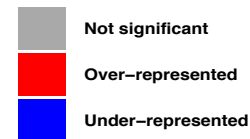

W9

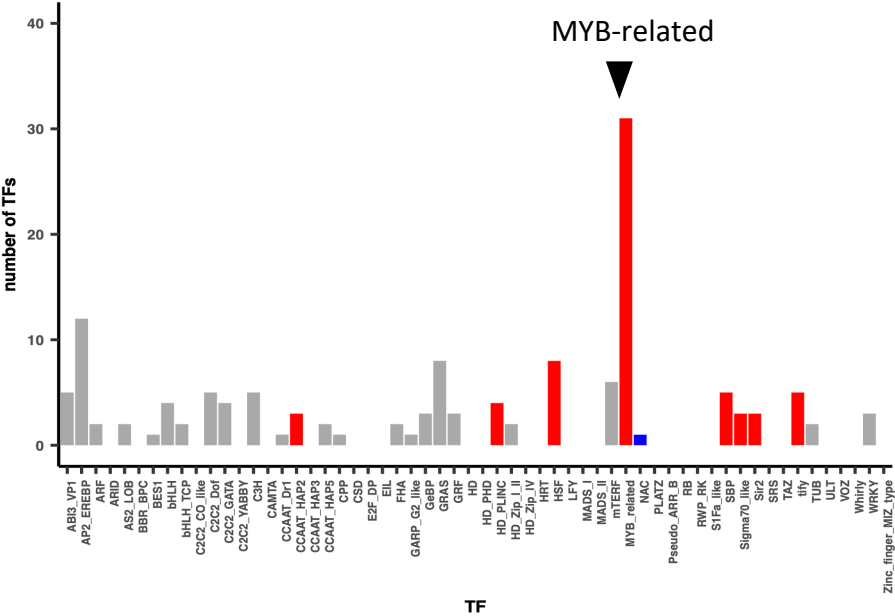

Supplement: S10 Fig — Barcharts showing number of genes (y axis) belonging to each TF superfamily (x axis) within wheat co-expression modules W1-W9. Coloured bars denote those superfamilies significantly over-enriched (red) or under-enriched (blue) compared to the total number of TF superfamilies present in the 16,327 genes submitted to WGCNA (Fisher’s exact test, p < = 0.05). Families not significantly over- or under-enriched are coloured grey. (Data_Fig_S10 in S2 Data). (PDF) [file pbio.3001802.s018.pdf]

W1

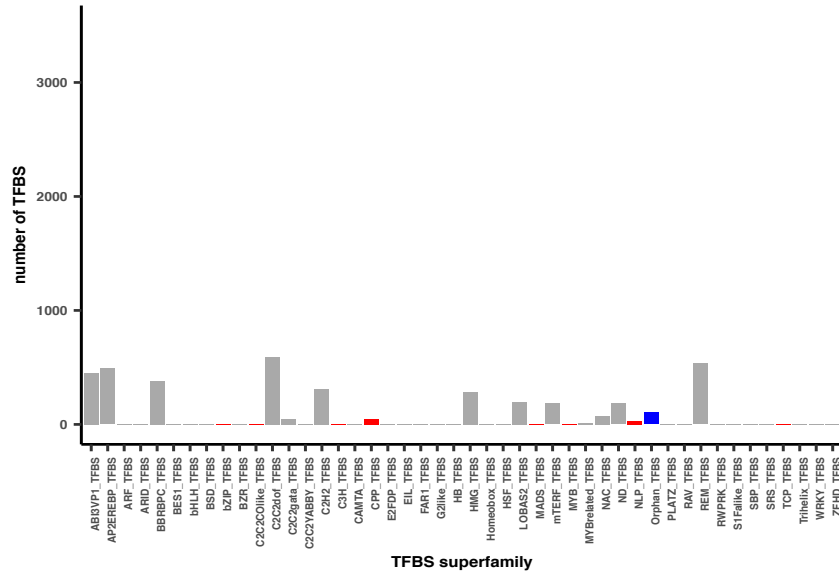

W2

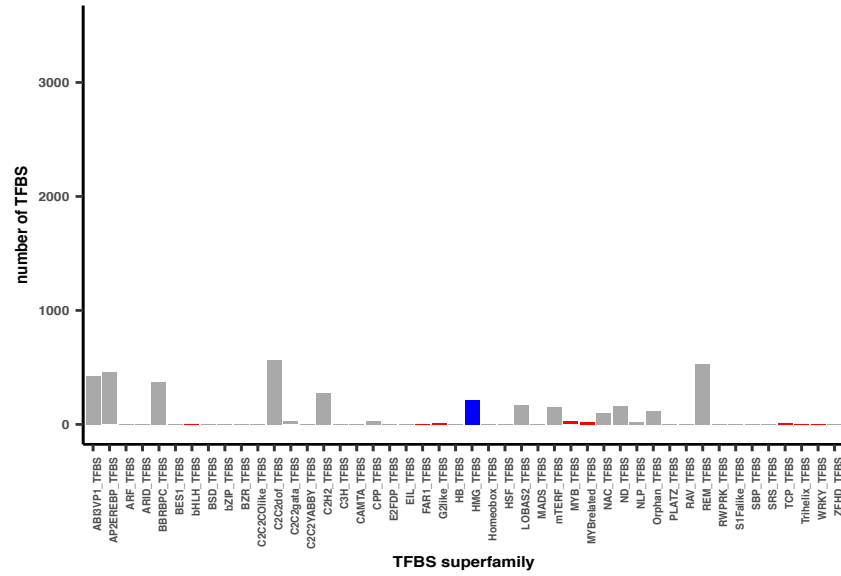

W3

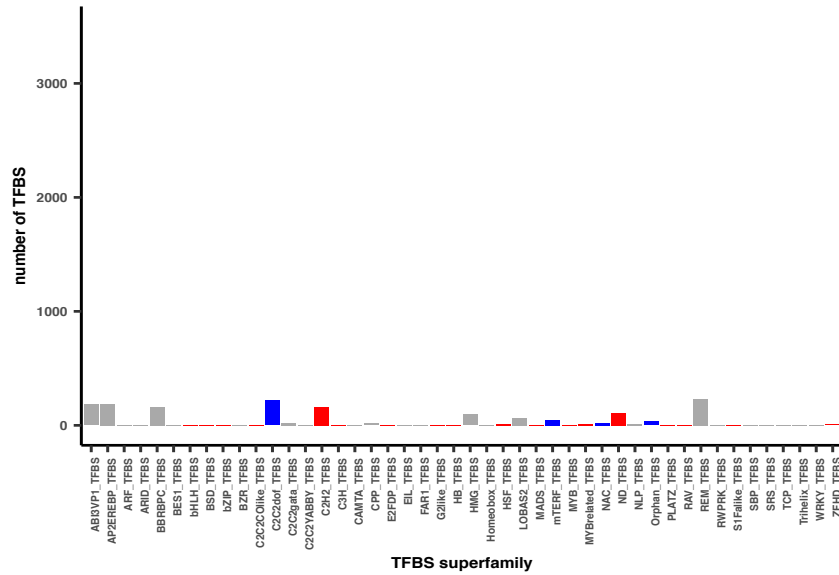

W4

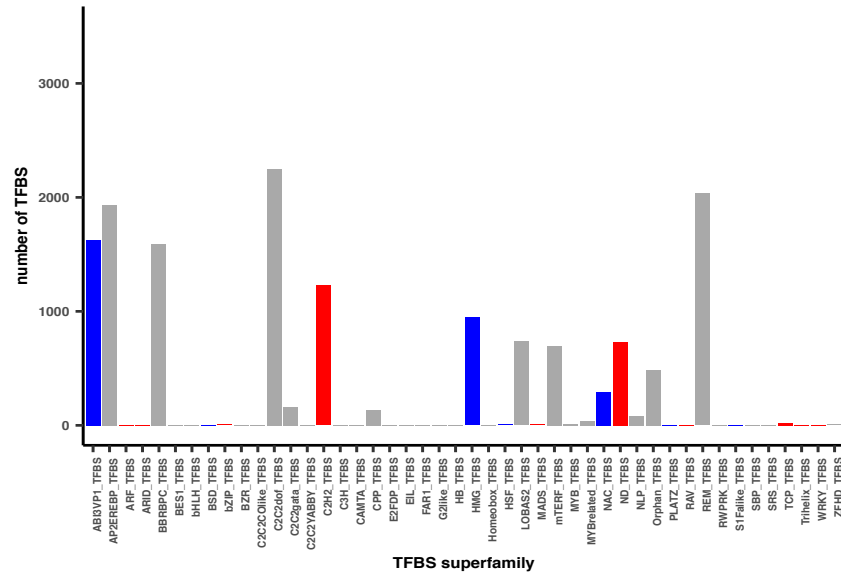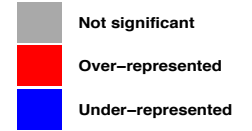

W5

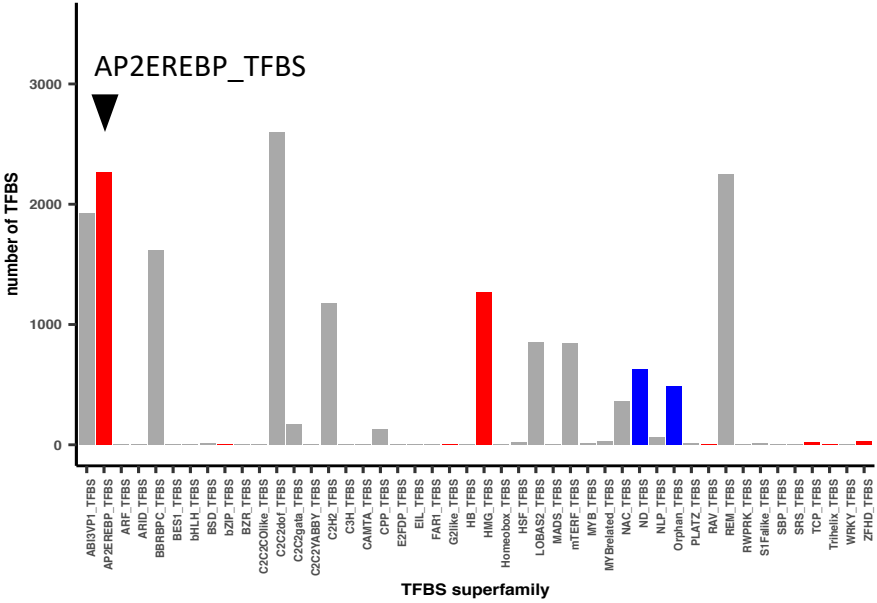

W6

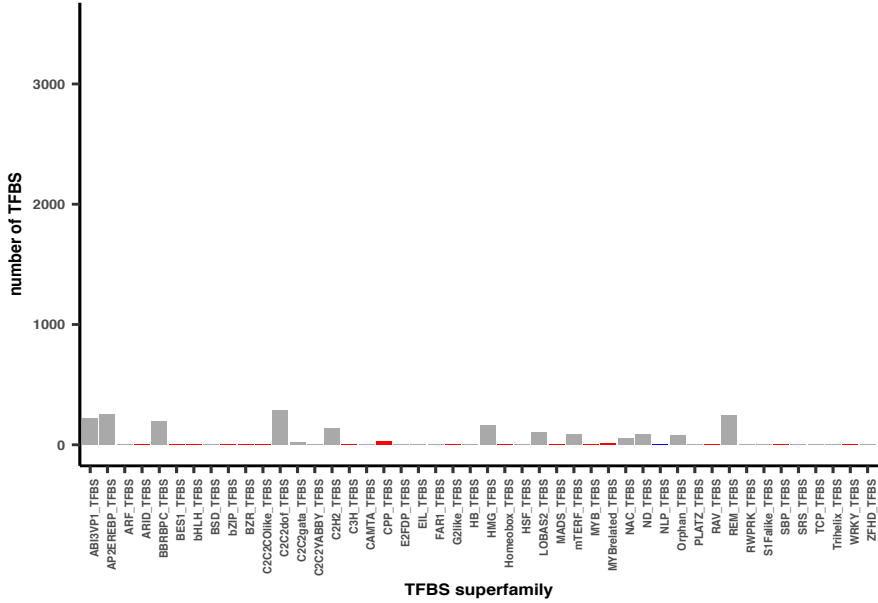

W7

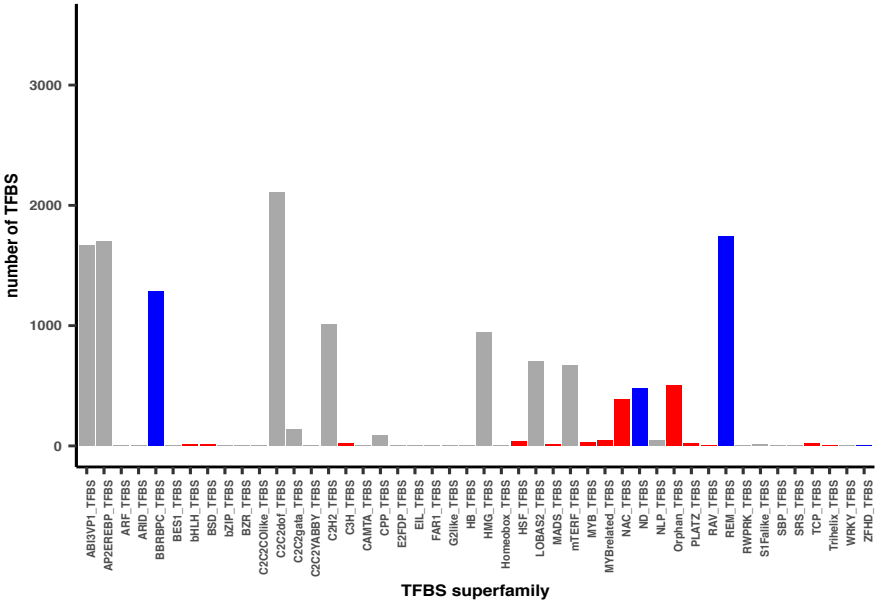

W8

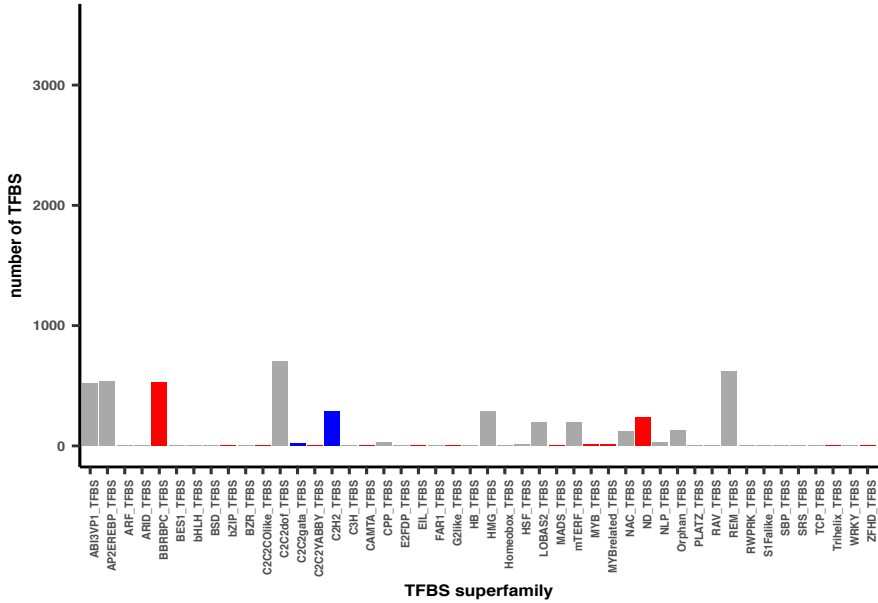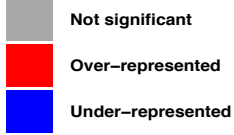

W9

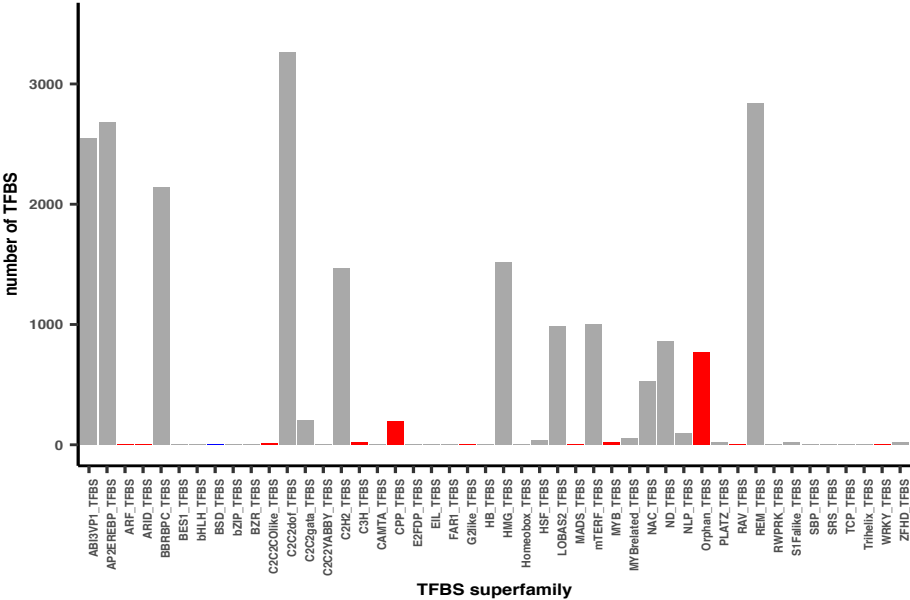

Supplement: S11 Fig — Bar charts showing number of nonredundant TFBS motifs (y axis) belonging to each TFBS superfamily (x axis) within wheat co-expression modules W1-W9. Coloured bars denote those superfamilies significantly over-enriched (red) or under-enriched (blue) compared to the total number of nonredundant TFBS motif superfamilies present in the 16,327 genes submitted to WGCNA (Fisher’s exact test, p < = 0.05). Families not significantly over- or under-enriched are coloured grey. (Data_Fig_S11 in S2 Data). (PDF) [file pbio.3001802.s019.pdf]

Tree 1: MYB (LHY-like)

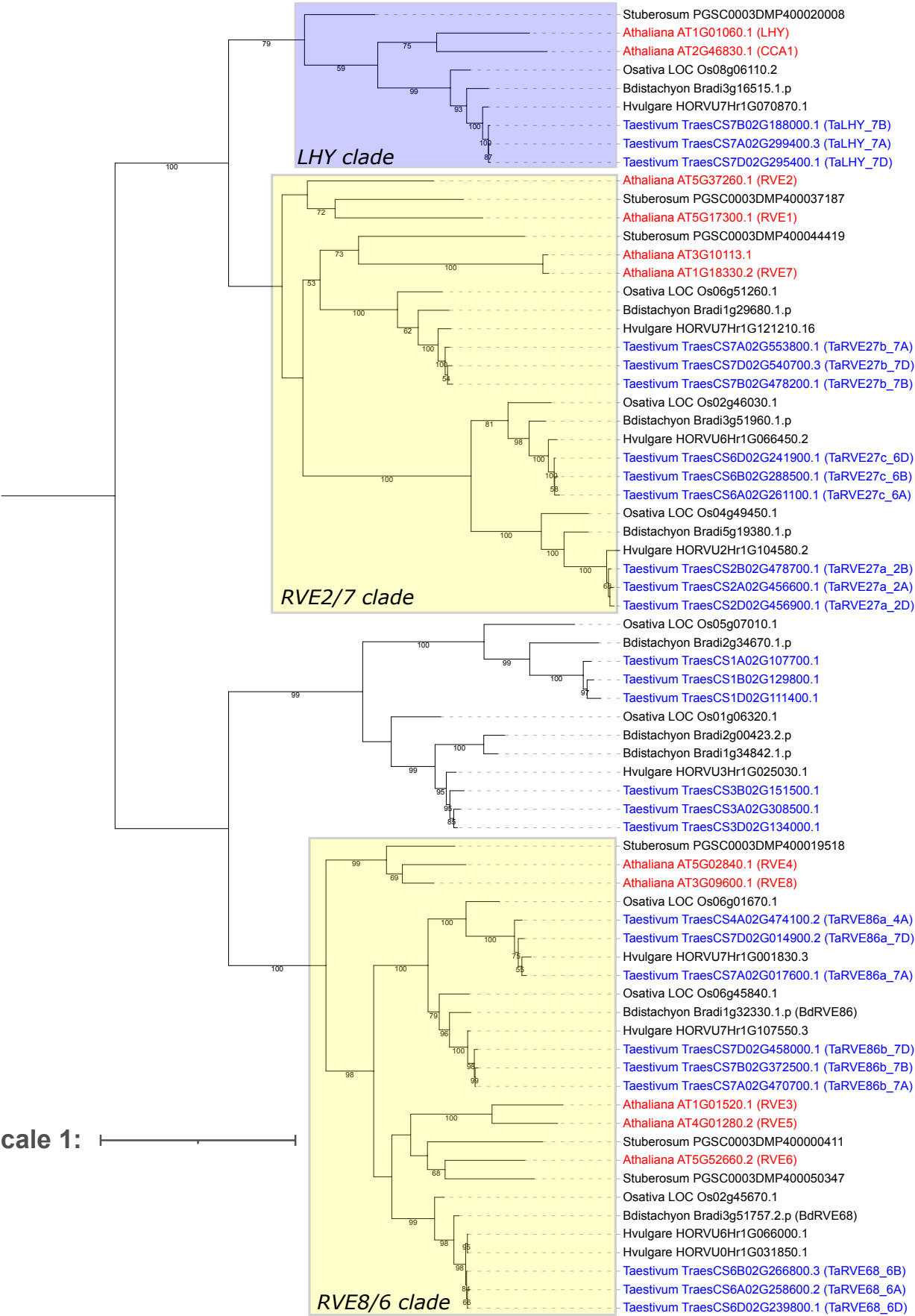

Supplement: S12 Fig — The species identifiers for each species have been abbreviated as follows: Taestivum: Triticum aestivum (hexaploid wheat), Bdistachyon: Brachypodium distachyon, Hvulgare: Hordeum vulgare (barley), Osativa: Oryza sativa (rice), Stuberosum: Solanum tuberosum (potato), Athaliana: Arabidopsis thaliana. Wheat genes have been highlighted in blue and Arabidopsis genes in red for clarity. Bootstrap values are calculated using RAxML, with values over 50 shown on branches. (PDF) [file pbio.3001802.s020.pdf]

## Tree 2: TOC1-like

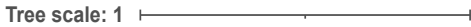

Supplement: S13 Fig — The species identifiers for each species have been abbreviated as follows: Taestivum: Triticum aestivum (hexaploid wheat), Bdistachyon: Brachypodium distachyon, Hvulgare: Hordeum vulgare (barley), Osativa: Oryza sativa (rice), Stuberosum: Solanum tuberosum (potato), Athaliana: Arabidopsis thaliana. Wheat genes have been highlighted in blue and Arabidopsis genes in red for clarity. Bootstrap values are calculated using RAxML, with values over 50 shown on branches. (PDF) [file pbio.3001802.s021.pdf]

# Tree 3: PAS/LOV (ZTL-like)

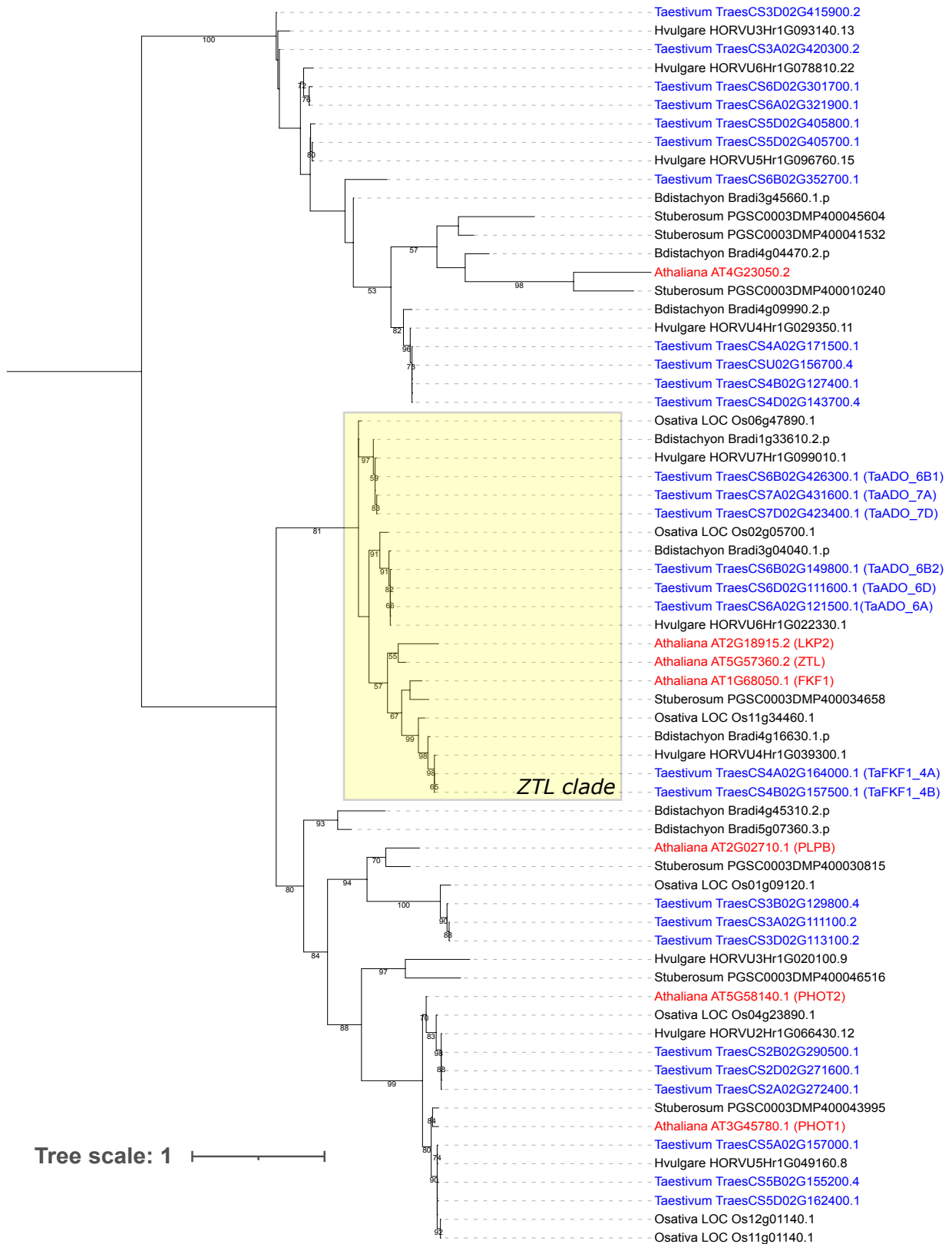

Supplement: S14 Fig — The species identifiers for each species have been abbreviated as follows: Taestivum: Triticum aestivum (hexaploid wheat), Bdistachyon: Brachypodium distachyon, Hvulgare: Hordeum vulgare (barley), Osativa: Oryza sativa (rice), Stuberosum: Solanum tuberosum (potato), Athaliana: Arabidopsis thaliana. Wheat genes have been highlighted in blue and Arabidopsis genes in red for clarity. Bootstrap values are calculated using RAxML, with values over 50 shown on branches. (PDF) [file pbio.3001802.s022.pdf]

## Tree 4: MYB (LUX-like)

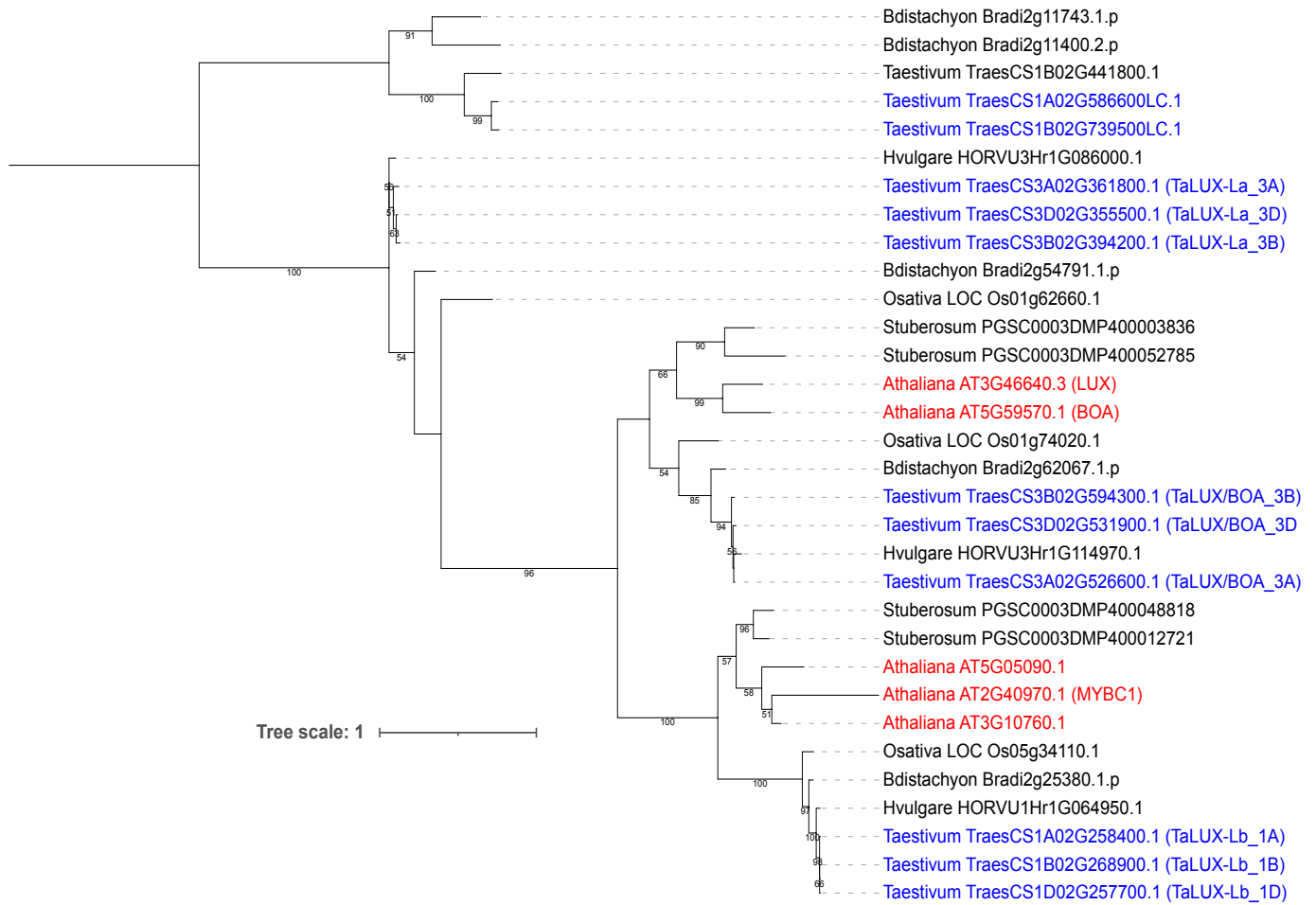

Supplement: S15 Fig — The species identifiers for each species have been abbreviated as follows: Taestivum: Triticum aestivum (hexaploid wheat), Bdistachyon: Brachypodium distachyon, Hvulgare: Hordeum vulgare (barley), Osativa: Oryza sativa (rice), Stuberosum: Solanum tuberosum (potato), Athaliana: Arabidopsis thaliana. Wheat genes have been highlighted in blue and Arabidopsis genes in red for clarity. Bootstrap values are calculated using RAxML, with values over 50 shown on branches. (PDF) [file pbio.3001802.s023.pdf]

## Tree 5: ELF4-like

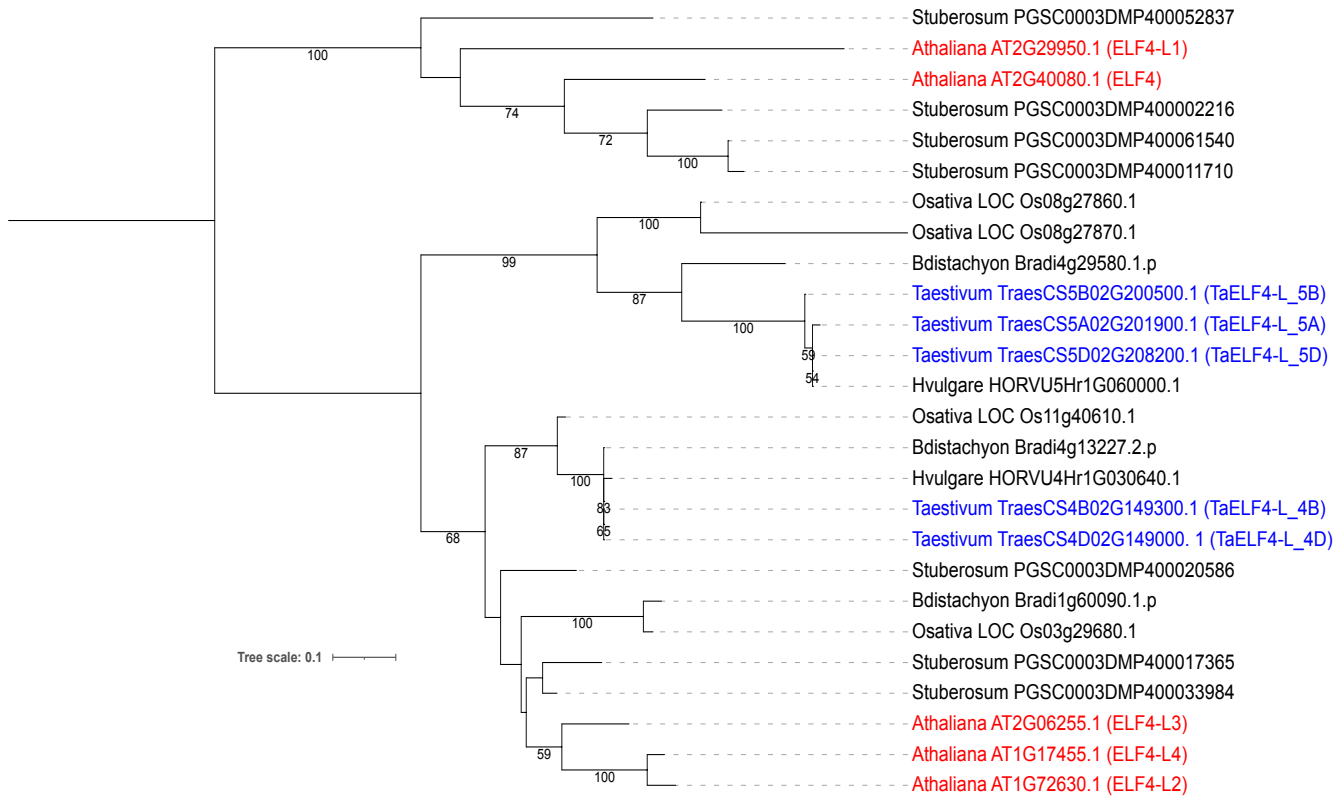

Supplement: S16 Fig — The species identifiers for each species have been abbreviated as follows: Taestivum: Triticum aestivum (hexaploid wheat), Bdistachyon: Brachypodium distachyon, Hvulgare: Hordeum vulgare (barley), Osativa: Oryza sativa (rice), Stuberosum: Solanum tuberosum (potato), Athaliana: Arabidopsis thaliana. Wheat genes have been highlighted in blue and Arabidopsis genes in red for clarity. Bootstrap values are calculated using RAxML, with values over 50 shown on branches. (PDF) [file pbio.3001802.s024.pdf]

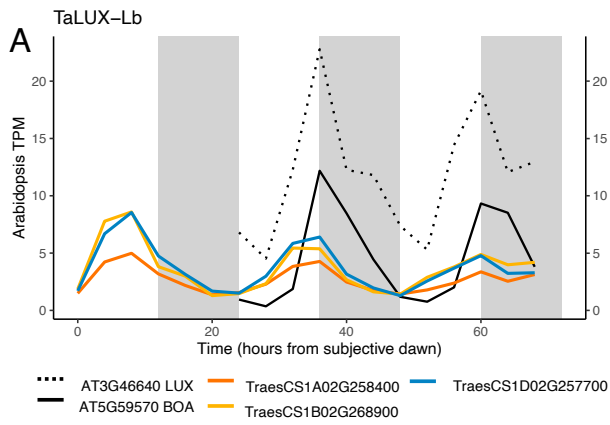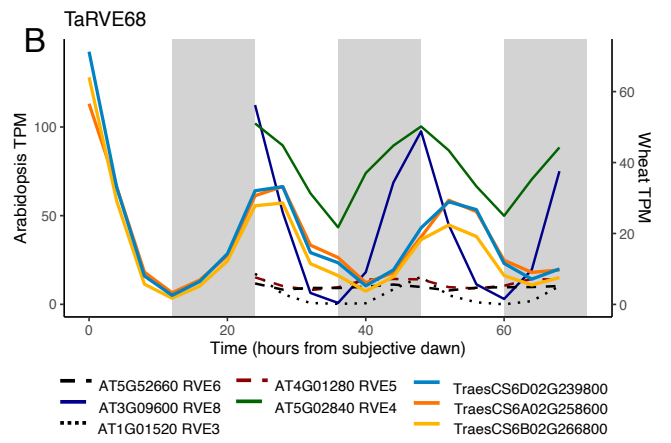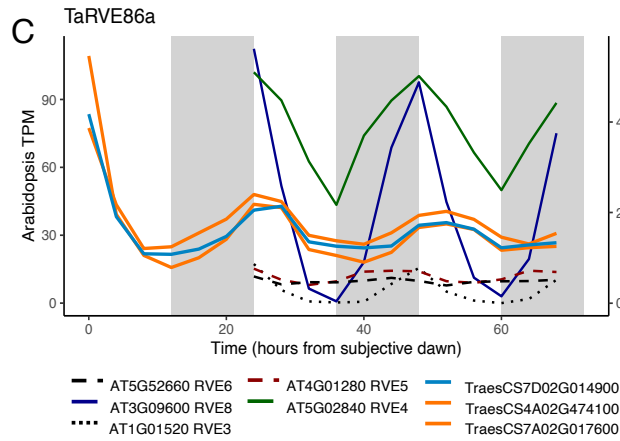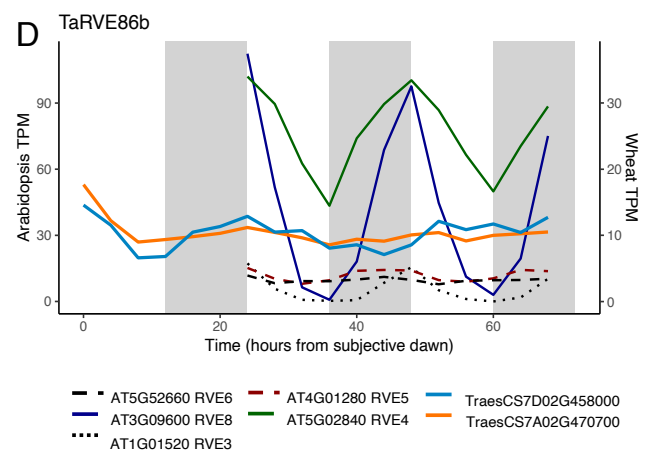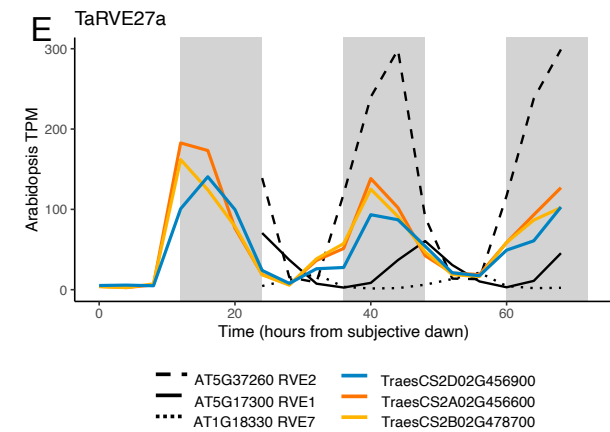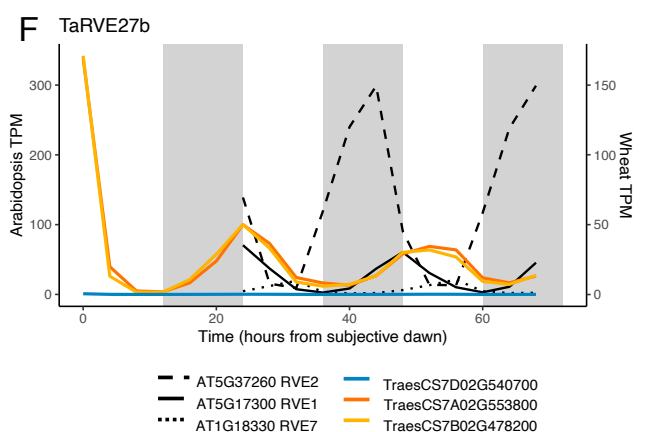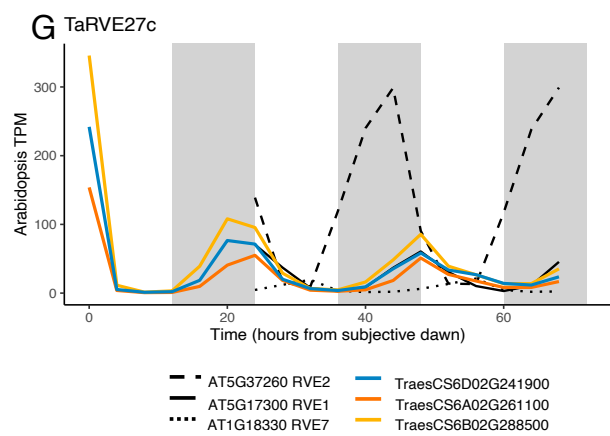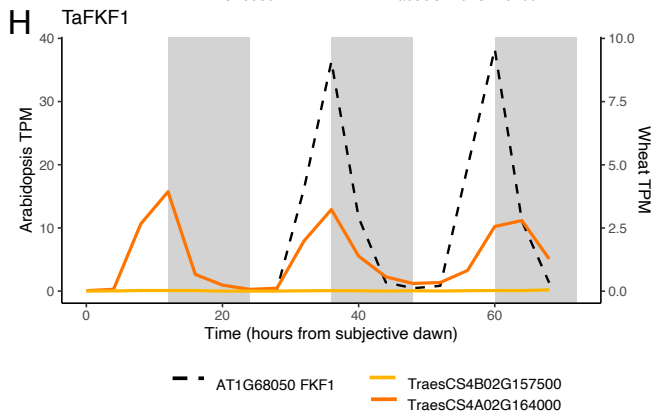

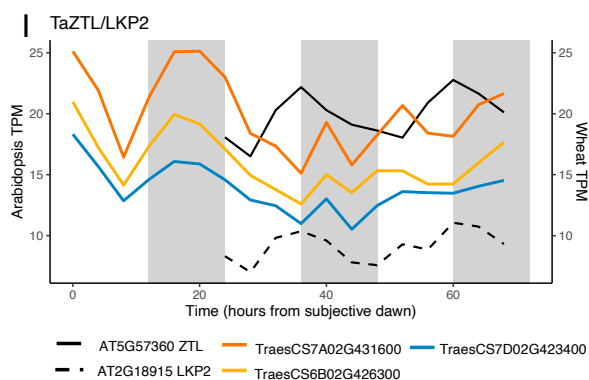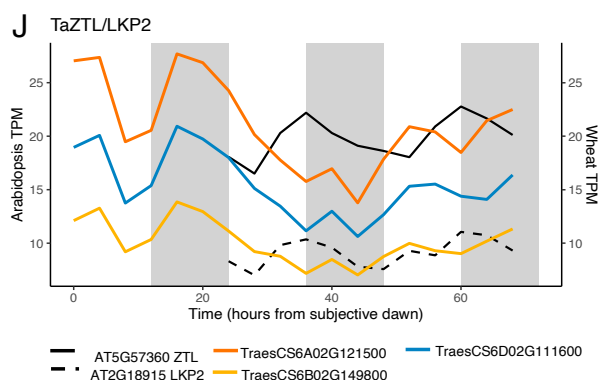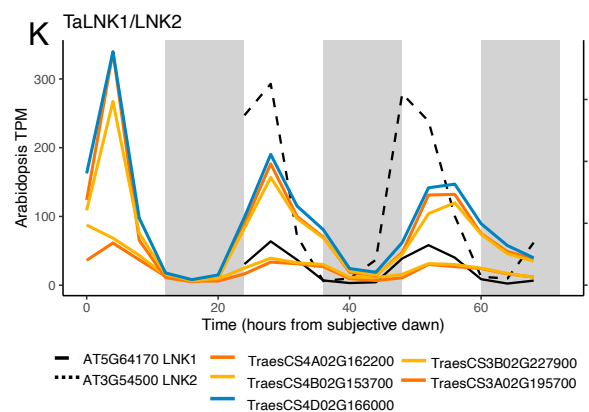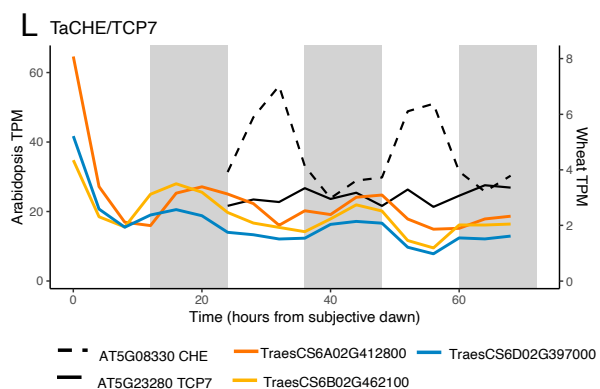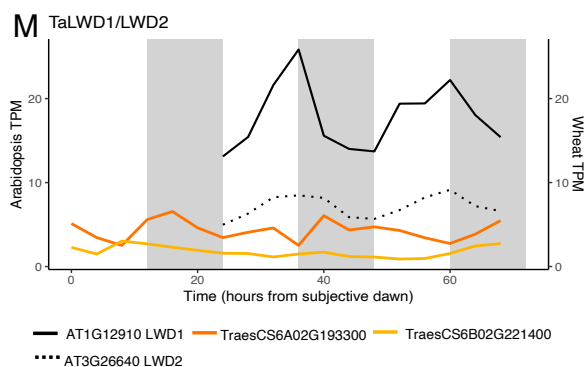

Supplement: S17 Fig — Supplementary to Fig 3 in the main text. Wheat circadian clock genes were identified through alignment of phylogenetic protein family trees or BLASTP to known clock gene homologs. Wheat homoeologs are coloured according to their identity to either the A genome (orange), B genome (yellow), or D genome (blue), and grey and white blocks indicate subjective dark and light time periods under constant conditions. Data represent the mean of 3 biological replicates and transcript expression is collapsed to gene level. (Data_Fig_S17 in S2 Data). (PDF) [file pbio.3001802.s025.pdf]

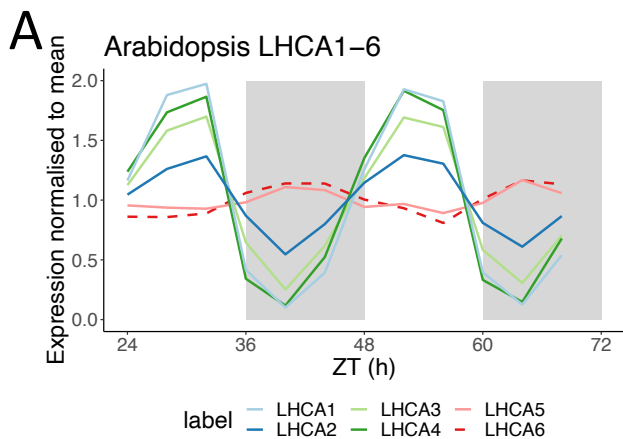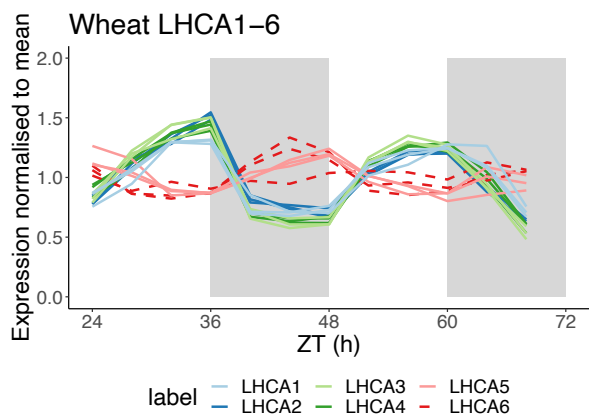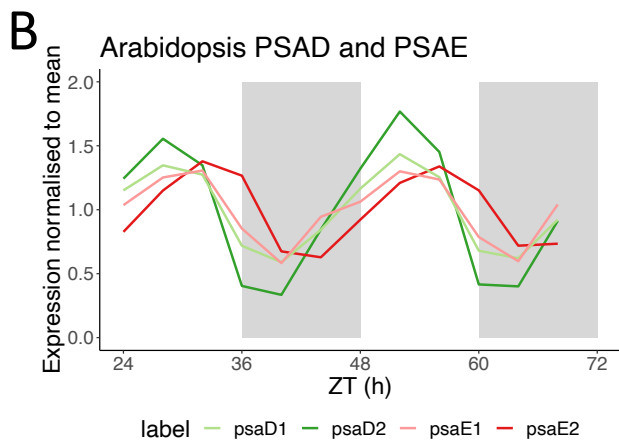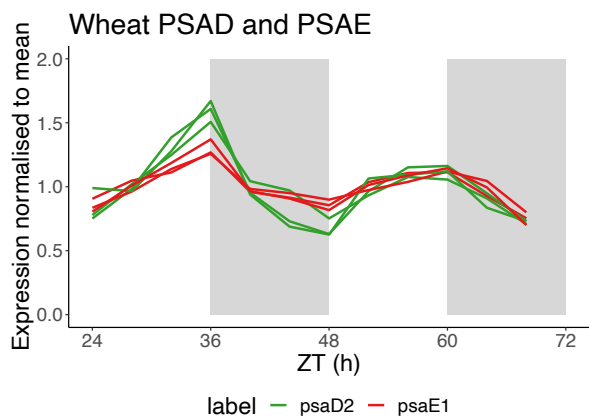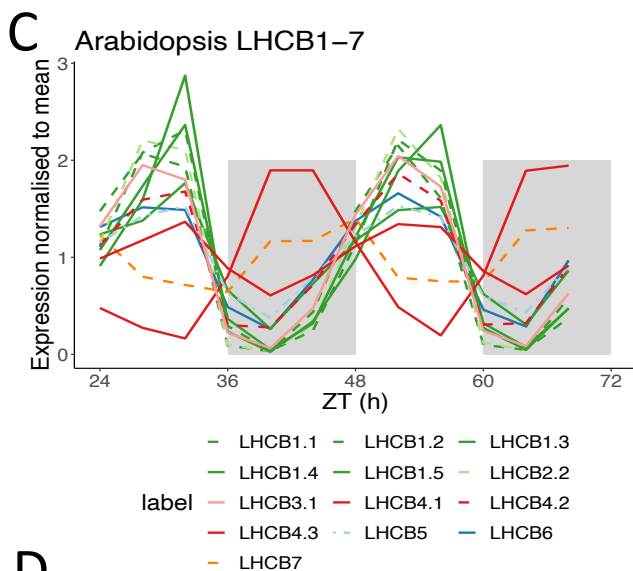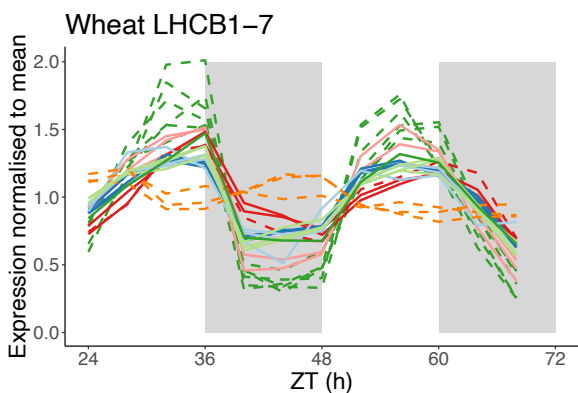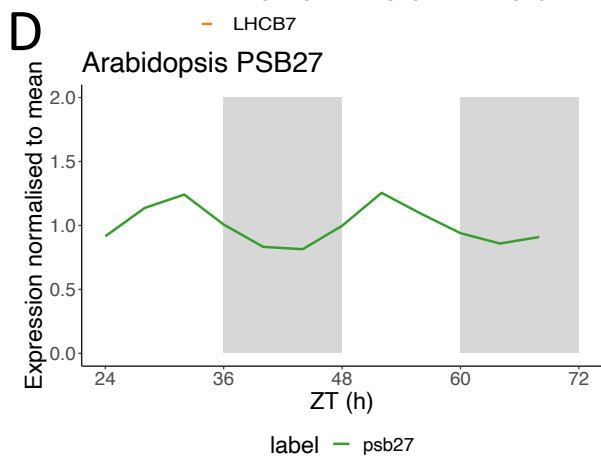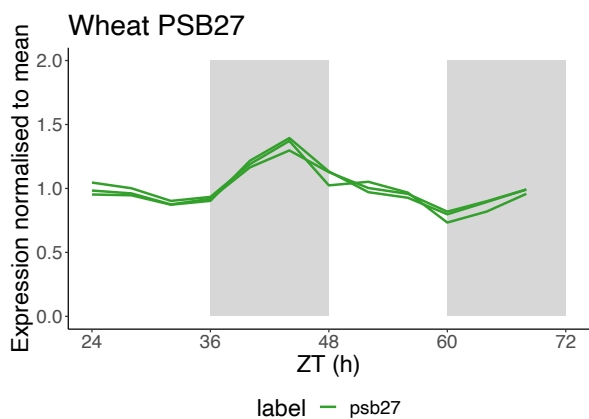

Supplement: S18 Fig — Data is mean normalised TPM. Shaded white and grey backgrounds indicate perceived day and night periods, respectively. Gene IDs for all genes plotted can be seen in S10 Table. (Data_Fig_S18-20 in S2 Data). (PDF) [file pbio.3001802.s026.pdf]

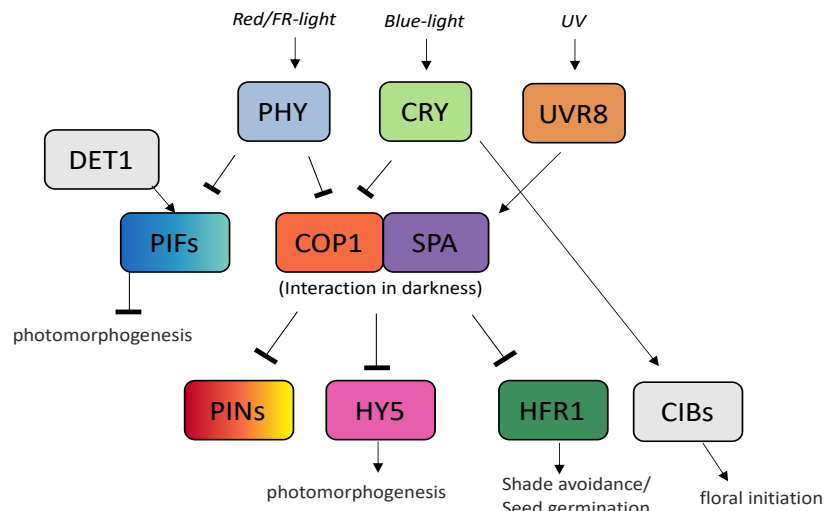

**A**

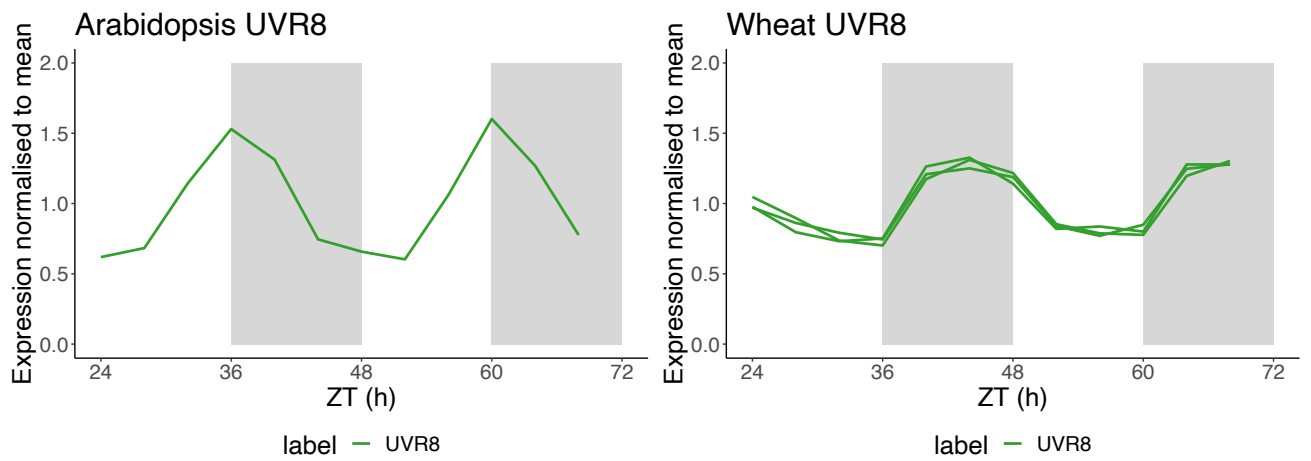

**B**

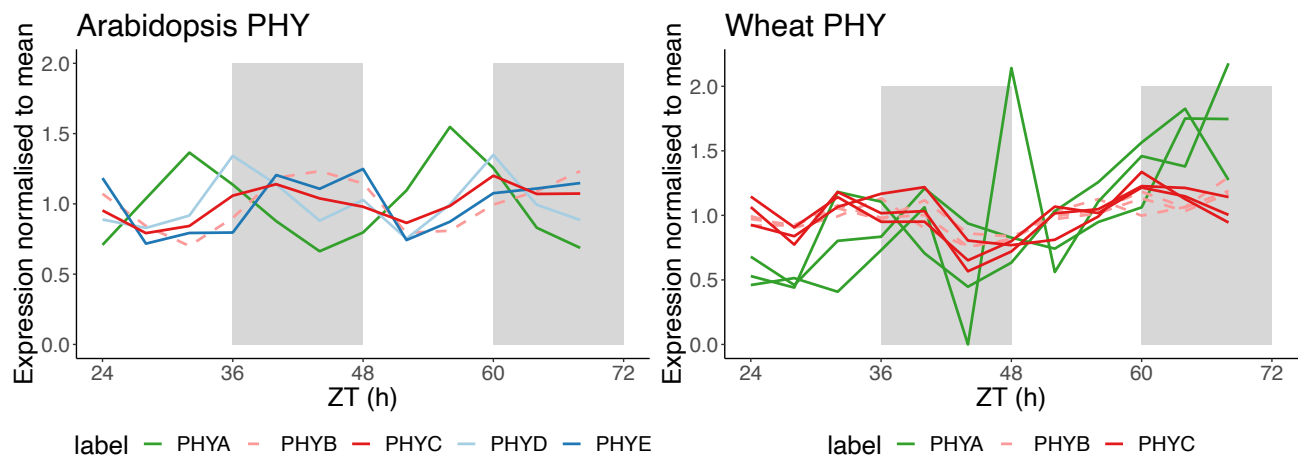

**C**

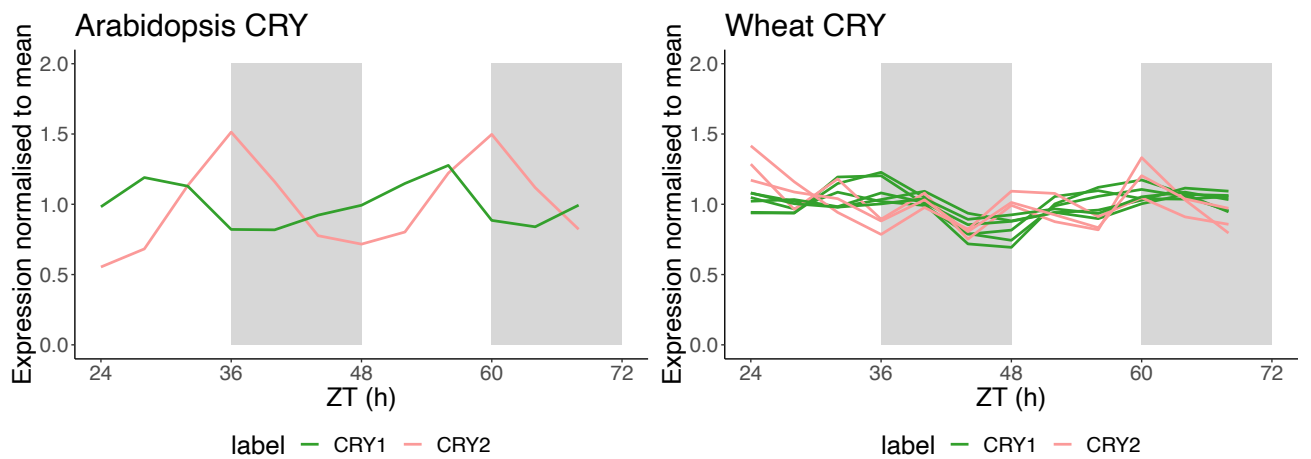

D

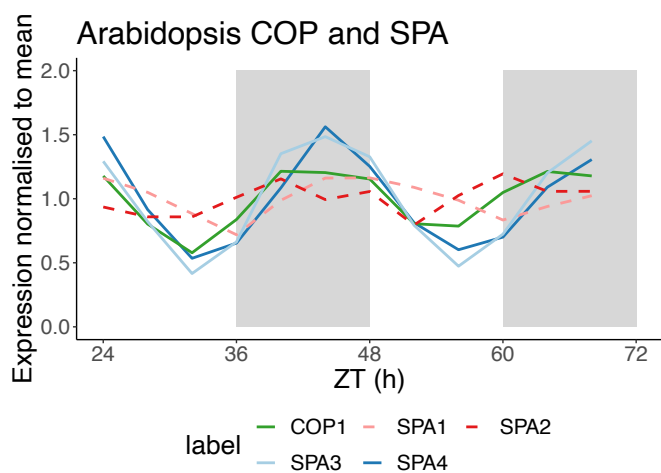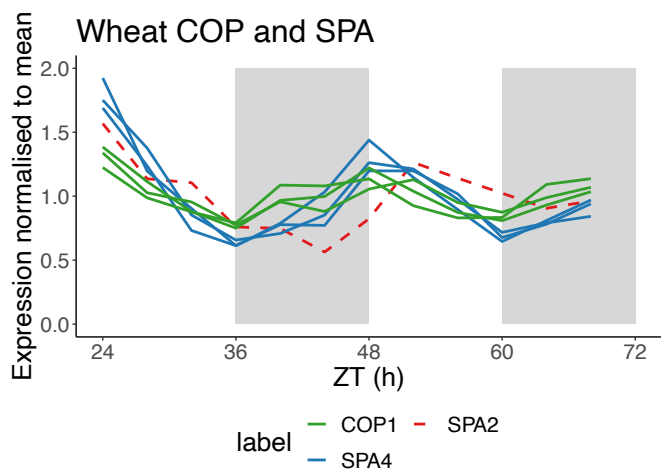

E

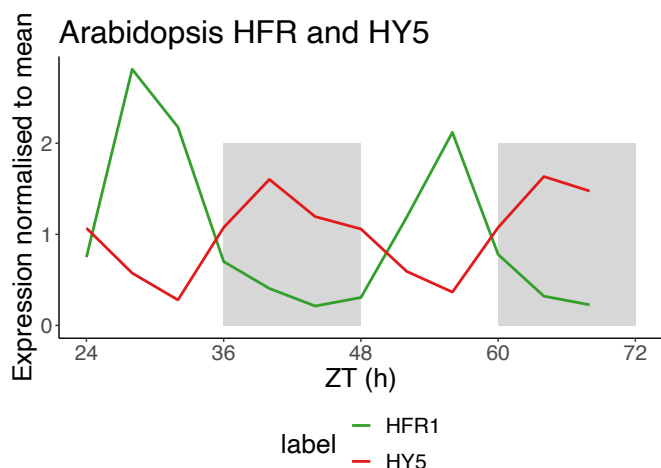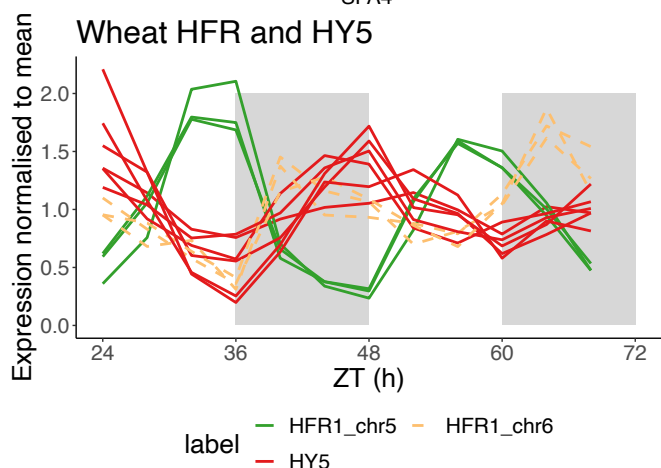

F

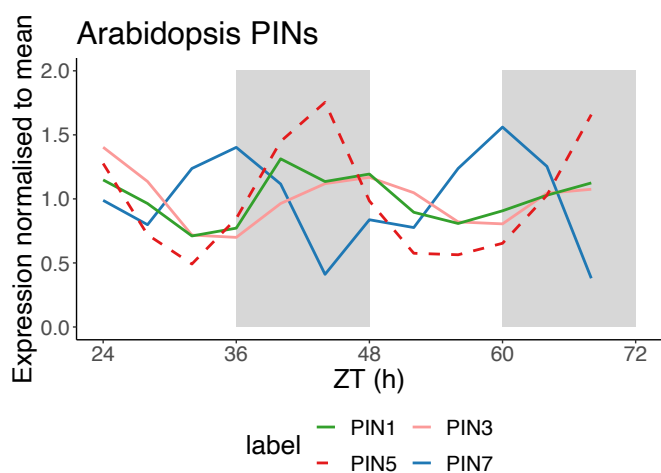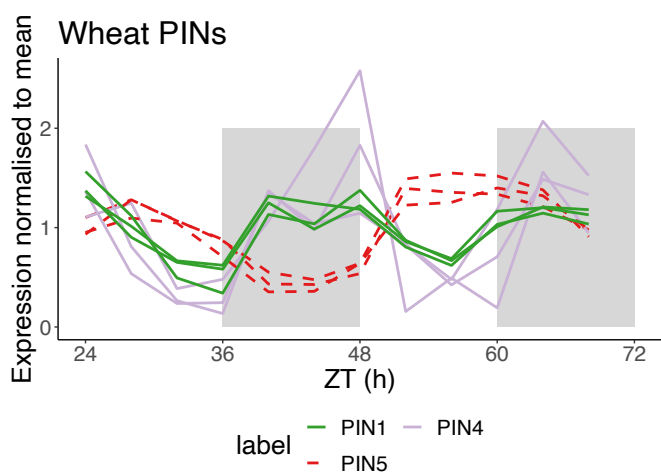

G

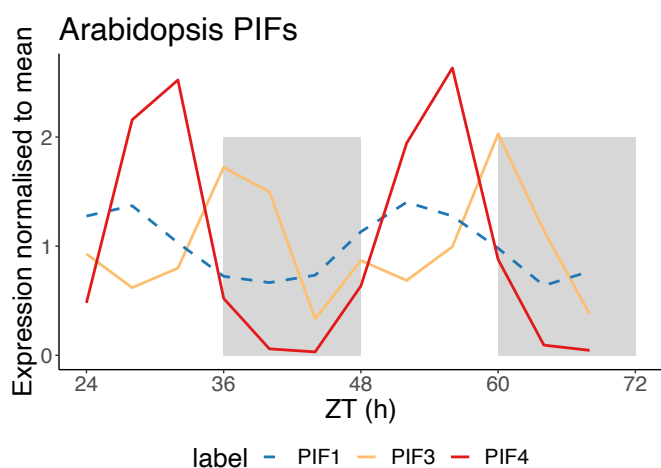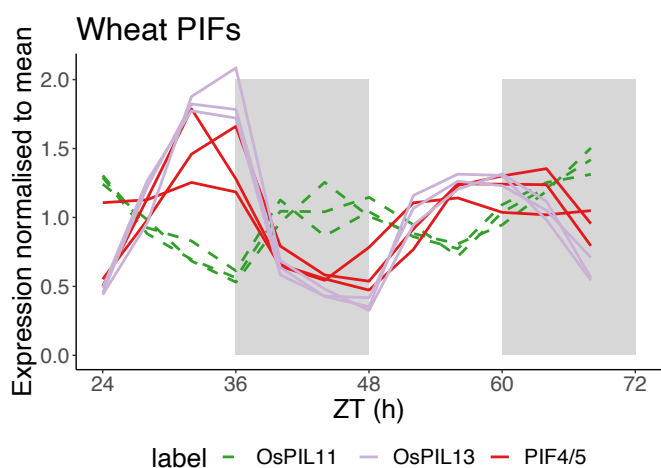

H

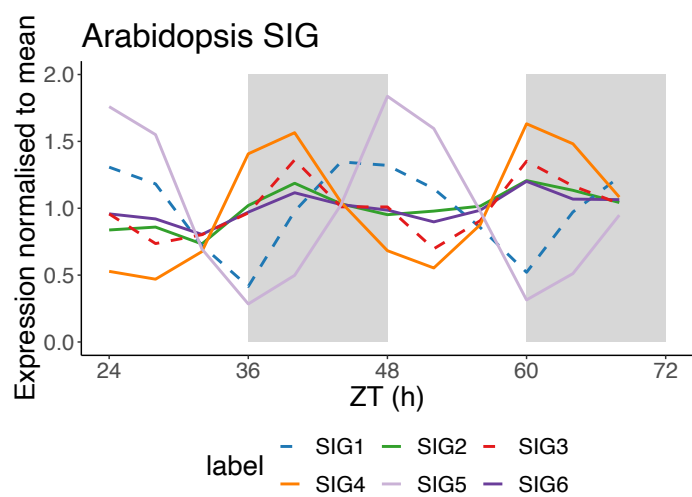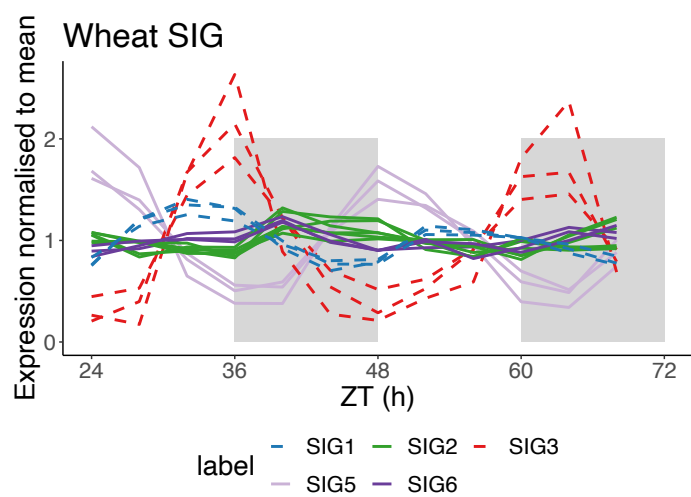

Supplement: S19 Fig — Data is mean normalised TPM. Shaded white and grey backgrounds indicate perceived day and night periods, respectively. Gene IDs for all genes plotted can be seen in S10 Table. (Data_Fig_S18-20 in S2 Data). (PDF) [file pbio.3001802.s027.pdf]

A

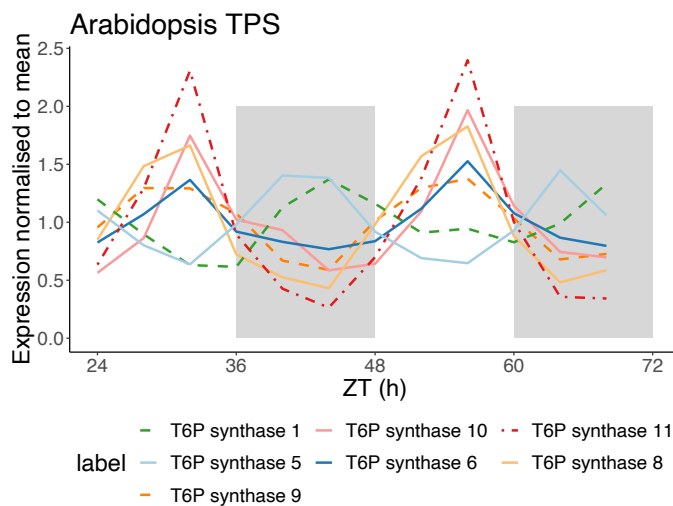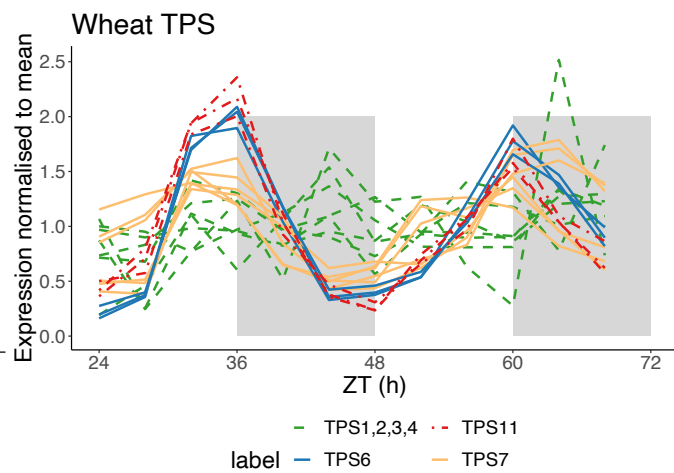

B

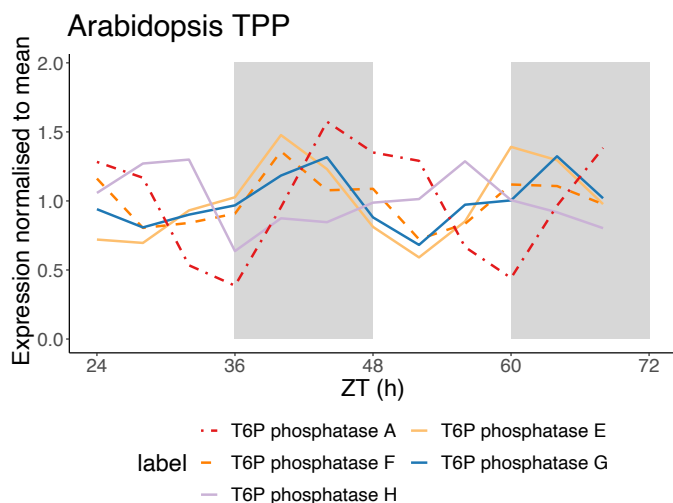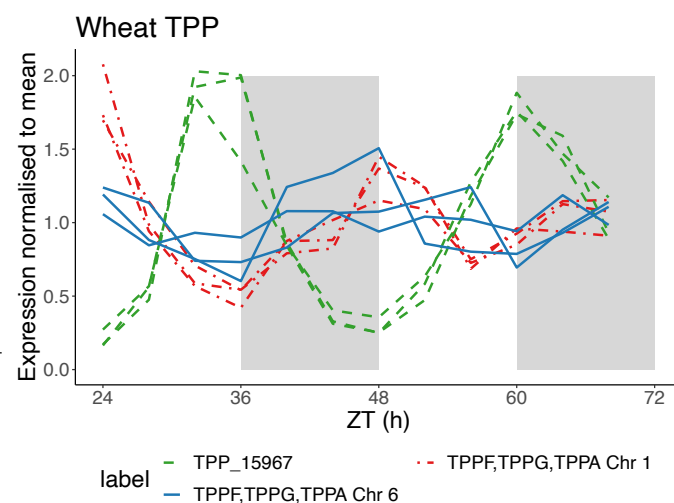

C

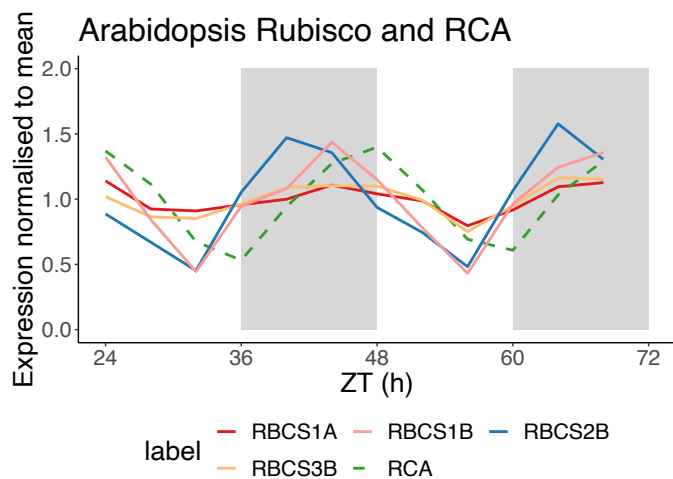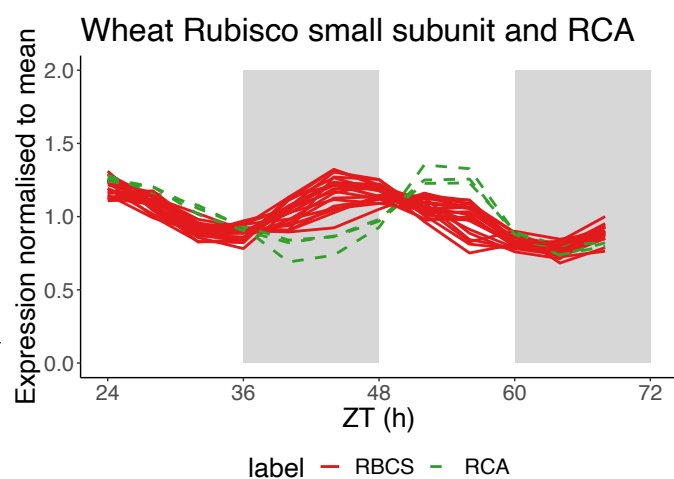

D

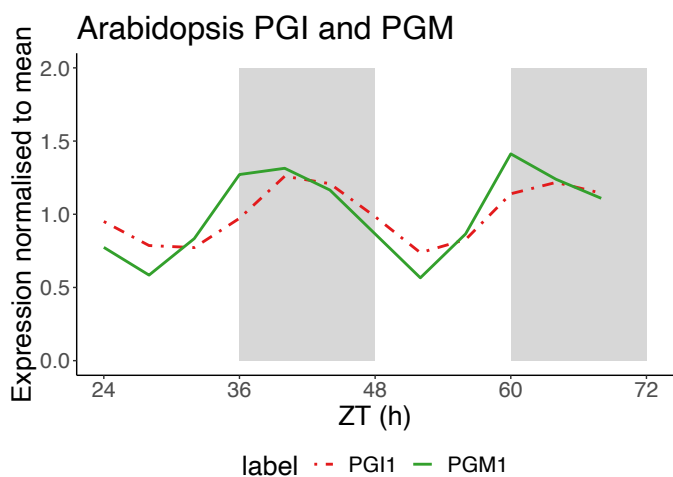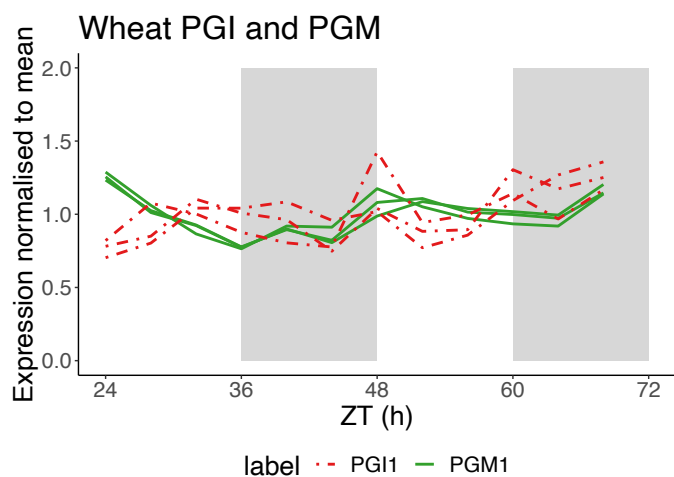

E

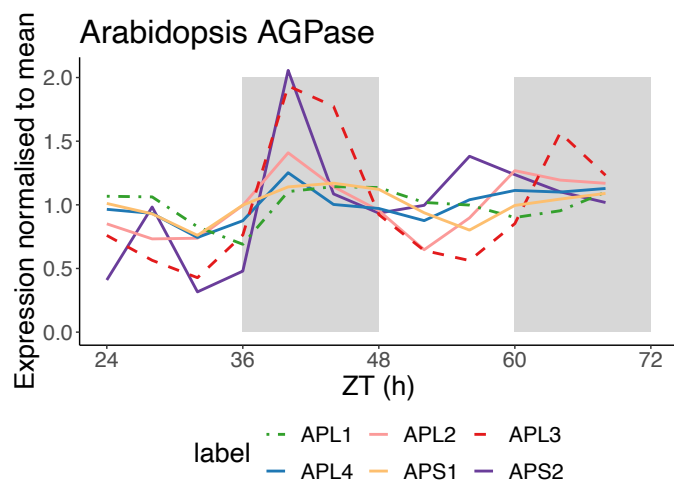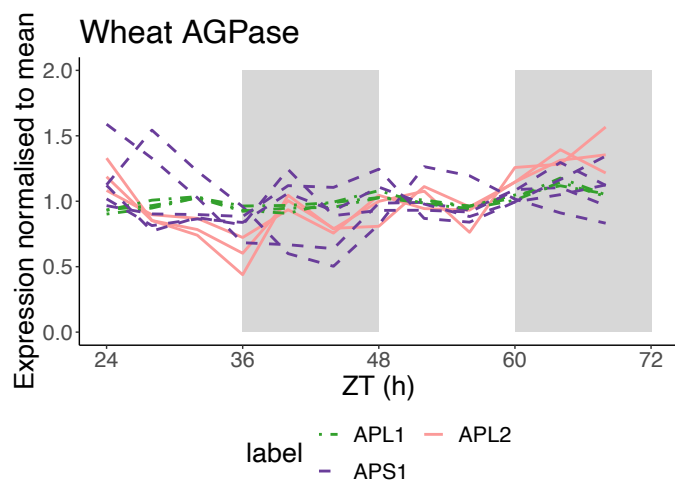

F

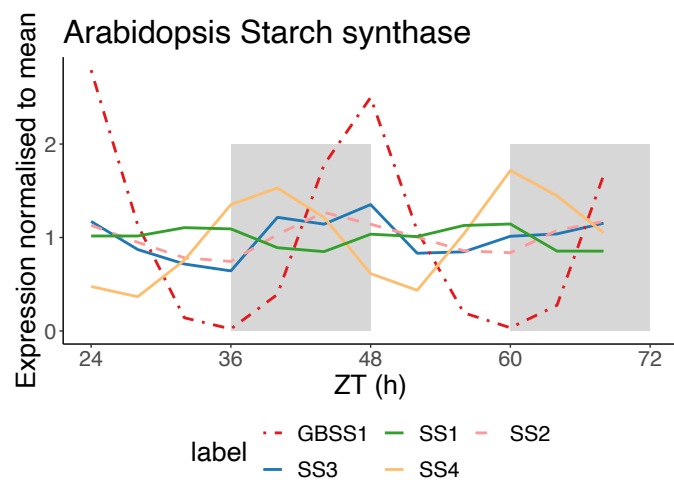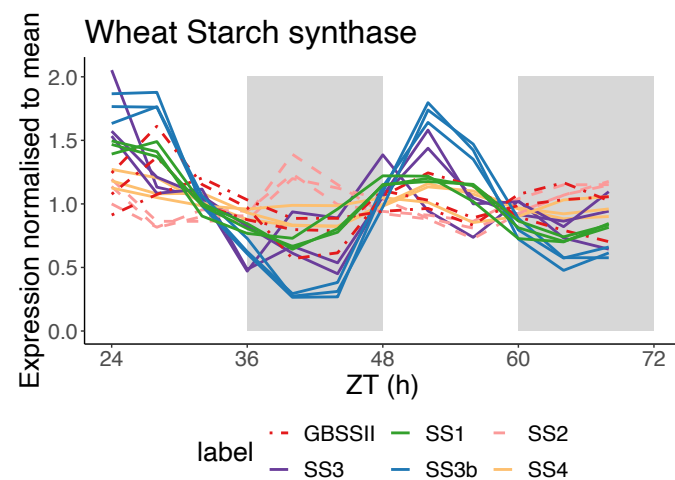

G

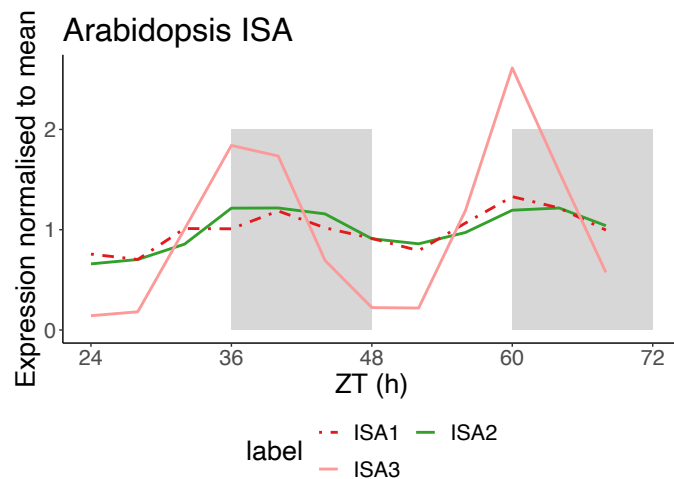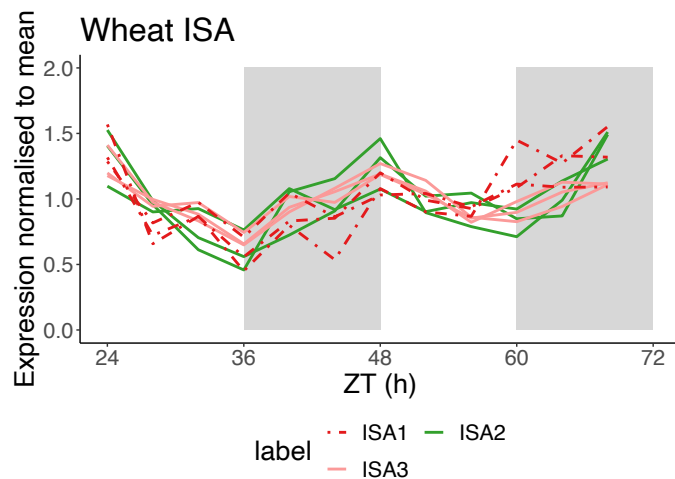

H

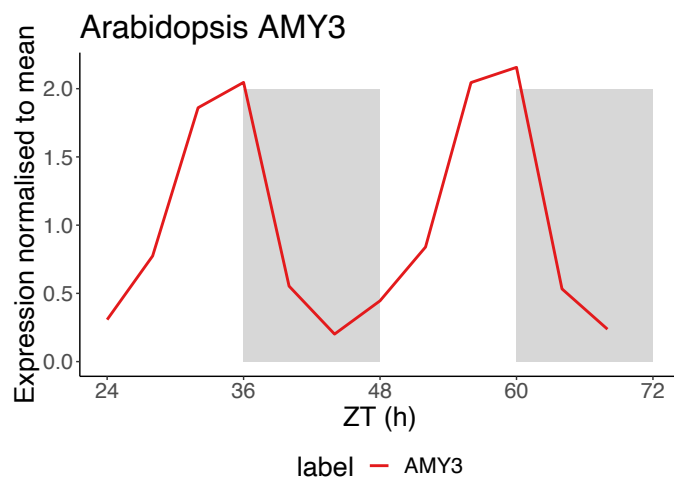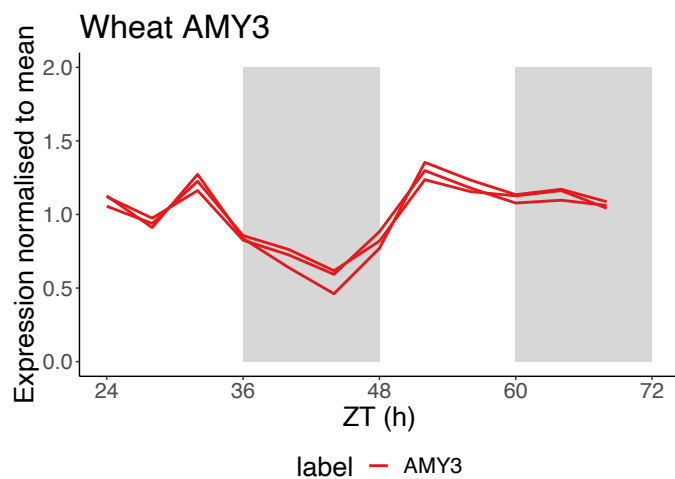

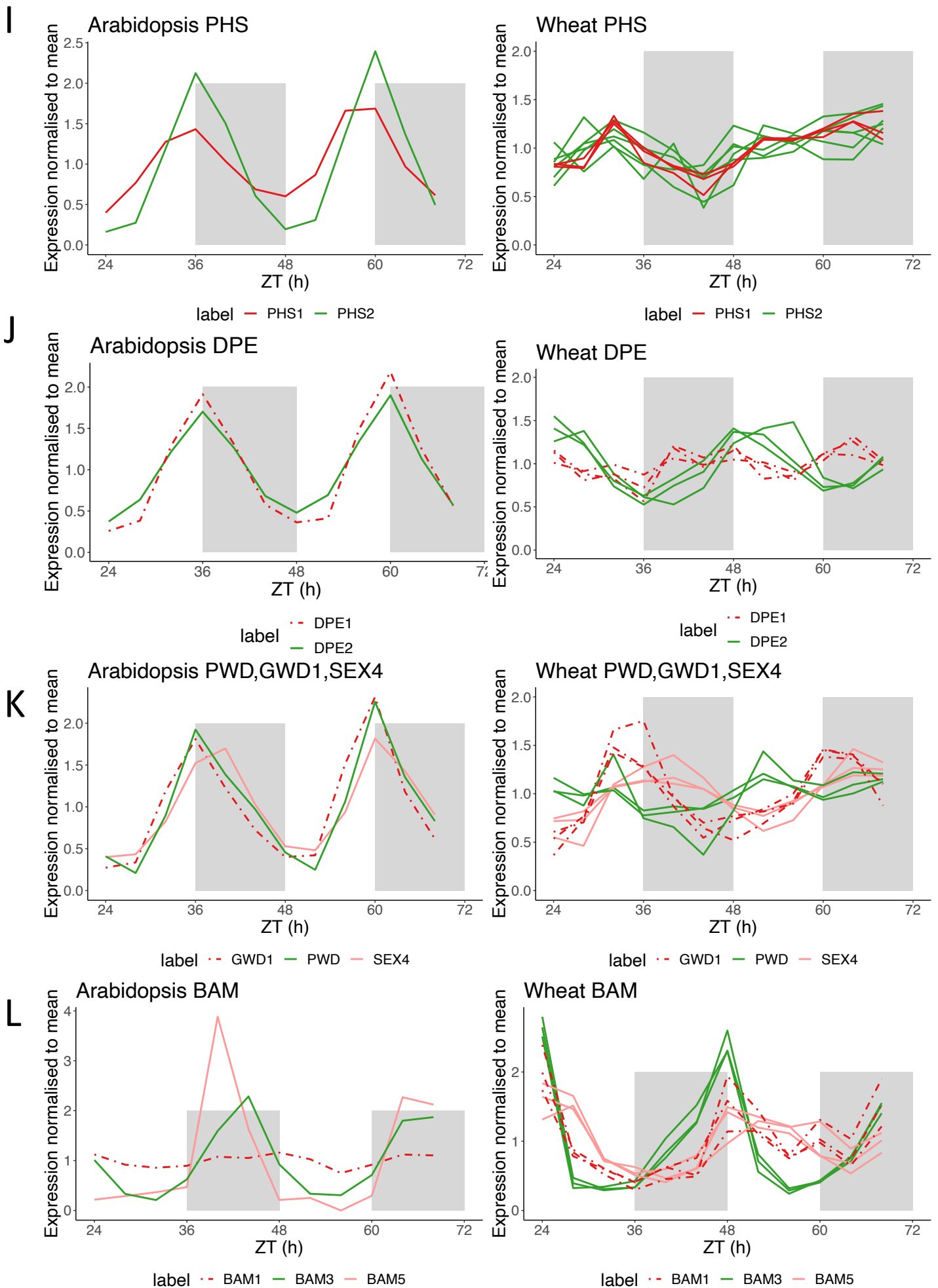

M

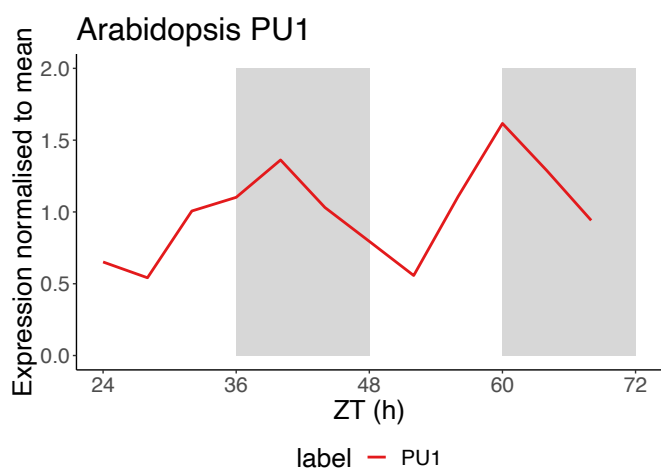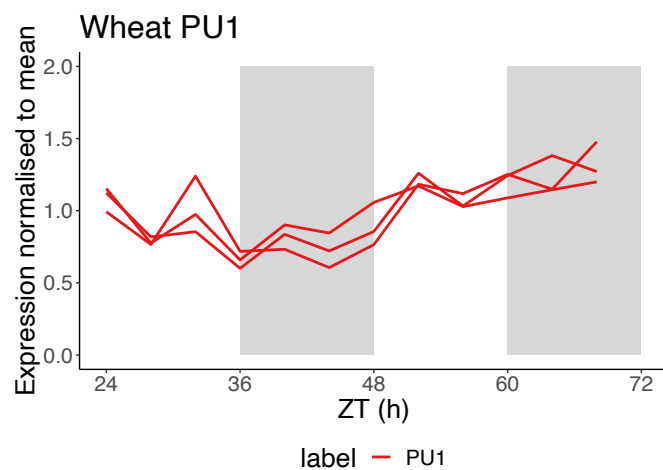

N

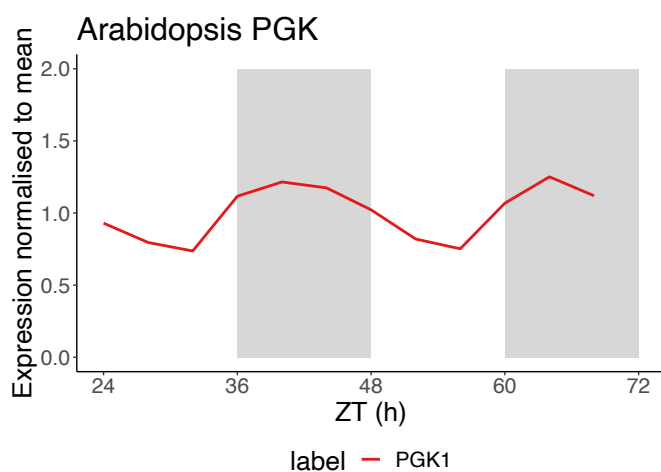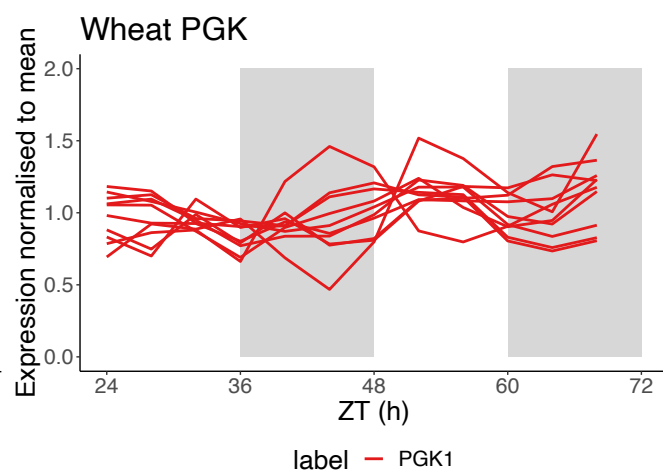

Supplement: S20 Fig — Data is mean normalised TPM. Shaded white and grey backgrounds indicate perceived day and night periods, respectively. Gene IDs for all genes plotted can be seen in S10 Table. (Data_Fig_S18-20 in S2 Data). (PDF) [file pbio.3001802.s028.pdf]
