# Supplementary material for: Circadian regulation of the transcriptome in a complex polyploid crop
Source: PLoS Biol. 2022 Oct 13;20(10):e3001802. doi: 10.1371/journal.pbio.3001802 (PMC9560141; doi:10.1371/journal.pbio.3001802)
Supplement: S8 Fig — Triads in the following categories are shown: 1A, 1B, 1D [triads with 1 rhythmic gene on the A, B, or D chromosomes, respectively and 2 arrhythmic genes], 2AB, 2AD, 2BD [triads with 2 rhythmic genes on the AB, AD, or BD chromosomes, respectively and 1 arrhythmic gene]. Points indicate that the homoeolog is rhythmic, and coloured lines represent the category of the triad. (PDF) [file pbio.3001802.s016.pdf]

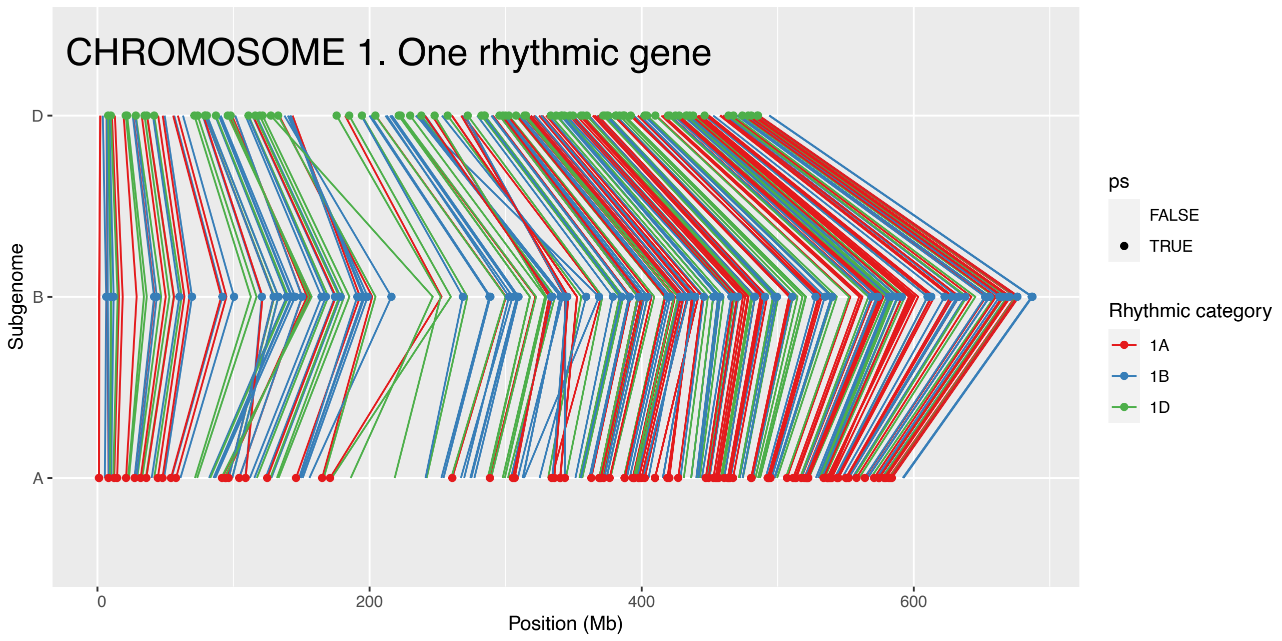
CHROMOSOME 1. One rhythmic gene
Subgenome
D
B
A
0
200
400
600
Position (Mb)
ps
FALSE
TRUE
Rhythmic category
1A
1B
1D

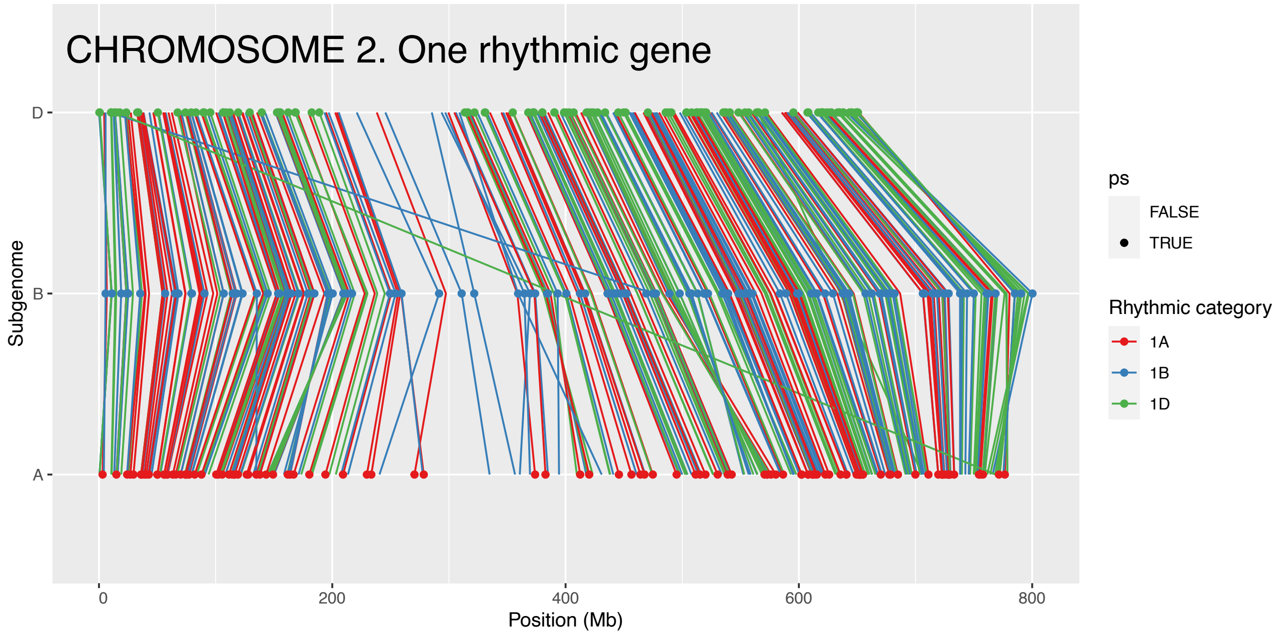
CHROMOSOME 2. One rhythmic gene
Subgenome
D
B
A
0
200
400
600
800
Position (Mb)
ps
FALSE
TRUE
Rhythmic category
1A
1B
1D

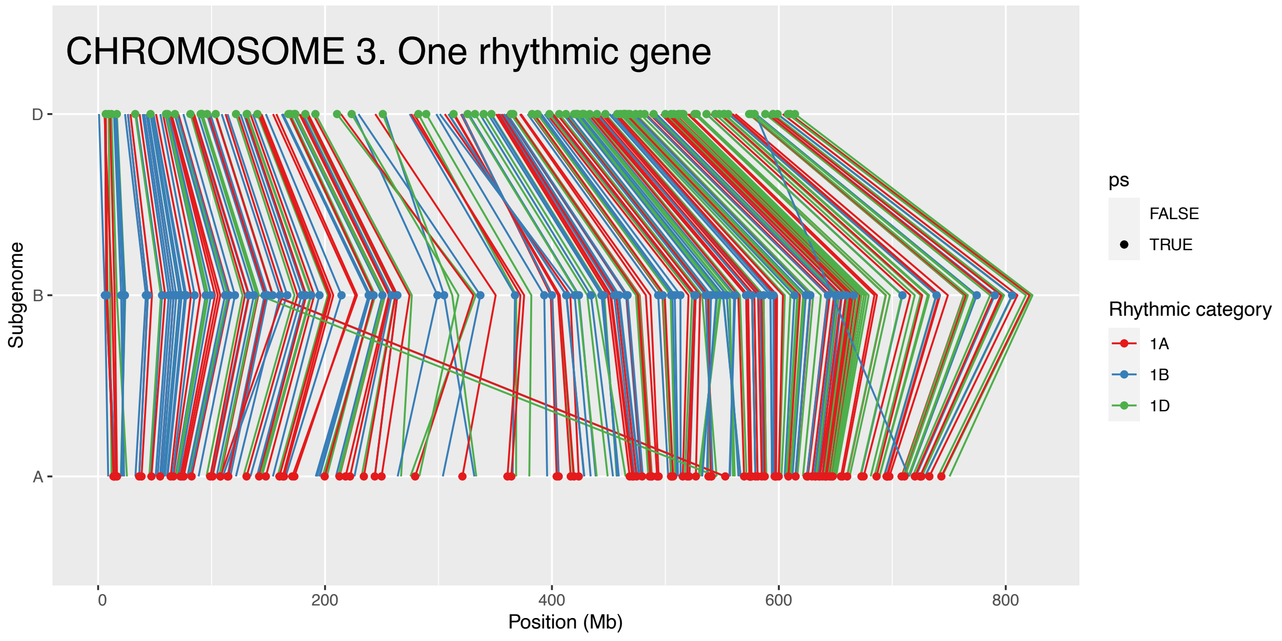
CHROMOSOME 3. One rhythmic gene
Subgenome
D
B
A
0
200
400
600
800
Position (Mb)
ps
FALSE
TRUE
Rhythmic category
1A
1B
1D

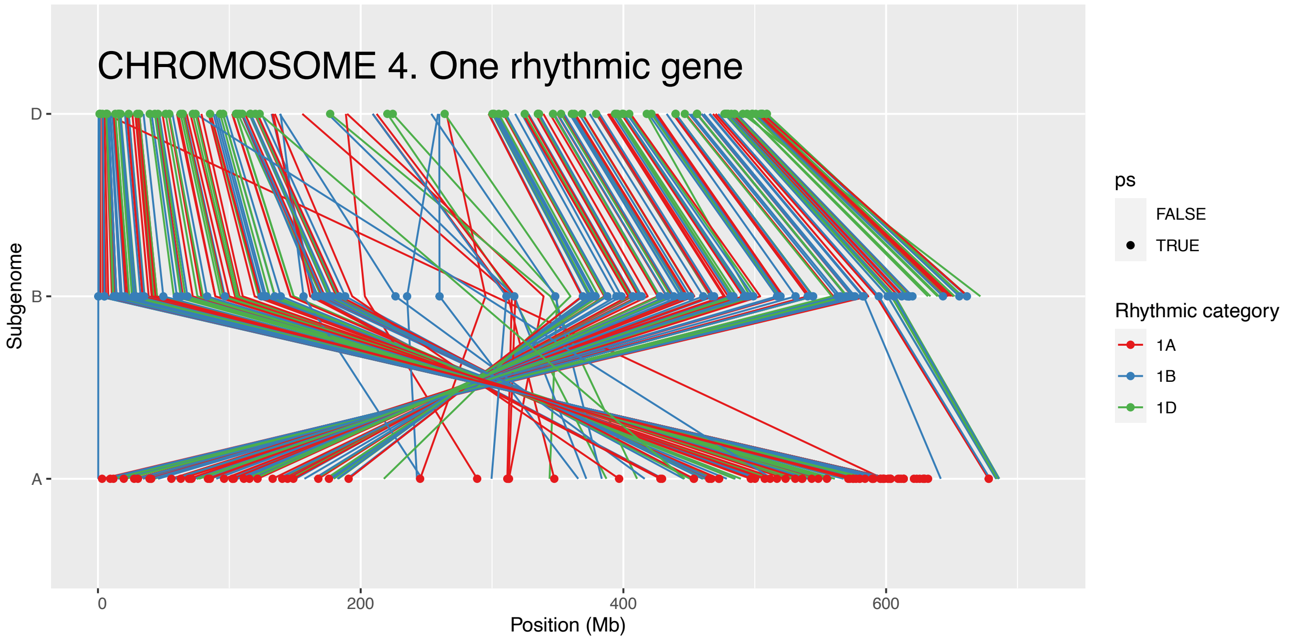
CHROMOSOME 4. One rhythmic gene
Subgenome
D
B
A
0
200
400
600
Position (Mb)
ps
FALSE
TRUE
Rhythmic category
1A
1B
1D

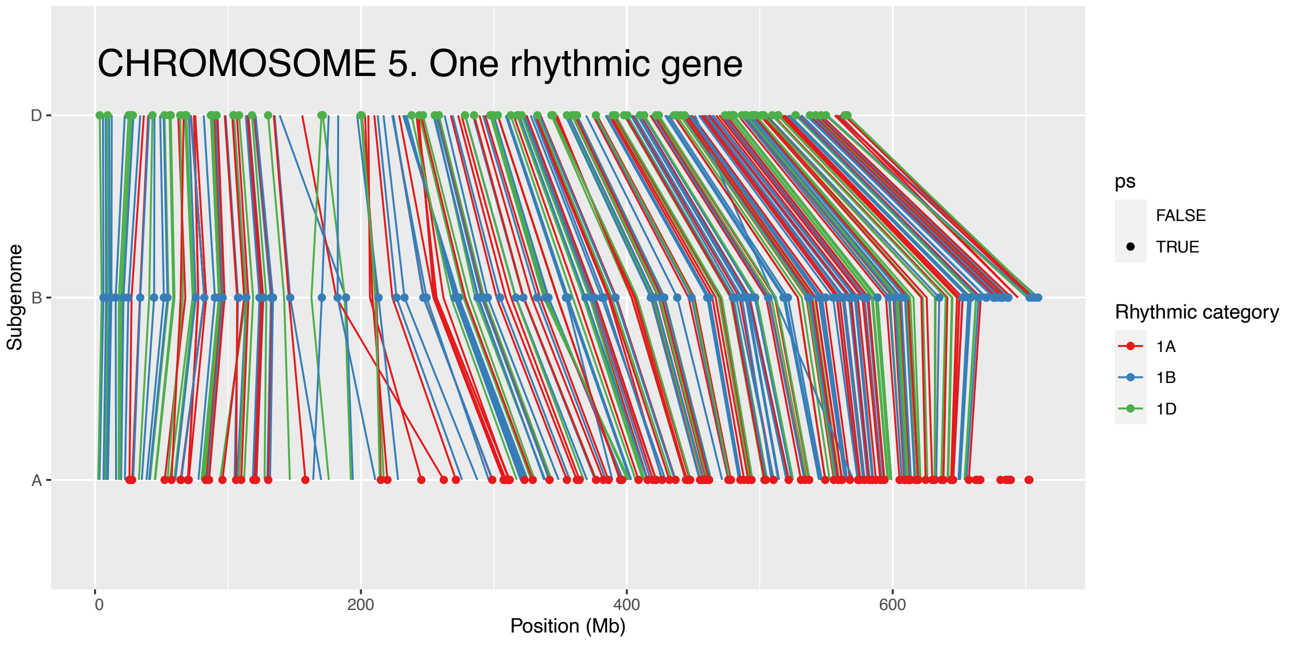
CHROMOSOME 5. One rhythmic gene
Subgenome
D
B
A
0
200
400
600
Position (Mb)
ps
FALSE
TRUE
Rhythmic category
1A
1B
1D

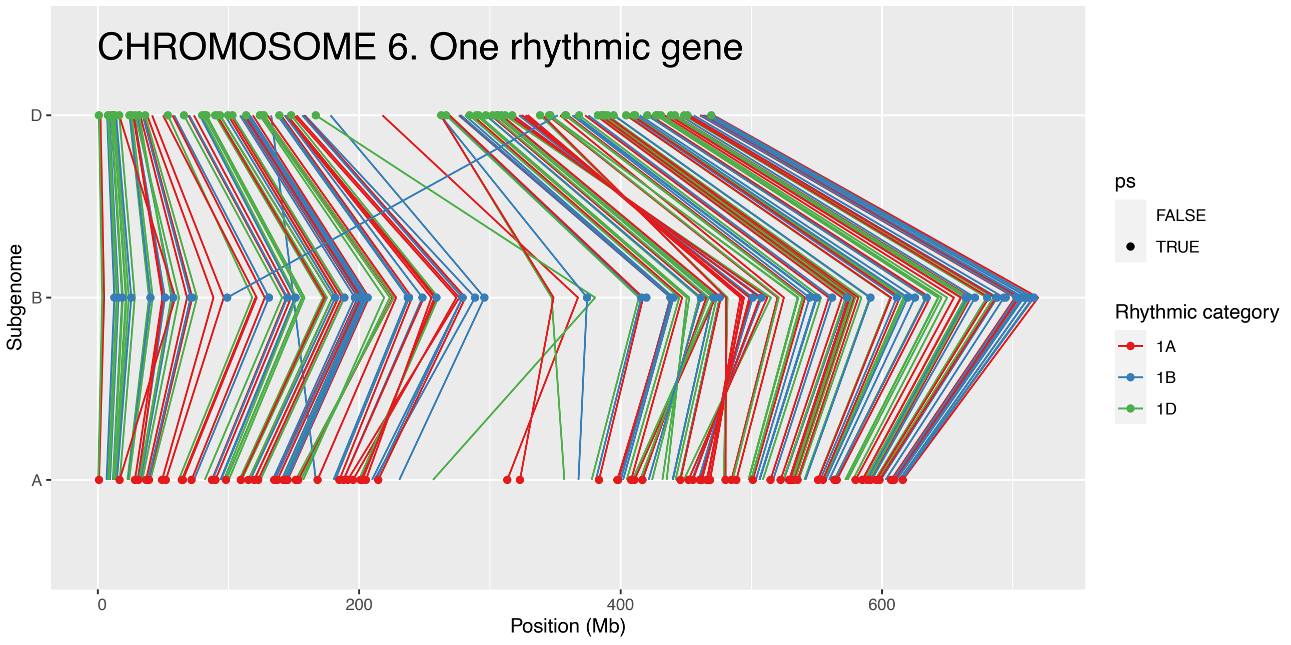
CHROMOSOME 6. One rhythmic gene
Subgenome
D
B
A
0
200
400
600
Position (Mb)
ps
FALSE
TRUE
Rhythmic category
1A
1B
1D

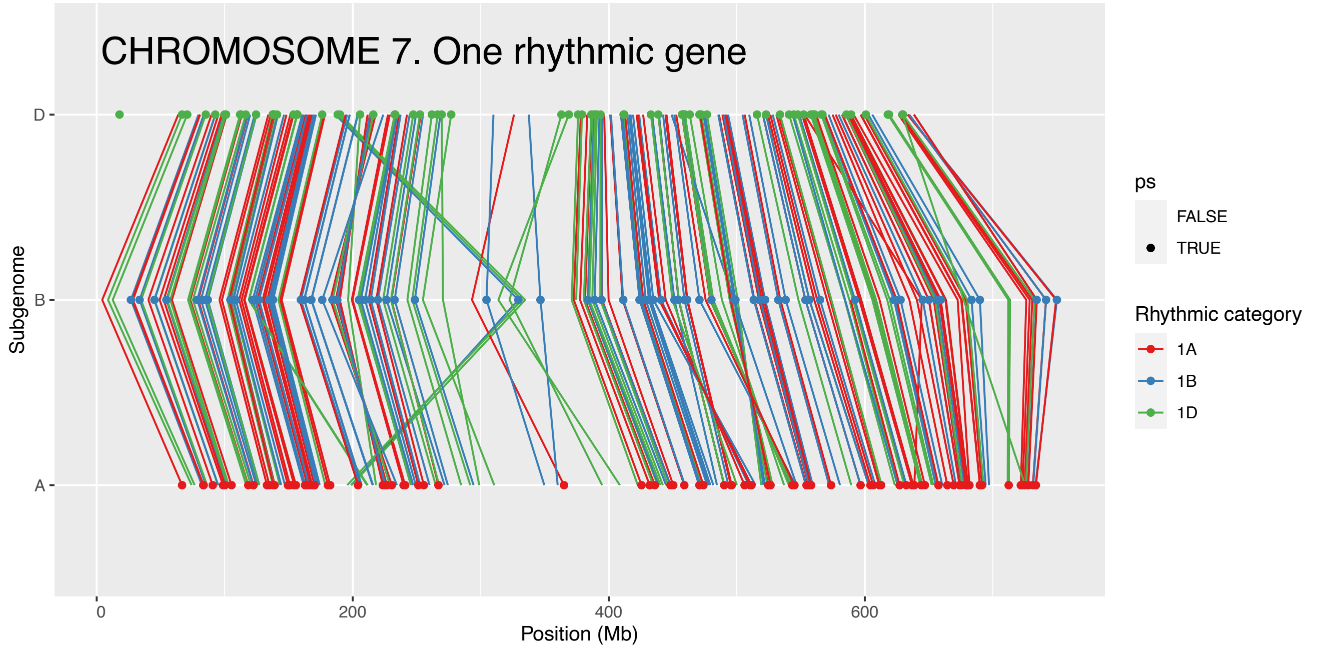
CHROMOSOME 7. One rhythmic gene
Subgenome
D
B
A
0
200
400
600
Position (Mb)
ps
FALSE
TRUE
Rhythmic category
1A
1B
1D

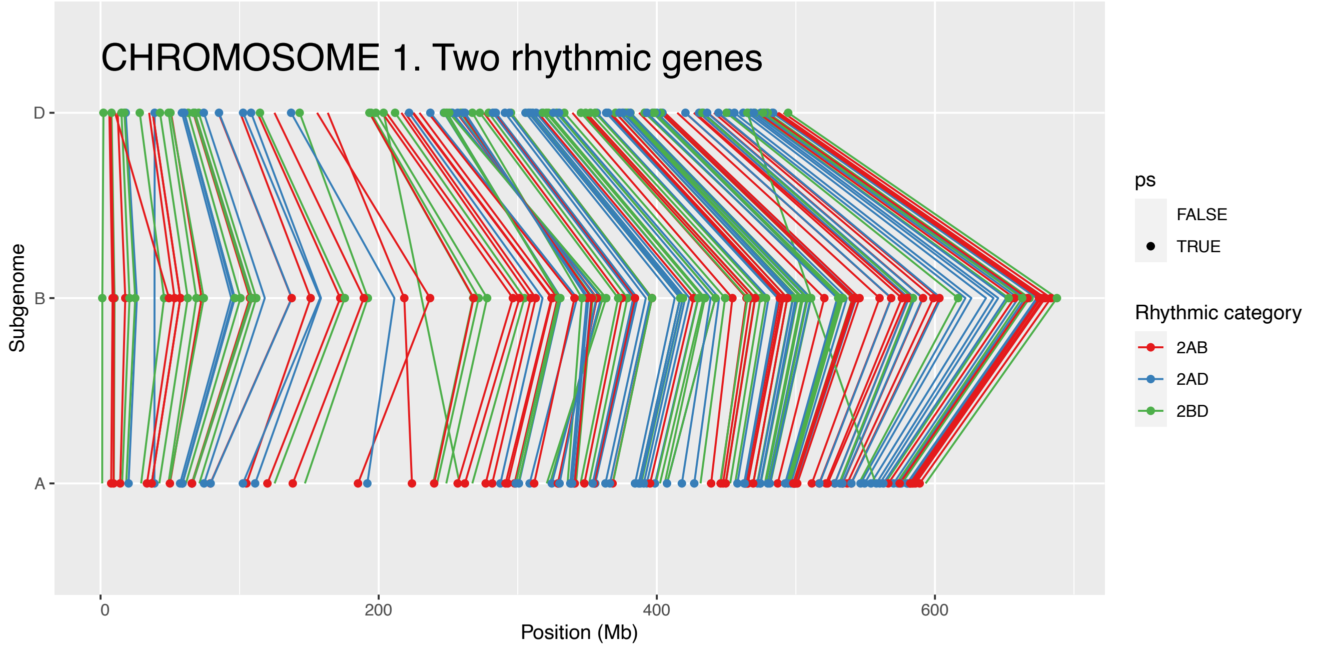
CHROMOSOME 1. Two rhythmic genes
Subgenome
D
B
A
0
200
400
600
Position (Mb)
ps
FALSE
TRUE
Rhythmic category
2AB
2AD
2BD

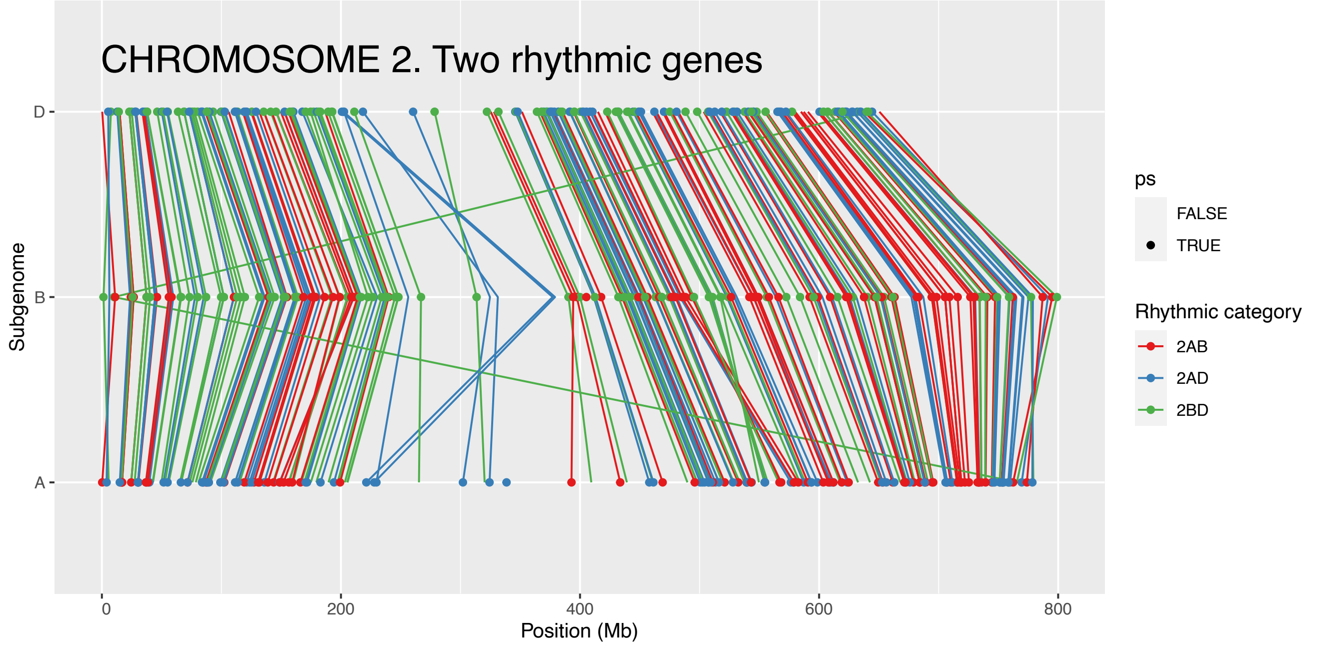
CHROMOSOME 2. Two rhythmic genes
Subgenome
D
B
A
0
200
400
600
800
Position (Mb)
ps
FALSE
TRUE
Rhythmic category
2AB
2AD
2BD

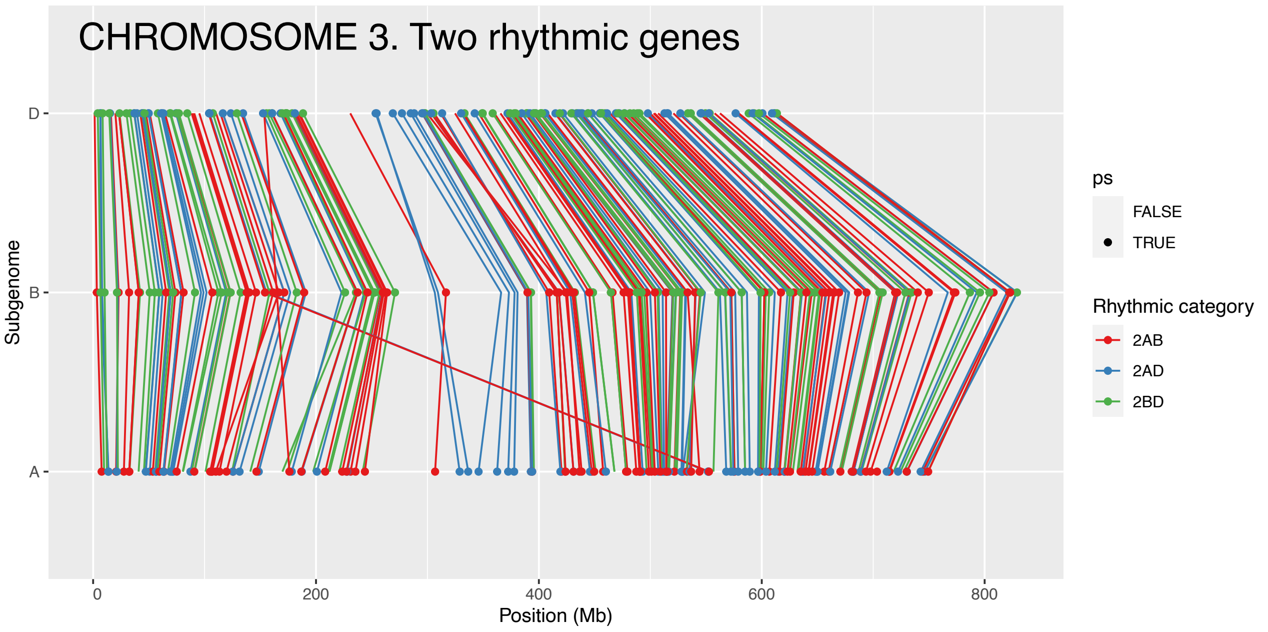

CHROMOSOME 3. Two rhythmic genes
D
B
A
Subgenome
0
200
400
600
800
Position (Mb)
ps
FALSE
TRUE
Rhythmic category
2AB
2AD
2BD

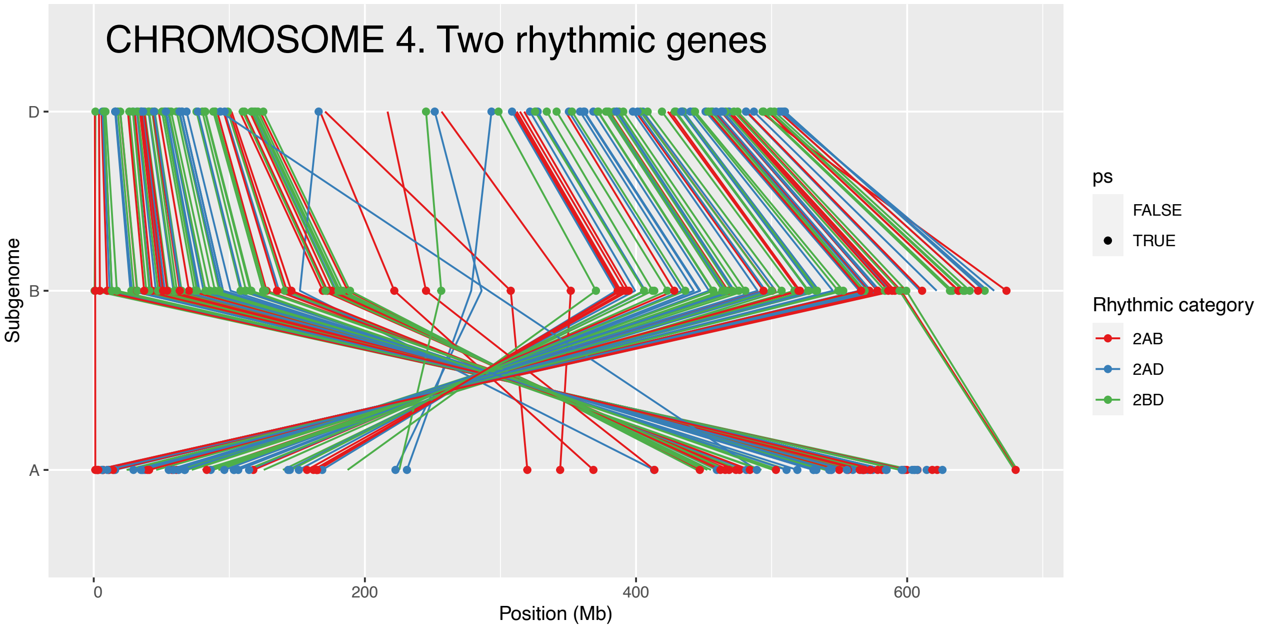

CHROMOSOME 4. Two rhythmic genes
D
B
A
Subgenome
0
200
400
600
Position (Mb)
ps
FALSE
TRUE
Rhythmic category
2AB
2AD
2BD

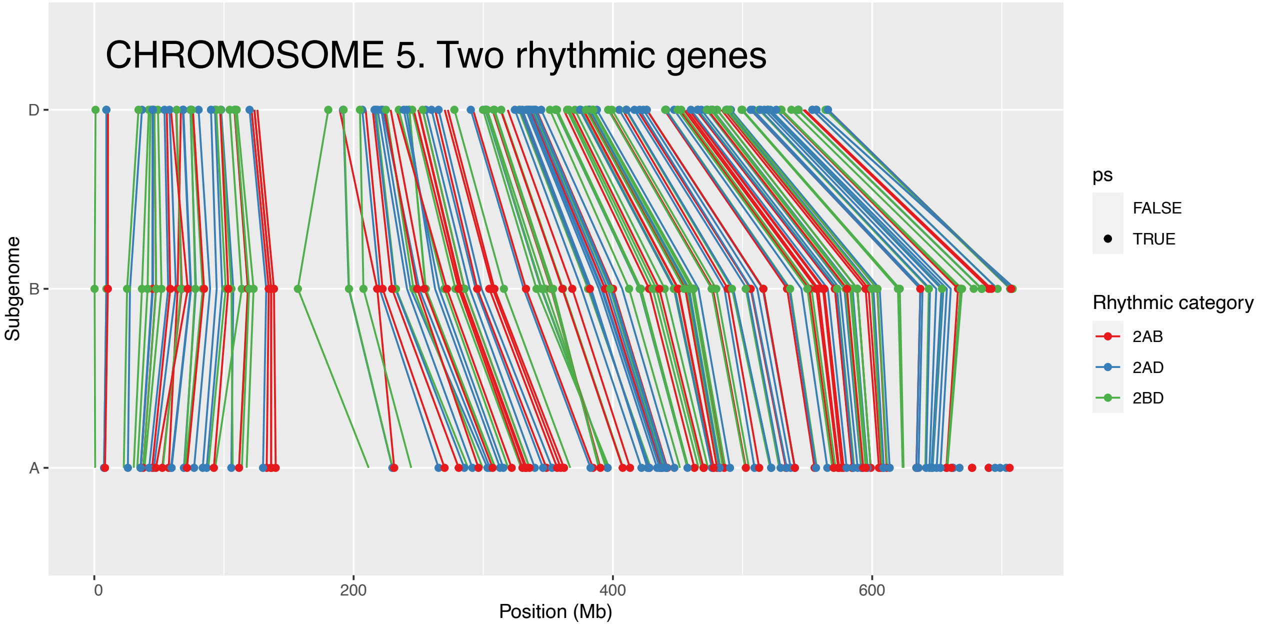

CHROMOSOME 5. Two rhythmic genes
D
B
A
Subgenome
0
200
400
600
Position (Mb)
ps
FALSE
TRUE
Rhythmic category
2AB
2AD
2BD

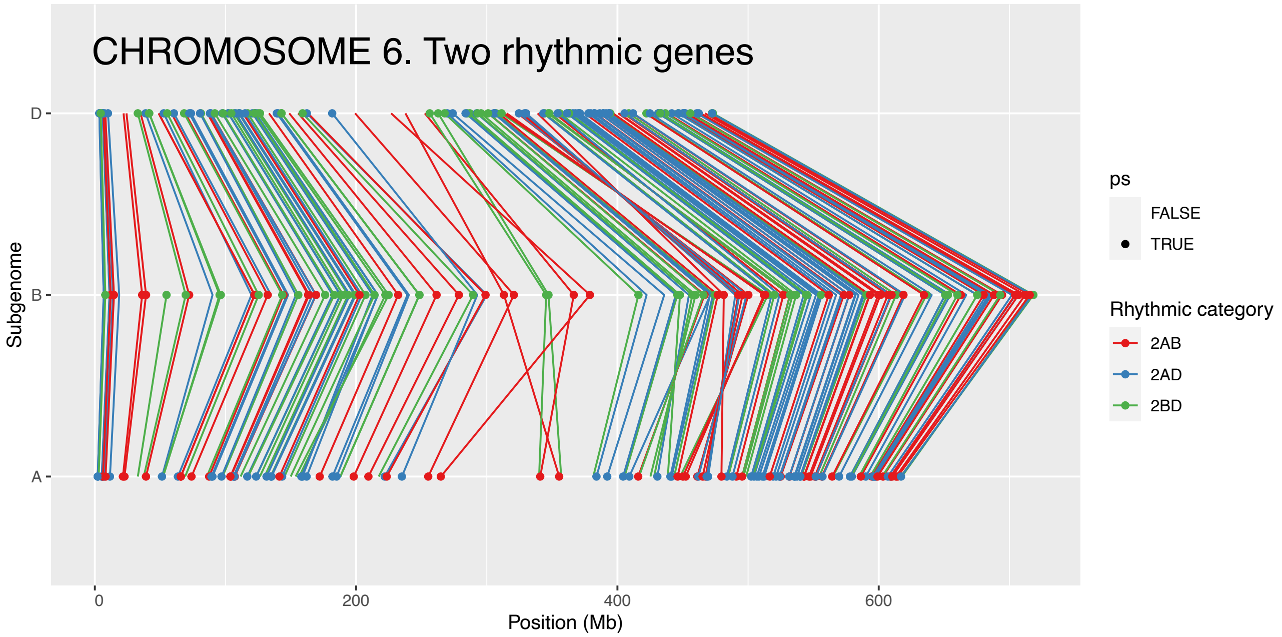
CHROMOSOME 6. Two rhythmic genes
D
B
A
Subgenome
0
200
400
600
Position (Mb)
ps
FALSE
TRUE
Rhythmic category
2AB
2AD
2BD

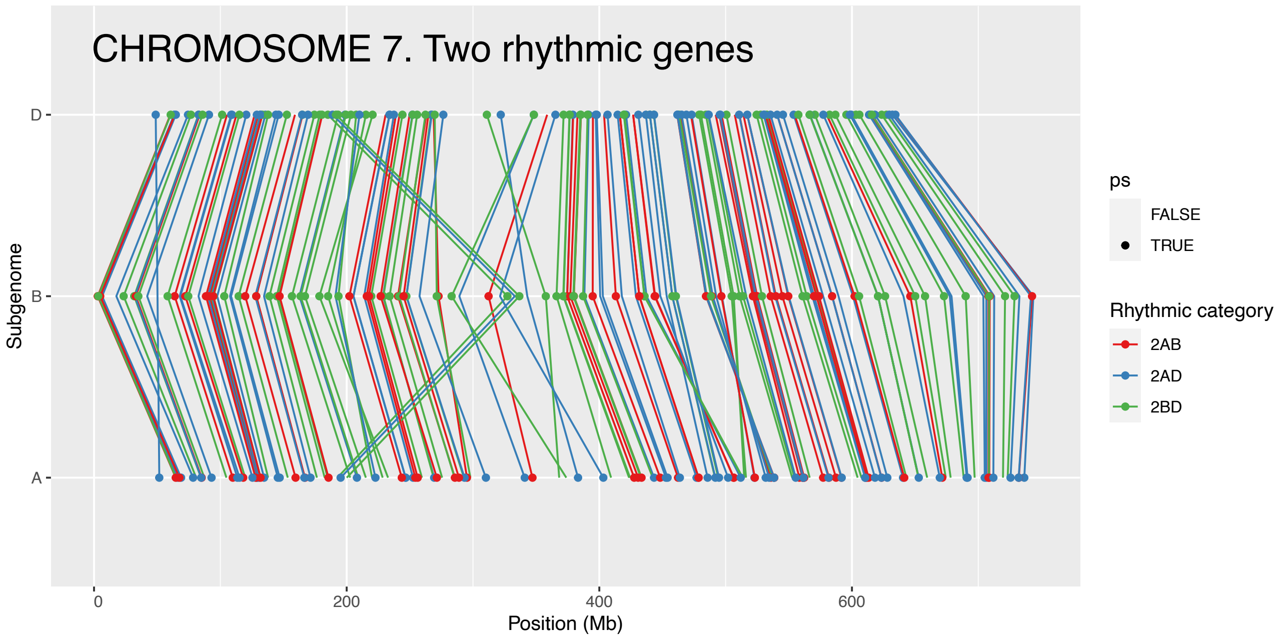
CHROMOSOME 7. Two rhythmic genes
D
B
A
Subgenome
0
200
400
600
Position (Mb)
ps
FALSE
TRUE
Rhythmic category
2AB
2AD
2BD
