# Supplementary material for: Circadian regulation of the transcriptome in a complex polyploid crop
Source: PLoS Biol. 2022 Oct 13;20(10):e3001802. doi: 10.1371/journal.pbio.3001802 (PMC9560141; doi:10.1371/journal.pbio.3001802)
Supplement: S9 Fig — Numbers of significantly enriched (p < 0.05) GO-slim terms in common between 9 pairwise modules in Arabidopsis and wheat were counted and the pairwise modules were grouped based on the highest correlation score (peak lag) following cross-correlation with a lag of 0, 4, 8, or 12 h. (Data_Fig_S9 in S2 Data). (PDF) [file pbio.3001802.s017.pdf]

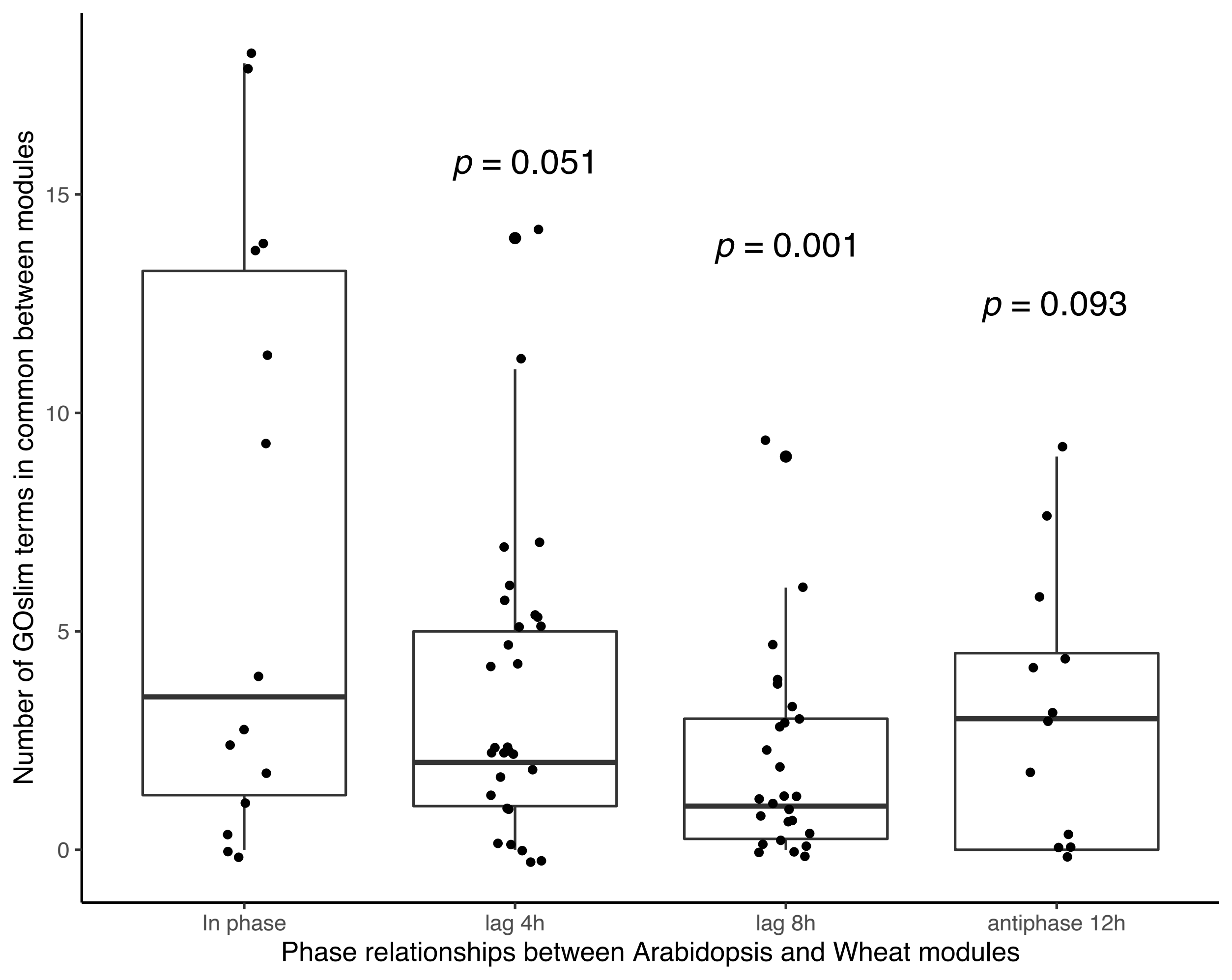

p = 0.051
p = 0.001
p = 0.093
15
10
5
0
Number of GOslim terms in common between modules
In phase
lag 4h
lag 8h
antiphase 12h
Phase relationships between Arabidopsis and Wheat modules
