# Supplementary material for: Circadian regulation of the transcriptome in a complex polyploid crop
Source: PLoS Biol. 2022 Oct 13;20(10):e3001802. doi: 10.1371/journal.pbio.3001802 (PMC9560141; doi:10.1371/journal.pbio.3001802)
Supplement: Attachment — Submitted filename: 125671_1_rebuttal_2364712_rc1m1m.docx [file pbio.3001802.s043.docx]

**Manuscript number:** RC-2022-01379

**Corresponding author(s):** Anthony, Hall

*[Please use this template only if the submitted manuscript should be considered by the affiliate journal as a full revision in response to the points raised by the reviewers.*

*If you wish to submit a preliminary revision with a revision plan, please use our "*[*Revision Plan*](https://docs.google.com/document/u/0/d/1HHp62Tfeb44nd3Gu_3ChB0b8gmMdEog7GjZcZFwHSiQ/edit)*" template.* ***It is important to use the appropriate template to clearly inform the editors of your intentions.****]*

## 1. General Statements [optional]

Thank you for considering our manuscript “**Circadian regulation of the transcriptome in a complex polyploid crop**” and for inviting us to submit a revised version. Overall, all three reviewers provided encouraging feedback that the analysis is thorough and will be of interest to readers. Reviewer 1 states that: “Interestingly, by analyzing circadian parameters among expressed genes, they find evidence that this newly formed allopolyploid already shows signs of divergence in circadian traits among homoeologs” and predicts that: “This dataset will be a great resource for the community and enable new predictions about the influence of polyploidy on the circadian control of important crop improvement traits and the circadian regulation of gene expression.” Reviewer 2 describes our work as “precious, carefully generated and analysed dataset” and Reviewer 3 finds the manuscipt to be “well written, the methods are correct, the analysis performed is sufficiently extensive and the figures are clear.”

This new manuscript resolves all comments made by the reviewers regarding our earlier submission and our point-by-point response to the reviewers is provided below. Our study will appeal to cereals communities, to groups investigating genome evolution and polyploidy, to circadian biologists, and to crop breeders. In this respect, our study provides insights that are relevant specifically for wheat research, and more broadly for the investigation of polyploid genomes. This study is especially timely because there is believed to be considerable scope for the application of circadian biology to agriculture (Steed et al., 2021).

Steed, G., Ramirez, D. C., Hannah, M. A. & Webb, A. A. R. Chronoculture, harnessing the circadian clock to improve crop yield and sustainability. *Science (80-. ).* **372**, (2021).

## 2. Point-by-point description of the revisions

*This section is mandatory.* *Please insert a point-by-point reply describing the revisions that were already carried out and included in the transferred manuscript.*

From the start, the authors would like to thank all the reviewers for their careful and constructive consideration of our manuscript. We have now made several changes to the paper and believe it to be better for the feedback.

**Reviewer #1 (Evidence, reproducibility and clarity (Required)):**

In this study, Rees et al. perform an RNA-seq circadian time course experiment in the recently formed allopolyploid wheat. Through comparisons with other circadian transcriptomic datasets in other species it appears that the period of rhythmic genes is much more variable in wheat with a shift to longer periods compared to the other species examined. Interestingly, by analyzing circadian parameters among expressed genes, they find evidence that this newly formed allopolyploid already shows signs of divergence in circadian traits among homoeologs. A thorough comparison with circadian regulated genes in Arabidopsis reveals overlap in phasing of genes involved in certain biological processes such as photosynthesis and light signaling whereas genes involved in starch metabolism were found to have different levels of rhythmicity and phasing. This dataset will be a great resource for the community and enable new predictions about the influence of polyploidy on the circadian control of important crop improvement traits and the circadian regulation of gene expression.

Major Comments

1. The results section starts with very little explanation of the experiment. It would help to provide a little more detail at the start of the results to explain the context for the experiment and what was done, when samples were collected and for how long. For the methods section, it isn't until line 650 that it is clearly stated that the sampling started at ZT0. It would be better to put this in the plant materials and growth condition section.

Thank you for highlighting the need for this context, we agree that the manuscript is improved by an introduction to the experiments. We have now included an “Experimental context” section in the results and have taken the opportunity to explain how the full 0-68h and 24-68h datasets are used within our analysis. Ln 74-82. We have also edited the Methods as suggested Ln 610-615.

2. The low proportion of circadian regulated genes is likely due to the very low cutoff for calling a gene expressed, especially when there are three days of repeated timepoints. If a gene is expressed across the time course it should have values above TPM 0 for at least 3 time points in order for it to be expressed each day. I'd also be suspicious of a gene with a TPM value less than 0.5. Comparing these types of numbers is always challenging due to the various cutoffs used. Along those lines, why was a different filtering scheme used for Arabidopsis (line 657)?

We completely agree that the proportion of genes described as rhythmic changes a great deal with the threshold at which you exclude low expression transcripts as well as the window over which measurements are taken and the *q*-value cut-off for rhythmicity. We performed an analysis to test the effects of applying a pre-filtering step to exclude low-expression genes and discuss our findings in Supplementary Note 1. Briefly, we removed genes with expression less than 0.1 TPM in six or more timepoints and again ran Metacycle to define numbers of rhythmic genes. Our results are discussed in Supplementary Note 1 and are presented in Supplementary Table 1. Regardless of the cut-offs applied, *Arabidopsis* and wheat data was treated identically, and our findings reported in the main results were consistent with those reported in the Supplementary analysis. Thank you for raising this point, as we have now improved our description of this analysis in the main text (Ln 92-95).

Regarding the different filtering schemes, the filtering mentioned by Reviewer 1 was applied to both *Arabidopsis* and wheat data for a stricter retention of rhythmic genes, as part of the pre-WGCNA clustering analysis. Filtering to retain genes with >0.5TPM across 3 timepoints was applied to reduce lowly expressed genes, that act as background 'noise' when defining clusters. We applied this across 3 timepoints rather than the WGCNA suggestion of 90% of samples - because the patterns of expression in our rhythmically filtered datasets were cyclical in nature.

3. In reference to the shortening of the period every day, this should be interpreted with caution. Period estimate of a single cycle are not very reliable and the SD for each day is around 3h so it is difficult to draw any conclusions about changes in period each day. One option would be to only include genes with an SD less than 1h or alternatively to remove the discussion surrounding the comparison of period across the three days and focus on the period results for the full 24h-68h window shown in 1b. While 2 days is better it is still not ideal for calling period; however, your first day will still have a strong diurnal driven pattern that will likely skew your circadian period.

Thank you for your comments. Our question here was to determine whether the mean period lengths of rhythmic transcripts in wheat were always immediately longer upon transfer to constant light, or whether they got progressively longer over time. Upon reading the reviewer’s comment, we realize that the explanation provided of how we conducted this analysis was misleading. Our approach was to take a 44h sliding window (almost 2 days) and measure period at 0-44h, 12-56h and 24-68h. We have now added the previously missing statistics that support our findings in the main text, and which hopefully show the significance of the period changes over time (supplementary note 2). One of the most surprising findings from this analysis was that the periods in the first window were the longest 28.61h (SD=3.421), suggesting that the diel (driven) oscillation had little impact upon immediate transfer to free run. Our interpretation is that the mean period initially lengthens trying to follow the missing dusk signal, before the free-running endogenous period asserts itself in later cycles (Ln 129-128).

4. Line 87-93: If the dusk cue is important for clock expression you would think this would be biased towards genes that peak later in the day or near dusk. This argument should be connected better to the period results discussed on lines 98-101.

Following on from our statement above, we have now combined our hypothesis for why wheat transcripts expressed at dusk have longer periods with the discussion about longer periods upon transfer to constant light. We agree that the two processes are likely to be connected and have now placed them together in Ln 129-128.

5. Lines 650-652 of the Methods mentions that one of the main interests was the response to transfer to L:L, but this isn't mentioned in the introduction and doesn't come up much in the Results section. Most of the expression comparisons are focused on the 24-68h window. It also isn't clearly explained why the first day in LL is still a diurnal cycle. This would be helpful for non-circadian readers who may wonder why the first day is not included in all the analyses.

We believe this point is now also addressed by the addition of an Experimental Context section in the results (Ln 74-82), in response to the reviewer’s previous comment.

6. The phase comparisons shown in Figure suppl 4 are confusing. Suppl. Note 3 states that the period from the 24-68h data window was used to establish the bins but then the phase is shown for 3 different windows for each column? When calculating the phase for each of those 3 windows which period was used as the denominator in the phase calculation? Was it the period that matches the window used to calculate phase? What does the plot look like if phase is called on the same window used to calculate period (24-68)? What method was used to call phase in Suppl. Fig 4? As shown in Suppl Fig. 3 the method can influence the phase distributions. The methods suggest that the phase was determined with Metacycle but then FFT and MESA were used to verify. What does this mean verify, were they adjusted if FFT/MESA didn't agree?

We agree that this Figure was unnecessarily complicated. We have now simplified Supplementary Figure 4 so that only the phases from 24-68h are presented. We have also clarified the legend to explain why we used FFT-NLLS to improve accuracy of Metacycle predictions.

7. It is difficult to interpret the value of the period and phase comparisons shown in Fig. 1b, c, e and f after the preceding section about how variable the period and phase is across days. It is also surprising that the full 3 days were used to calculate the circadian statistics considering the first day is still under diurnal control. Do the ratios remain the same if the statistics are performed only on the 24h-68h window? For consistency with the rest of the paper and avoid confusion it would be best to have all circadian parameters measured using the same time window (24h-68h).

Thank you for your comments, we can see how our logic in using the different data windows was not clear enough. As mentioned above, we have now explained the use of the full and shortened data windows in Experimental context section (Ln 74-82). Fig 1c is a comparison between different circadian datasets and as such we have only compared periods across 24-68h window. Similarly, Fig 1b is a global analysis of periods in rhythmic genes in comparison with *Arabidopsis* and so is again measured from 24-68h. We have now clarified this in the Figure legend for 1b.

For comparisons of homoeologs within wheat triads, our question was in identifying homoeologs which behaved differently when placed under free-running conditions. We therefore still feel justified in using the full 0-68h dataset to identify homoeolog periods and phases which indicate differential circadian regulation, but we have now clarified that we are using the full dataset for the triad analysis in the results (Ln 140).

8. Fig 1h-m. How were those genes chosen? It would help to see the SD of the replicates shown, since this is just showing one triad. It would be helpful to see a plot that represents the full set of triads rather than just one that looks best. If normalized to a standard phase they could be put on the same plot. For example, panel j is meant to show the 8h lag of subgenome D. If the data is normalized so that A and B are set to the same phase all the triads could be displayed with shaded SD bars to show the variation. Something like this would be a better representation of the data rather than showing just one example.

Fig. 1h-m are case-studies illustrating the different forms of circadian imbalance between homoeologs. We agree that it is helpful to see the standard deviation as error bars on these triad plots and have added it as suggested. In line with another Reviewer 2’s suggestion we have removed Fig 1k and have replaced this with a comparison of mean normalised data for Triad 408 and Triad 2454, highlighting the difference between imbalanced rhythmicity and imbalanced amplitudes between homoeologs. Fig 1 I and m do not have error bars as adding standard deviations to mean normalised data wasn’t appropriate.

Thank you for your suggestion on how to display the different phases between homoeologs. We feel that if we were to plot all of the triads displaying imbalanced phases, the differences in period length and accompanying noise differences would make the plot so busy as to be unreadable. We hope that the pie charts Fig 1 d-g give a global overview of the proportions of triads with circadian imbalance, but agree with the point that it is useful to allow readers to view triads of their own preference. Therefore, we have now provided the replicate level TPM data with the triad IDs annotated (Supplementary File 12) and Supplementary file 11 provides the classification of each triad alongside Metacycle statistics, ortholog identification and cluster information discussed elsewhere in the paper. Readers can now look up a triad or gene of interest and see how it was classified and what the expression looks like over the full dataset.

9. It is surprising that there aren't more comparisons with the B. rapa dataset, especially when discussing the clock genes that show balanced or imbalanced expression. Are they similar in B. rapa and does it support your hypothesis that unbalance for certain genes are selected against?

While we agree that a thorough, multiple species, comparative transcriptomic analysis is undoubtably of interest for the future, we feel it is beyond the scope of the questions being addressed in this paper. We do compare paralogs defined as “similar” in the Greenham dataset with homoeologs described as “balanced” in our dataset and find that genes involved with “photosynthesis” and “generation of precursor metabolites and energy” tend to be common between the two groups, potentially suggesting conservation of balance for certain types of genes (Ln 206-217).

10. Figure 2 networks. Why were these specific modules selected? Is it actually appropriate to directly compare these modules? I do see that some of the comparisons have high correlations from panel a, but not all. For example, in panel b the W9 and A9 modules have a correlation value of 0.92, which seems appropriate. However, panel c (modules W3 and A2) have a correlation of 0.42, which seems far too low to make any sort of comparison meaningful.

The modules were selected to simplify the comparison of genes expressed in the dawn, midday, dusk, and night. We were interested in identifying common GO-enrichment in genes peaking throughout the day, although as you have identified, the differences in period length between *Arabidopsis* and wheat made this difficult. Our reasons for comparing module W3 with module A2, were that, even though their eigengenes are not highly correlated per se, when period length is taken into account, both modules peak during the subjective day (CT 6.34h and 6.19h) and they share commonly enriched GO terms which make sense for day peaking genes.

Further, as described in methods comments, using a cutHeight as low as 0.15 will likely lead to some number of genes in any given module that do not necessarily "share" a similar expression pattern. These genes could have a pattern that has very low correlation to their module eigengene and were only placed in that module because the pattern was "less similar" to other module eigengenes. The current expression plots in this figure follow a clear pattern, but I suspect this would be even more apparent if the genes within these modules had a higher correlation to the module eigengene. Perhaps the current genes in these modules could just be filtered to have a higher correlation score?

Thank you for your comments, we have now made changes to the Results and Methods to clarify our approach (Ln 237-239 and Ln738-765). Merging modules with highly correlated module eigengenes (ME) is the final step in constructing our co-expression networks. To do this, as the reviewer describes - we used the WGCNA default parameter of a mergeCutHeight() of 0.15. This results in the merging of modules with highly correlated ME as the 0.15 mergeCutHeight() refers to the dissimilarity metric of 1 minus the eigengene correlation. So for WGCNA, a mergeCutHeight() of 0.15 corresponded to a correlation of 0.85. For the wheat modules, we took the additional step of merging closely related modules (mergeCloseModules()) using a cutHeight of 0.25, again a dissimilarity metric of 1 minus the eigengene correlation (corresponding to a correlation of 0.75). Reducing the stringency of the cutHeight to merge highly correlated wheat modules enabled us to more easily compare significantly correlated wheat and *Arabidopsis* co-expression modules to identify groups of genes in wheat and Arabidopsis expressed at similar times in the day, and enable the comparison of whether similar phased transcripts in wheat and Arabidopsis had similar biological roles.

11. Lines 327-334: I am not following the connection between 'response to abiotic stimulus' and the photoreceptor and light signaling proteins. At the start of this section (line 308) the authors say that the GO analysis was only done on rhythmically expressed genes but the reference to only one PHYA being rhythmic and yet multiple genes are shown in the plot in fig. S16. Does this mean that all the genes were shown and not just the rhythmic ones? This would explain why many of the PHY and CRY genes don't seem to have rhythms. This should be clarified better in the text or indicated in the plot which ones were called rhythmic. Since the first day following transfer is still the diel pattern from the entrainment condition, what does the PHY and CRY expression look like? Does it appear rhythmic under diel but lose rhythmicity in LL? It should be noted in the text that arrhythmicity in circadian conditions doesn't mean there isn't rhythmicity under diel conditions. This could be an additional explanation apart from the current one in the text that the regulation is at the level of protein stability/localization. Overall, this entire section is very long and entirely based on data shown in the supplemental material. I do appreciate having the individual gene plots that supplement Figure 4 and would suggest either providing a main figure to highlight a small subset of genes or pathways in this section or shorten it and focus on the results shown in the main figures.

Upon reading the reviewer’s comment, we realize that we should have made our motivations and processes clearer within this section. We used the data filtered for rhythmicity to conduct the GO-enrichment analysis and then used that to identify processes which should be of interest for further investigation. We have now added an additional sentence (Ln 352-354) to explain this more clearly. We then considered the orthologs of well-known *Arabidopsis* gene networks and extracted their expression from our circadian dataset, whether rhythmic or not. Supplementary Table 10 contains all of the genes we investigated, their expression and their MetaCycle statistics. We have also indicated here which genes are plotted in which Supplementary Figure 18-20. The reasons for plotting non-rhythmic genes in some cases was that it illustrates the differences between circadian control in *Arabidopsis* versus wheat (as is the case for the PHY and CRY genes). We understand that it is useful to see at a glance which genes are classified as rhythmic or arrhythmic, so have now highlighted each row in Supplementary Table 10 to make this more intuitive, and added a read me tab.

Regarding your point about oscillation under diel cycles, we agree that some transcripts will show rhythmic behaviour under entraining environments but not under constant conditions, and may perform time-of-day specific functions. However, these transcripts are likely to not be regulated by the circadian clock (at the transcriptional level) and so are not discussed in the context of a circadian transcriptome.

For your interest, here is the full expression of PHY and CRY transcripts starting at ZT0:

It is difficult to say for definite, but it seems likely that some of these photoreceptors will have rhythmic patterns of expression under diel cycles, but these rhythms do not endogenously persist under constant conditions.

We appreciate your feedback that this section would benefit from cutting down of text and addition of a Figure to illustrate the text. We have now cut some of this section down and created a new main figure based on some of the oscillation plots from Supplementary Figure 18 and 19. We chose examples that reflect a conservation of relationships between transcripts of different peak phases, as we find it interesting that both species have similar patterns. (Main Figure 4, Ln 361--363, 382).

12. Primary metabolism section: in terms of the supplemental figure, similar to the previous one I think it would declutter the plots if the genes that are not rhythmic were left out and simply indicate below the plot that they didn't meet the rhythmicity cutoff. This is another area where there is more discussion surrounding the supplemental figures than the main figure 4.

One of the overall findings of this section was that many of the genes involved in Starch and T6P metabolism which are rhythmically expressed in *Arabidopsis* are not rhythmically expressed in wheat. We feel removing these genes from the results would detract from the importance of this finding. We have now edited Supplementary Table 10 to highlight which genes are classified as rhythmic. We have also added in a sentence to the start of this section which lays out our motivations for this analysis, summarises our findings and better connects the text with an explanation of Fig. 5 (Ln 408-430).

13. For all gene expression figures there should be SD or SE shown either as bars or ribbons to represent the variation in replicates.

Although we agree that error bars are informative for showing variation between replicates (and have added them to Fig. 1 to show differences within wheat triads) we feel that adding error bars to the gene expression plots in Fig. 3, Fig 4 and Supplementary Fig 19-20 would make these plots difficult to read, particularly where the wheat homeologs are very similar. The purpose of these gene expression plots is to compare circadian profiles in *Arabidopsis* and wheat orthologs rather than to claim significant differences in expression at any particular timepoint. This is fairly common in other circadian biology studies:

<https://www.pnas.org/doi/10.1073/pnas.1408886111> ,

<https://www.jbc.org/article/S0021-9258(17)49454-3/fulltext#seccestitle20> , <https://journals.plos.org/plosone/article/comments?id=10.1371/journal.pone.0169923> , <https://www.science.org/doi/10.1126/science.290.5499.2110?url_ver=Z39.88-2003&rfr_id=ori:rid:crossref.org&rfr_dat=cr_pub%20%200pubmed>,

<https://www.frontiersin.org/articles/10.3389/fgene.2021.664334/full>,

<https://www.science.org/doi/full/10.1126/science.1161403>

The replication level information for each gene has now been made available in Supplementary file 12.

14. It would be very helpful to include the code used to generate the networks and perform the cross-correlation of eigengenes across networks should be included in the Methods. This will also save you from responding to email requests!
Thank you for your comment, Code for the cross-correlation analysis, Loom plots and WGCNA network construction is now available from our groups GitHub repository: <https://github.com/AHallLab/circadian_transcriptome_regulation_paper_2022/tree/main>

Minor Comments

1. Figure 1, panel d: - The "unbalanced" triads that are depicted by the lighter shading; do these in fact have a different cutoff than the original rhythmic homoeologs? In the figure it says q<0.1 but I thought it was q<0.01.

Thank you for bringing this to our attention, this has now been corrected.

2. Hard to directly compare the GO term overlap in Figure 2f. Might be better to only show the results for the 4 pairs shown in b-e and put them side by side in the bubble plot.

Thank you for this feedback, We have tried to make this plot easier to understand without losing any of the available information. Hopefully it is now more intuitive to understand which columns are being compared. We have changed the coloured lines to make them slightly wider, put the modules in corresponding coloured boxes and highlighted GO-slim terms shared by modules being compared.

3. Line 314 -316 don't see supp tables 10, 11

Our apologies, these files were missed previously from the upload are now available.

4. For the selection of B. rapa circadian paralogs with similar and differential expression patterns (starting line 714), the authors choose a hard cut off of 0.001 (differentially patterned) OR 0.1 (similarly patterned). What happens to the genes that are between these two cut offs or is this a typo. Since all the other cutoffs for rhythmicity was set at 0.01 it seems likely that this is a typo.

We have now clarified this in the methods, (Ln 807-822). This is not a typo, but it is a different method to the Metacycle approach we have used for our wheat data. We defined similar/different paralogs as characterized in Greenham et al, (2020) using DiPALM p-values. We chose these DiPALM p-value cut-offs as they gave us approximately equal numbers of paralogs in each category, which represent tails of similarly expressed or differently expressed circadian genes. We checked these cut-offs by calculating average Pearson’s correlation statistics between paralogs and found that differential Brassica paralogs had a mean Pearson correlation coefficient of 0.31 (SD = 0.43) and similar Brassica paralogs had a mean Pearson correlation of 0.75 (SD= 0.23) which confirms that the DiPALM method of defining expression patterns makes sense in the context of this analysis.

5. Line 681. Should be supplemental Figure 6 not 9.
6. References to most supplemental figures are not the correct number.
7. Labels above the plots in Supp Fig5 do not match the legend.

We apologise for these mistakes. We realize that we had mistakenly submitted an earlier draft of the Supplementary materials file, which was missing Supplementary Figure 5, 6 and 9 which therefore shifted the order of the remaining figures. This is now updated.

8. Suppl table 7 should be as a separate .csv file or similar to be able to see the full table.

This is a good suggestion, and we have added this.

9. Line 723 should be B. rapa not B. napus.

Thank you for catching this! Corrected.

10. Figure 4. There is no explanation for what the black boxes represent in the figure legend.

Thank you for your comment. Figure 4 (new Figure 5) has now been updated.

Reviewer #1 (Significance (Required)):

This study provides new insight into the circadian regulation of the transcriptome in a new allopolyploid. It adds a valuable resource to a growing collection of circadian studies in important crops and will greatly improve our efforts to learn more about the circadian control of important crop improvement traits. The dataset will be of interest to other plant circadian biologists as well as the general plant biology community who focus on monocot crops. My expertise is more on the transcriptomic side and I do not have the expertise to evaluate the phylogenetic work presented in this study.

**Reviewer #2 (Evidence, reproducibility and clarity (Required)):**

Summary
Rees et al. present an RNAseq time course of bread wheat. Its recent polyploidisation is one motivation for this study as gene expression dosage is known to be important for clock function in other plants.
The time course covers 3 days at sampling intervals of 4h of 2-week old wheat plants (all aerial tissues), in triplicates. The subsequent analysis of the RNAseq data includes analysis of the generated data by itself (e.g. GO analysis, rhythmicity, period and phase analysis, rhythmicity of transcription factor families as well as TF binding sites) as well as thorough comparison with published datasets of other species (Arabidopsis, Brassica rapa, Brachypodium dystachion). One of the key findings is that the mean period length and the period spread are larger in wheat than in these other species). Circadian clock genes largely have similar dynamics in wheat compared to Arabidopsis.
In addition, one focus is the analysis of the dynamics of three genes of one triad and imbalance / balance of such triads. To the surprise of the authors, circadian regulated and clock genes were not necessarily balanced. Silencing is one of their explanation for imbalance of circadian genes as arrhythmic genes of one triad are typically those with the lowest expression level. Finally, the authors point out more examples of rhythmic processes and genes (photoreceptors and signalling, auxin, carbon metabolism) and their commonalities and differences with Arabidopsis.


Major comments
- The key conclusions and the data are convincing

We thank the reviewer for their supportive comment.

- line 120 and figure 1: In my opinion, q > 0.05 is not a good definition of arrhythmicity as non-significant q-values can result from either noise in spite of rhythmicity or from arrhythmicity. A more statistically sound way to detect arrhythmicity could for example be two-one-side tests (for example in the R package 'equivalence', e.g. see usage for time courses by Noordally et al. 2018, <https://www.biorxiv.org/content/10.1101/287862v1>).

Thank you for pointing us in the direction of this package, we agree that choosing methods for circadian quantification and q-value cut-offs is always tricky and different approaches will perform better for noisier or non-sinusoidal waveforms. For future work, we will investigate the application of the suggested method in circadian rhythmicity analysis. However, we believe that the criteria used in this paper for rhythmicity quantification is suitable for addressing our questions, and overall, we are satisfied that rhythms with a q-value of >0.05 would also be classified by eye as being arrhythmic, and rhythms with a q-value <0.01 would be classified as rhythmic.

Many other studies have used meta2d B.H q-values as a metric of rhythmicity: e.g. (<https://bmcplantbiol.biomedcentral.com/articles/10.1186/s12870-022-03565-1> , <https://link.springer.com/content/pdf/10.1186%2Fs12915-022-01258-7> , <https://www.ncbi.nlm.nih.gov/pmc/articles/PMC8782462/pdf/pcbi.1009762.pdf> )

- lines 480-484 and intro: In the introduction, the authors write that expression levels of clock components are important for the function of the clock, and that this is one motivation for the current study where polyploidisation is expected to affect the expression levels of clock genes and their outputs. I wonder what answers or speculations this study provides in the end, or whether such answers / speculations should be made clearer. For example, do the authors think that the higher variability of periods in wheat could be a consequence of lower robustness (in addition to possible spatial differences that are mentioned) due to polyploidisation? Is anything known about the period of rhythms of close wheat relatives that did not undergo polyploidisation? Did you look at dampening over the time course in wheat vs. Arabidopsis?

The point above is an interesting one, and we thank the reviewer for raising it. We agree that the high variability of periods in wheat may be a product of polyploidisation, as functional redundancy between homoeologs may allow a tolerance for less tightly regulated, non-dominantly expressed circadian transcripts. We have now added this hypothesis to our discussion: Ln536-550.

In our comparative analysis of period distributions, we looked at periods of transcripts from a diploid relative of hexaploid wheat, *Brachypodium distachyon.* In *Brachypodium,* period lengths have around the same SD as in *Arabidopsis* but the mean period length is slightly longer (Supplementary table 2). We have now edited our results to make the relationship between wheat and *Brachypodium* clearer (ln 109-110).

Minor comments:

Introduction
- lines 49: it is unclear what is meant by ppd-1 at this position of the sentence

We agree this was unclear and have revised it to “notably the *ppd-1* locus within *TaPRR3/7”* Ln 52

- line 54/55: clarify that this refers to Arabidopsis thaliana
Corrected.

Results
- line 69 and 76: cite references for these tools here (not only in the methods section)

Corrected.

- line 90-93: Why wouldn't the same thing happen on subsequent subjective evenings?

Thank you for your comments. We have now combined our hypothesis for why wheat transcripts expressed at dusk have longer periods with the discussion about longer periods upon transfer to constant light. We think that the two processes are likely to be connected and have now placed them together in Ln 126-131.

The behaviour of mean period lengths of wheat transcripts upon transfer to constant light was unexpected and we believe is quite interesting. One explanation is that the influence of the ongoing light zeitgeber when dusk was expected causes a delay in the expression of evening peaking genes which are delayed by the continuous light signal. Then, on subsequent evenings the influence of the diel dusk signal is ‘forgotten’ as the governance of the endogenous clock takes over. The very long period observed at 0-24h (28.61h) may be due to a phase shift rather than an intrinsic lengthening of period per se. Whether this trait is unique to wheat or can also be seen in other plant species is, to our knowledge, unknown.

- line 118: what is your defined cutoff for significance of the Chi square test (p=0.03 not regarded significant?)

The reviewer is completely right, we have now clarified this. Ln 145-149

- figure 1h,i: In order for the reader to see whether A and D (Figure 1h) or A (figure 1i) are indeed arrhythmic, one would need to see plots with a normalisation as done in figure 1m for 1l.

We have now removed the triad showing one rhythmic gene and two arhythmic genes (as Fig. 1h already illustrates this type of circadian imbalance) and replaced this with a side by side comparison of how imbalance in rhythmicity differs from imbalance in relative amplitude as suggested.

- figure 1h-m (and others with circadian time course traces): could a measure of variation (e.g. SD, SEM, confidence interval) be plotted as a shaded region around the curves (unless they're so small that they are there but not visible)?

We have now added error bars to these plots to show standard deviation between replicates, in Fig. 1 h, j, k and l. We could not think of an accurate way to display this information for the mean normalised data (Fig 1. i and m) so have not put error bars on these plots.

- line 139 (also in 737 and 450): give reference to Ramirez-Gonzalez et al in the same style as the rest of the manuscript (number)

Thank you for raising this, we believe we have corrected all in-text citations (both narrative and fully parenthetical form) for consistency with the APA format used by the majority of Review Commons affiliate journals.

- Clustering (modules): What is the reason for choosing 9 clusters? Was this number optimised or chosen for other reasons?

WGCNA uses an unsupervised clustering algorithm that works within the supplied parameters to determine the optimum number of clusters to explain the dataset, without prior specification of the number of clusters. We have amended the manuscript text to clarify this Ln237-239.

- lines 280 - 284: The TaELF3-1D phenotype could be explained a bit better to the non-wheat specialist, for example by mentioning in the beginning of this set of sentences.

Done (Ln 314-318).

- The authors present an analysis of TF binding sites. Can they say something about binding sites in a less sophisticated manner, such as on some very well-known motifs in promoters like the evening element?

We agree that this is a very interesting question, and one that we may investigate in more detail with our data in the future. In this paper, we performed a global analysis of wheat TFBS predicted from orthologous *Arabidopsis* TF targets. These targets have been experimentally validated in *Arabidopsis* using DAP-seq, but we have not validated that these binding sites exist in wheat promoters. We therefore took a tentative approach, and presented only enrichments at the superfamily level rather than talking about specific regulatory motifs.

The evening element would fit most likely fit within the MYB or MYB-related TFBS superfamily, however the diversity of transcription factors in this family means that there is significant enrichment of these TFBS in multiple modules throughout the day (Supplementary Figure 11). In summary, a more in depth TFBS analysis of known circadian motifs is of great interest, but we feel would be a substantial work in its own right.

- Figure 1h-l: If known or meaningful, it would be interesting to know the gene identities behind the triads shown, as in supplementary figure 5.

These triads were selected as case studies to exemplify the ways in which we were defining imbalanced circadian triads. They have no particular relevance to the figure, but out of curiosity, these are the closest *Arabidopsis* orthologs for the triads displayed in Fig. 1:

Triad 408 has highest identity to a hypothetical protein (AT4G26415).

Triad 2454 is similar to AT3G07600, a heavy metal transport/detoxification superfamily protein

Triad 13405 is similar to AT3G22360, encoding an ALTERNATIVE OXIDASE 1B, AOX1B

Triad 10854 is similar to NSE4A, a δ-kleisin component of the SMC5/6 complex, possibly involved in synaptonemal complex formation (AT1G51130).

Information about wheat gene names in each triad and their *Arabidopsis* orthologs can be viewed in Supplementary Table 11, so that readers can search for genes of particular interest to them.

- Figure 4 and text: The illustration of starch metabolism is very helpful. However, I think the paper would benefit from giving a better reason for the selection of this specific set of processes, for example by relating these findings to functional differences in starch metabolism in the two species (in contrast to Arabidopsis, wheat stores little starch in leaves but uses fructans as main reserve carbohydrate)? Are there known differences in the dynamics of starch degradation during the night?

The reviewer raises an interesting point, and we have now clarified in our results that the stated differences between starch regulation in *Arabidopsis* and wheat was part of the motivation behind studying this pathway. Starch is at the centre of plant primary metabolism as a carbon storage source and is arguably one of the most important features that breeders look for in regard to grain filling and yields. Additionally, it is of interest to circadian biologists as starch (as well as sucrose) have been shown to transiently cycle and to be regulated by the circadian clock. However, in wheat, carbon storage primarily uses sucrose rather than starch, and we have now added sucrose to Figure 5 to place it in this context. We think your suggestion has now improved our explanation for why we focused on starch in the manuscript, and we are grateful for your input (Ln 408-421).

We also agree that the differences in the ways that *Arbaidopsis* and wheat utilise starch versus sucrose, and perhaps the role that fructans have in as a reserve carbohydrate and in protection against freezing in wheat may be one of the reasons we are seeing differences in circadian regulation of starch. We have now added this to our discussion (Ln 584-592).

- Figure 4: triose-phosphates can be transported in and out of the chloroplast, as is illustrated in the figure. However, the illustration looks as though they are converted to hexose phosphates during the transport process. In order to be consistent with other transport processes of the figure (maltose and glucose), triose-phosphate should be repeated on the cytosolic side.

We have now amended this (new Fig. 5). Thank you for your feedback.

Methods
- line 543: if I understand correctly that triplicates were collected and analysed for each time point, '18 samples' is mis-leading (18 time points would be more accurate).

We agree this was badly worded. Changed Ln 615.

Supplementary
- Supplementary figure 3: x axis label very small and contains typo

Now corrected. Also enlarged axis for Supplementary Figure 2.

- Supplementary table 1: Romanowski et al 2020 (add year), or use ref. number citation style as in the rest of the manuscript

Thank you for raising this, we have now hopefully corrected all in text citations (both narrative and fully parenthetical form) to be consistent with APA format used by the majority of Review commons affiliate journals.

- Supplementary table 9, primary metabolism: does bold highlighting of Arabidopsis accession numbers have a meaning or is it accidental?

We apologise that this was unclear. We have corrected this. Supplementary Table 10 now also has a “Read me” tab which explains that table.

Reviewer #2 (Significance (Required)):

I believe this is a precious, carefully generated and analysed dataset which many biologists will benefit from, beyond wheat or circadian specialists.
The dataset expands the knowledge of circadian transcriptome regulation to an important crop and contributes a resource of which only a handful of others exist in other species.
Many high impact papers on RNAseq include some follow-up on candidates, for example in Romanowski et al 2020, which is admittedly easier to do in Arabidopsis than wheat due to the availability of genetic resources.

My expertise: Plant circadian clock (Arabidopsis), dataset analysis (but not specifically for RNAseq)


**Reviewer #3 (Evidence, reproducibility and clarity (Required)):**

This manuscript is based on the analysis of a single experiment consisting in transcriptomic profiling of one (hexaploid) wheat genotype along 3 days (samples taken every 4 hours). The experiment is performed in constant light conditions, allowing detection of transcripts controlled by the circadian clock. The bioinformatic analysis studies the dynamics of the different homoeologous transcript in the polyploid genome and compares cycling transcripts in wheat with what is known from Arabidopsis.

The manuscript is well written, the methods are correct, the analysis performed is sufficiently extensive and the figures are clear. The manuscript finds interesting expression patterns among homeologous genes, and goes into detail on important differences in circadian regulation of relevant gene families between Arabidopsis and wheat. The work is purely descriptive and does not aim at associations with physiological phenotypes, but the bioinformatic analysis is very thorough and uncovers interesting examples.

Only one caveat: For what I gather, there is no replication in the RNA-seq experiment, although the exact method does not appear in the text. From the Methods section: "tissue was sampled every 4h for 3 days (18 samples in total)" and "At each timepoint, we sampled the entire aerial tissue from 3 replicate plants". Whether these samples were pooled or not is not described. The "Data Availability" section links to 18 RNA-seq paired end libraries, which suggest that the replicates were pooled, although some type of barcoding might have been used. The text should mention if the replicates were pooled or not, and, if so, what was the method used for poling (tissue, RNA or libraries). Even in the case of no biological replication the manuscript brings interesting insights into wheat transcriptomics and circadian biology. The editor (or the rules of the journal) should decide if they accept articles with no "real" biological replication (I am sure we all understand by now the benefits and limitations of pooling biological replicates into a single RNA-seq library).

There was replication within the RNA sequencing experiment, and we apologise that this was unclear from our manuscript. Each timepoint consisted of three independent biological replicates. We have now created a new “Experimental context” section in the results to explain this (Ln 74-82) and have clarified in the methods how our data was processed (Ln 609-615 and 636-638).

We have now included an additional matrix with TPMs at the replicate level to assist readers in looking at specific genes of interest (Supplementary Table 12).

Minor comments:

The description of the experimental setup in the first sentence of the Results section is too brief. Could you please talk about for how long the experiment was running? At what intervals the samples were taken? What conditions were used?

We apologise that this was unclear. We hope that the new Experimental Context section, added in response to comments from several reviewers, makes this much clearer, alongside the clarification in the methods (Ln 609-615 and 636-638).

Line 280: "...due *to* an introgression..."

Corrected. Ln 315

The legend of Figure 3l says elf4 instead of elf3

We thank the reviewer for noticing this mistake that we have now corrected.

Line 306 "says Supplementary Note 7 instead of Supplementary Note 7

We are not sure what is to be corrected here!

Reviewer #3 (Significance (Required)):

This works advances our knowledge on how genome wide expression levels are controlled by the circadian clock in polyploids. Although previous works had performed similar analyses in other polyploid plants, this is the first time this is done in an hexaploid. This work is a starting step to understand gene regulation in this important crop, and have interest for researchers working in fundamental and applied plant biology.

Thank you for your positive comments and your feedback in improving this manuscript. We would like to clarify that to our knowledge, this work presents the first analysis of a circadian transcriptome in a polyploid crop. The work by Greenham et al, although undoubtably providing insight into circadian regulation of ancient paralogs, was performed in the diploid *Brassica rapa*.
