# Supplementary material for: Circadian regulation of the transcriptome in a complex polyploid crop
Source: PLoS Biol. 2022 Oct 13;20(10):e3001802. doi: 10.1371/journal.pbio.3001802 (PMC9560141; doi:10.1371/journal.pbio.3001802)
Supplement: Attachment — Submitted filename: Response_to_review_AUG22.docx [file pbio.3001802.s045.docx]

Response to reviewers- comments from authors are written in blue

Dear Dr Hall,

Thank you for your patience while we considered your revised manuscript "Circadian regulation of the transcriptome in a complex polyploid crop" for publication as a Methods and Resources at PLOS Biology. This revised version of your manuscript has been evaluated by the PLOS Biology editors, the Academic Editor and the original reviewers from Review Commons.

You can read the reviewer comments below my signature. As you will see, the reviewers feel the revision has largely addressed their original concerns and they highlight that this is a valuable dataset. However, the reviewers have identified a number of minor issues which we think should be addressed before publication, in a revision that we think will not take very long.

Based on the reviews, we are likely to accept this manuscript for publication, provided you satisfactorily address the remaining points raised by the reviewers.

**IMPORANT: In addition to addressing the reviewer comments please also make sure to address the following data request:

--
DATA POLICY REQUEST:

You may be aware of the PLOS Data Policy, which requires that all data be made available without restriction: <http://journals.plos.org/plosbiology/s/data-availability>. For more information, please also see this editorial: <http://dx.doi.org/10.1371/journal.pbio.1001797>

Note that we do not require all raw data. Rather, we ask that all individual quantitative observations that underlie the data summarized in the figures and results of your paper be made available in one of the following forms:

1) Supplementary files (e.g., excel). Please ensure that all data files are uploaded as 'Supporting Information' and are invariably referred to (in the manuscript, figure legends, and the Description field when uploading your files) using the following format verbatim: S1 Data, S2 Data, etc. Multiple panels of a single or even several figures can be included as multiple sheets in one excel file that is saved using exactly the following convention: S1_Data.xlsx (using an underscore).

2) Deposition in a publicly available repository. Please also provide the accession code or a reviewer link so that we may view your data before publication.

Regardless of the method selected, please ensure that you provide the individual numerical values that underlie the summary data displayed in the following figure panels as they are essential for readers to assess your analysis and to reproduce it:
Fig 1b,c,h-n; Fig 2b; Fig 3a-m; Fig4a-f; Fig S1a-c; Fig S2a-f; Fig 3a-l; Fig S4; Fig S5; Fig S6; Fig S7; Fig S9; Fig S10; Fig S11; Fig S17a-m; Fig S18a-d; Fig s19a-h; Fig S20a-n

NOTE: the numerical data provided should include all replicates AND the way in which the plotted mean and errors were derived (it should not present only the mean/average values).

We have provided the underlying data for all figures listed. Data for figures in the main text are in S1_Data.xlsx and data for figures in Supplementary are in S2_Data.xlsx. Replicate level data, means, standard deviations, and mean-normalised data are provided where appropriate.

>>Please also ensure that figure legends in your manuscript include information on where the underlying data can be found, and ensure your supplemental data file/s has a legend.

The supplemental data files have a read-me tab and all individual data tabs have their own legend. All supplementary data is referenced in the figure legends in the manuscript.

>>Please ensure that your Data Statement in the submission system accurately describes where your data can be found.
The Data availability section has been updated to include the new files

As you address these items, please take this last chance to review your reference list to ensure that it is complete and correct. If you have cited papers that have been retracted, please include the rationale for doing so in the manuscript text, or remove these references and replace them with relevant current references. Any changes to the reference list should be mentioned in the cover letter that accompanies your revised manuscript.

We updated one citation to update its publication in a peer reviewed journal: Cuitun-Coronado et al., 2022

**We expect to receive your revised manuscript within two weeks. However, if you need additional time to address the reviewer concerns, please do let us know and we are happy to grant an extension.**

To submit your revision, please go to <https://www.editorialmanager.com/pbiology/> and log in as an Author. Click the link labelled 'Submissions Needing Revision' to find your submission record. Your revised submission must include the following:
-  a cover letter that should detail your responses to any editorial requests, if applicable, and whether changes have been made to the reference list
-  a Response to Reviewers file that provides a detailed response to the reviewers' comments (if applicable)
-  a track-changes file indicating any changes that you have made to the manuscript.

NOTE: If Supporting Information files are included with your article, note that these are not copyedited and will be published as they are submitted. Please ensure that these files are legible and of high quality (at least 300 dpi) in an easily accessible file format. For this reason, please be aware that any references listed in an SI file will not be indexed. For more information, see our Supporting Information guidelines:
<https://journals.plos.org/plosbiology/s/supporting-information> 

*Published Peer Review History*
Please note that you may have the opportunity to make the peer review history publicly available. The record will include editor decision letters (with reviews) and your responses to reviewer comments. If eligible, we will contact you to opt in or out. Please see here for more details:
<https://blogs.plos.org/plos/2019/05/plos-journals-now-open-for-published-peer-review/>

*Press*
Should you, your institution's press office or the journal office choose to press release your paper, please ensure you have opted out of Early Article Posting on the submission form. We ask that you notify us as soon as possible if you or your institution is planning to press release the article.

*Protocols deposition*
To enhance the reproducibility of your results, we recommend that if applicable you deposit your laboratory protocols in protocols.io, where a protocol can be assigned its own identifier (DOI) such that it can be cited independently in the future. Additionally, PLOS ONE offers an option for publishing peer-reviewed Lab Protocol articles, which describe protocols hosted on protocols.io. Read more information on sharing protocols at <https://plos.org/protocols?utm_medium=editorial-email&utm_source=authorletters&utm_campaign=protocols>


Please do not hesitate to contact me should you have any questions.

Sincerely,

Luke

Lucas Smith, Ph.D.
Associate Editor,
lsmith@plos.org,
PLOS Biology


------------------------------------------------------------------------
Reviewer remarks:

Reviewer #1: I appreciate the work done by Rees et al. to address and respond to my concerns from the first review. This dataset will be a fantastic resource for the community and will hopefully inspire others to perform circadian studies in other crops as well as other cultivars within a species.

I have a few minor comments to improve clarity in the text:
1. In figure 1, panels l-m. It would be helpful to have the color legend directly on the figure to easily identify the subgenomes.

We have now included this as suggested.
2. This is just a comment, but I love supplemental figure 6 and think this is really cool and a great way to emphasize the importance of time course data and being carefully about assessing subgenome dominance from single time point (or single condition) studies.

Thank you for the feedback!
3. For Figure 3 and supplemental figure 17, it is difficult to make statements about expression level when there is no statistical support. Given that these are normalized plots without SD showing the variation I would suggest either running a test or de-emphasizing the expression level and focusing more on the period and phase.

We have edited this as suggested. Ln 322-324 now has the statistical test for mean expression level and we have removed mention of expression level on Ln 330.
4. On line 383 referring to HFR1 expression the Figure reference should be 4e not 4c.

Corrected, thank you.
5.  The results section on starch metabolism has some redundancies that could be eliminated with a little reorganizing. Currently, starch regulation is explained at the start of the section and then there is a shift to discussing Rubisco and then returning to starch synthases. It would improve flow to bring all the relevant starch metabolism text together.

After considering this suggestion, we have decided to keep the existing structure as the opening paragraph introduces the background to circadian control of primary metabolism genes in wheat and Arabidopsis and describes the overall message of Figure 5. The following paragraphs then break this down into further description of the individual components. We have now added a new sentence which clarifies the separation from this overview to the breakdown of components.

Ln 433; A more detailed analysis of the individual components in this network is described below.


Reviewer #2: The authors have addressed all of my comments and concerns sufficiently.
In one of the edits a parenthesis needs closing (line 146).
As stated in my first review of this paper on Review Commons I believe that this is a very valuable, and carefully constructed resource to the circadian biology community and beyond, including crop improvement.
Thank you for your time in reviewing our manuscript.

Reviewer #3: After revision by the authors, I have no objections to the manuscript.
Thank you for your time in reviewing our manuscript.
__________________________________________________
In compliance with data protection regulations, you may request that we remove your personal registration details at any time.  (Use the following URL: <https://www.editorialmanager.com/pbiology/login.asp?a=r)>. Please contact the publication office if you have any questions.
